# A Bias‐Corrected Bayesian Nonparametric Model for Combining Studies With Varying Quality in Meta‐Analysis

**DOI:** 10.1002/bimj.70034

**Published:** 2025-02-07

**Authors:** Pablo Emilio Verde, Gary L. Rosner

**Affiliations:** ^1^ Coordination Center for Clinical Trials University Hospital Dusseldorf Heinrich Heine University of Dusseldorf Dusseldorf Germany; ^2^ Division of Quantitative Sciences Johns Hopkins University Baltimore Maryland USA

**Keywords:** Bayesian nonparametric, bias correction, comparative effectiveness methods, conflict of evidence, cross‐evidence synthesis, hierarchical models, meta‐analysis

## Abstract

Bayesian nonparametric (BNP) approaches for meta‐analysis have been developed to relax distributional assumptions and handle the heterogeneity of random effects distributions. These models account for possible clustering and multimodality of the random effects distribution. However, when we combine studies of varying quality, the resulting posterior is not only a combination of the results of interest but also factors threatening the integrity of the studies' results. We refer to these factors as the studies' *internal validity biases* (e.g., reporting bias, data quality, and patient selection bias). In this paper, we introduce a new meta‐analysis model called the bias‐corrected Bayesian nonparametric (BC‐BNP) model, which aims to automatically correct for internal validity bias in meta‐analysis by only using the reported effects and their standard errors. The BC‐BNP model is based on a mixture of a parametric random effects distribution, which represents the model of interest, and a BNP model for the bias component. This model relaxes the parametric assumptions of the bias distribution of the model introduced by Verde. Using simulated data sets, we evaluate the BC‐BNP model and illustrate its applications with two real case studies. Our results show several potential advantages of the BC‐BNP model: (1) It can detect bias when present while producing results similar to a simple normal–normal random effects model when bias is absent. (2) Relaxing the parametric assumptions of the bias component does not affect the model of interest and yields consistent results with the model of Verde. (3) In some applications, a BNP model of bias offers a better understanding of the studies' biases by clustering studies with similar biases. We implemented the BC‐BNP model in the R package jarbes, facilitating its practical application.

## 1 Introduction

Meta‐analysis methods help researchers answer questions that require the combination of statistical results across several studies. Very often the only available studies are of different types and with varying quality. Therefore, when we combine disparate evidence at face value we are not only combining results of interest but also biases that might threaten the quality of the results. As a consequence, the resulting meta‐analysis could be misleading.

Factors threatening the integrity and quality of studies are called *internal validity biases*. For example, patient selection bias, dilution bias, reporting bias, data quality, and so forth are all forms of internal validity bias. The problem of these types of biases is that they are not directly observable, making their correction in meta‐analysis a challenging problem.

Table 1 presents an example of a meta‐analysis combining different types of studies, where researchers collected evidence across 18 observational studies (OS) on COVID‐19‐infected patients (de Almeida‐Pititto et al. 2020). This meta‐analysis was performed during the COVID‐19 pandemic when researchers were urgently trying to assess evidence linking baseline risk factors, such as in this case, hypertension, with mechanical respiratory assistance within 28 days. We will present more details about this meta‐analysis in Section 4, but by looking at the studies' summaries and the different design types, we have what we call the “Anna Karenina” principle in evidence synthesis: *good quality studies are alike, but biased ones are biased in their own way*. However, these studies were the only available evidence at that time, and researchers faced an imperfect body of evidence to answer their question.

**TABLE 1:** Example of a meta‐analysis combining disparate studies, where researchers collected evidence across 18 observational studies on COVID‐19‐infected patients. The odds ratio (OR) measures the association between hypertension and mechanical respiratory assistance within 28 days after hospitalization. Values of OR greater than one indicate higher risk of hypertension.

Author	Design	OR	seOR	N
Guo et al. 2020	Case series	6.48	1.43	187
Li J et al. 2020	Case series	2.59	1.14	1178
Mao et al. 2020	Case series	3.17	1.39	214
Wang Z et al. 2020	Case series	6.63	2.07	69
Zhang JJ et al. 2020	Case series	1.88	1.45	140
Li X et al. 2020	Cross‐sectional	2.20	1.21	548
Wan S et al. 2020	Cross‐sectional	1.12	1.83	135
Xiang et al. 2020	Cross‐sectional	12.60	2.49	49
Chen et al. 2020	Retrospective cohort	3.56	1.57	150
Deng et al. 2020	Retrospective cohort	1.51	1.52	112
Feng et al. 2020	Retrospective cohort	5.25	1.25	476
Guan W et al. 2020	Retrospective cohort	2.02	1.22	1099
Huang et al. 2020	Retrospective cohort	1.18	2.38	41
Liu W et al. 2020	Retrospective cohort	2.49	2.27	78
Simone et al. 2020	Retrospective cohort	2.85	1.50	124
Wang D et al. 2020	Retrospective cohort	4.96	1.51	138
Wu C et al. 2020	Retrospective cohort	2.35	1.43	201
Zhang G et al. 2020	Retrospective cohort	4.37	1.40	221

*Note:* Source of the data de Almeida‐Pititto et al. 2020.

**FIGURE 1:**
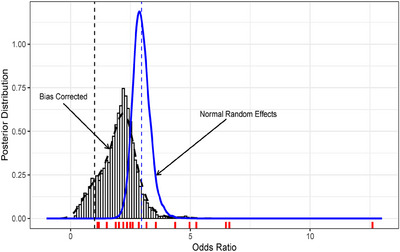
Results of the meta‐analysis comparing a random effects (RE) model (solid line) and the bias‐corrected Bayesian nonparametric (BC‐BNP) model (dashed line). Posterior distributions of the pooled odds ratio (OR) of needed for mechanical assistance for patients with hypertension. From left to right, the vertical dashed lines correspond to having no effect, OR = 1 (black), and the pooled estimate of the normal random effects, OR = 2.96 (blue). The short vertical lines at the bottom correspond to the estimated ORs from the studies.

In this meta‐analysis, researchers estimated an alarming odds ratio (OR) of 2.96 with a 95% confidence interval of [2.33, 3.76], meaning that patients admitted to hospitals with hypertension were almost three times more likely to receive mechanical respiratory assistance than patients without hypertension. The solid line in Figure 1 corresponds to the posterior pooled OR by applying a noninformative Bayesian normal random effects meta‐analysis model, which confirms this result. However, the disparity of the evidence in Table 1 comes as a warning against these results.

In this paper, we present a new meta‐analysis model, called the BC‐BNP model, where BC‐BNP stands for bias‐corrected Bayesian nonparametric. The aim, or at least the hope, is that the model automatically corrects meta‐analysis results affected by internal validity bias by only using the reported effects and their standard errors. This model relaxes the parametric assumptions of the bias distribution of the model introduced by Verde (2021). The general idea behind these models is that it is possible, at least in theory, to decompose between‐study heterogeneity into two components: a component of diversity and a component of internal validity bias (Higgins, Thompson, and Spiegelhalter 2009). We can schematically present this idea as follows:

$$\begin{array}{c} \text{Heterogeneity}\equiv \text{Diversity}\;+\;\text{Bias}. \end{array}$$

The dashed curve presented in Figure 1 corresponds to the posterior distribution of the pooled OR for the Table 1 studies calculated by using the BC‐BNP model. The posterior mean is 1.98 with a 95% posterior interval of [0.52, 3.35]. The effect of hypertension remains important, but it is moderated after bias correction and is subject to greater uncertainty. This result reduces certainty about the evidence collected in Table 1 and to the results of a meta‐analysis without bias correction.

Bayesian nonparametric (BNP) inference represents a broad and highly‐active research area in Bayesian hierarchical modeling and computational statistics. A gentle introduction of BNP is given by Rosner, Laud, and Johnson (2021) and Gelman et al. (2013). These models are “nonparametric” in the sense that they assign a stochastic process (e.g., Dirichlet process [DP], Pólya tree [PT] process), with a theoretically infinite number of parameters, to the statistical model. In this way, BNP avoids the more restrictive assumption of “parametric models,” where such a model assumes that the data can be described by a few finite numbers of parameters. From a pragmatic point of view, BNP modeling allows to acknowledge uncertainty about an assumed model, where a base distribution is assumed and realizations of the process allow variation around this distribution.

In meta‐analysis, BNP models have been developed to increase the flexibility of the random effects models. Ohlssen, Sharples, and Spiegelhalter (2007) provided an excellent practical implementation of the DP and Dirichlet process mixtures (DPMs) in meta‐analysis. The authors present the stick‐and‐break representation of the DP and DPM and their implementation in BUGS (Spiegelhalter et al. 2007). They apply these methods to a meta‐analysis of OS reporting hospitals' performances based on mortality rates (death in hospital within 30 days of emergency admission for myocardial infarction) from the United Kingdom.

Burr and Doss (2005) introduced a BNP model for random effects meta‐analysis based on DPMs. The motivation of the authors was based on a meta‐analysis of case‐control studies that investigated if there was an association between the presence of a certain genetic trait and an increased risk of coronary heart disease. The available evidence was contradictory, dispersed and motivated the development of a flexible random effects distribution. Although the DPM adds flexibility to the random effects, this happens at the cost of eliminating the interpretation of a location parameter as a pooled effect across studies. As a remedy, Burr and Doss (2005) considered a conditional DPM (CDPM) model in which the random effects distribution is constrained to have the median as the pooled effect size. The R package bspmma implements the Burr and Doss model (Burr 2012).

Branscum and Hanson (2008) developed a meta‐analysis random effects model based on mixture of Pólya Trees (MPTs), which is a family of random partitions of the sample space. PTs lend themselves naturally to fixing percentiles. For example, the first partition can be located at the median of the random effects distribution. In this way, an MPT approach allows for the desired flexibility in the random effects distribution while retaining the simplicity of the normal–normal meta‐analysis model in terms of evaluating a single location parameter. Using simulation experiments with relatively large number of studies (N=100), the authors showed that the MPT approach works well when normality holds as well as when it does not because the approach anticipates the possibility of misspecification of normality. To illustrate the MPT approach, the authors presented a meta‐analysis of prospective and retrospective OS investigating the relationship between alcohol consumption and breast cancer.

Dunson, Xue, and Carin (2008) presented the matrix stick‐breaking process (MSBP) for flexible meta‐analysis. This model is suitable in multiparameters meta‐analysis when each study reports multiple regression coefficients, but not necessarily the same ones in each study.

Karabatsos, Talbott, and Walker (2015) presented a BNP model for meta‐analysis regression, which is a special case of the adaptive‐modal BNP regression (Karabatsos and Walker 2012). This model increases the flexibility of the random effects distribution and allows nonlinear relationships in the metaregression component.

Poli et al. (2023) recently developed a multivariate PT model for meta‐analysis of S studies with time‐to‐event endpoints across n possible cohorts (i.e., n<S). In this model, the independent beta priors for the splitting probabilities in the PT construction are replaced by a Gaussian process prior. The Gaussian process links study‐specific covariates (e.g., tumor type, treatment agent, and biomarker status) to the PT partition in the logistic scale. In this way, the random partition introduces correlation between any pair of studies with similar baseline characteristics. This method is motivated by an extensive meta‐analysis of S=174 phase I/II studies on assessing the effect of biomarkers on clinical outcomes in patients with solid tumors. The authors evaluate the model using a simulation study mimicking the empirical characteristics of the motivated meta‐analysis.

These BNP meta‐analysis models have increased the flexibility of random effects distributions allowing nonlinear relationships in metaregression and complex dependencies in multivariate meta‐analysis. Moreover, they have been successfully applied to combine studies of different designs. However, these models have not been specifically tailored to adjust for multiple biases in meta‐analysis.

In Verde and Ohmann (2015), we reviewed over two decades of methods and applications of combining disparate evidence and adjusting for bias in meta‐analysis. We classified statistical approaches in four main groups: The confidence profile method (CPM, Eddy, Hasselblad, and Shachter 1992), cross‐design synthesis (CDS, Droitcour, Silberman, and Chelimsky 1993), direct likelihood bias modeling, and Bayesian hierarchical modeling. Within the Bayesian parametric hierarchical models, there are approaches to combine aggregated evidence with potential risk of bias, for example, the work of Prevost, Abrams, and Jones (2000), Welton et al. (2009), and Dias et al. (2010). These approaches differ in the way that the bias component is estimated and incorporated into the meta‐analysis model.

We organize this paper as following: In Section 2, we present methodological details of the BC‐BNP model. Here, we describe a flexible model that allows inference of the population effect in a meta‐analysis subject to studies having different degrees of internal validity bias. In Section 3, we perform a simulation study to evaluate the BC‐BNP under different scenarios, and compare it with three alternative meta‐analysis models. In Section 4, we show the BC‐BNP model in action by applying this methodology to two meta‐analyses; one meta‐analysis combines studies with different designs, and the other RCTs with varying quality. We conclude the paper in Section 5 by highlighting advantages, limitations, and pointing out possible extensions of the BC‐BNP model.

## 2 BC‐BNP Meta‐Analysis Model

In this section, we present methodological details of the BC‐BNP meta‐analysis model. This model is based upon a mixture of a parametric random effects distribution, which represents the model of interest, and a BNP model for the bias component. The resulting random effects model is a combination of a model of interest and a process of bias.

The BC‐BNP model has potentially two advantages. First, the parameters of the model of interest have a simple interpretation. Second, the bias component does not have the parametric restrictions presented in Verde (2021). In this way, we expect that it is possible to isolate the model of interest and make fewer model assumptions on the bias component. In addition, the BNP model adds flexibility to the identification of biased studies by clustering their results.

### 2.1 Modeling Biased Evidence

Suppose that a meta‐analysis of N studies has reported effect estimates $y_{1},y_{2},\text{…},y_{N}$ with their corresponding standard errors ${\text{SE}}_{1},{\text{SE}}_{2},\text{…},{\text{SE}}_{N}$. In this situation, we assume that the sample size within studies, say $n_{i}$, is large enough to ignore uncertainty on the estimated standard errors ${\text{SE}}_{i}$.

The crucial step in modeling biased evidence is to recognize that the reported effect estimate $y_{i}$ is a potentially biased version of the effect that could be observed in an “ideal study.” Therefore, the likelihood contribution of $y_{i}$ corresponds to a biased study effect $\theta_{i}^{B}$ that we model with a normal likelihood:

(1) $$y_{i}|\theta_{i}^{B}∼N\left(\theta_{i}^{B},{\text{SE}}_{i}^{2}\right),\;i=1,\text{…},N.$$

### 2.2 Modeling Heterogeneity as Diversity and Bias

Following Verde (2021), we represent the biased study effect $\theta_{i}^{B}$ as the combination of “three hidden variables”: $\theta_{i}$, $\beta_{i}$, and $I_{i}$, where

(2) $$\theta_{i}^{B}=\left(1-I_{i}\right)\;\theta_{i}+I_{i}\;\left(\theta_{i}+\beta_{i}\right),$$

which is equivalent to $\theta_{i}^{B}=\theta_{i}+I_{i}\;\beta_{i}$.

The random effect $\theta_{i}$ represents the bias‐corrected study effect, $\beta_{i}$ models the amount of the internal validity bias of the study, and the indicator variable $I_{i}$ labels if study i is biased or not, that is,

$$I_{i}=\left\{\begin{array}{cc} 1, & \text{if study}\;i\text{is biased,}\\ 0, & \text{otherwise}, \end{array}\right.$$

where $\text{Pr}(I_{i}=1)$ is the probability that the study i is biased. We assume a priori that each study has the same probability to be biased and we denote the class probability for the biased studies by $\pi^{B}$. This parameter expresses the uncertainty about the proportion of biased studies in the meta‐analysis.

We model the random effect $\theta_{i}$ as

(3) $$\theta_{i}∼N\left(\mu_{\theta },\tau_{\theta }^{2}\right),$$

where the parameters $\mu_{\theta }$ and $\tau_{\theta }^{2}$ represent the mean effect and the variability due to “diversity” across bias‐corrected studies, respectively. The random effect model (3) is “the model of interest” that we aim to isolate in the meta‐analysis.

In Verde (2021), we used a parametric model for the bias effect $\beta_{i}$. The advantage of a parametric model is the simple interpretation of the parameters. However, it is not immediately clear if a bias is discrete or continuous, or which probability distribution could be appropriate for $\beta_{i}$. In this paper, we relax the distribution assumption of $\beta_{i}$ by using a BNP prior.

More generally, let $G_{\beta }$ be the probability distribution of $\beta_{i}$. We propose to handle uncertainty around the bias effect by giving a BNP prior distribution to $G_{\beta }$. In this approach, we assume that $G_{\beta }$ is random, with a prior on the space of probability distributions. Specifically, we model the BNP prior of $G_{\beta }$ with a DP, having a base distribution $N(\mu_{\beta },\tau_{\beta }^{2})$, and concentration parameter α:

(4) $$\begin{array}{ccc} \beta_{i}|G_{\beta } & ∼ & G_{\beta },\\ G_{\beta }|\mu_{\beta },\tau_{\beta }^{2},\alpha & ∼ & DP\left(N\left(\mu_{\beta },\tau_{\beta }^{2}\right),\alpha \right). \end{array}$$

The DP prior in (4) is a discrete and infinite‐dimensional parameter prior, where $\mu_{\beta }$ and $\tau_{\beta }^{2}$ represent the location and scale of the base distribution. The prior expectation of the process corresponds to the base distribution

(5) $$E\left(G_{\beta }|\mu_{\beta },\tau_{\beta }^{2}\right)=N\left(\mu_{\beta },\tau_{\beta }^{2}\right)$$

and the prior variance depends on the base distribution and α:

(6) $$var\left(G_{\beta }|\mu_{\beta },\tau_{\beta }^{2}\right)=\frac{N\left(\mu_{\beta },\tau_{\beta }^{2}\right)\left[1-N\left(\mu_{\beta },\tau_{\beta }^{2}\right)\right]}{\alpha +1}.$$

The variance of the process (6) shows that the concentration parameter α influences the proximity of the process to the base distribution. In addition, α determines the number of clusters in the process, which is elaborated later in Equation (9).

The DP prior in (4) allows to represent the random distribution function $G_{\beta }$ with the “stick‐breaking” construction (Sethuraman 1994). In this case, $G_{\beta }$ can be expressed as

(7) $$G_{\beta }=\sum_{k=1}^{\infty }w_{k}^{*}\delta_{\beta_{k}^{*}},$$

where the stochastic weights $w_{1}^{*},w_{2}^{*},\text{…}$ and the support points $\beta_{1}^{*},\beta_{2}^{*},\text{…}$ are a priori two independent sequences of random variables, with $w_{k}^{*}>0$ and $\sum_{k=1}^{\infty }w_{k}^{*}=1$, and $\delta_{\beta_{k}^{*}}$ is the Dirac delta function, which places a measure 1 on the location $\beta_{k}^{*}$. In the representation (7), the collection of $\beta_{1}^{*},\beta_{2}^{*}\text{…}$ and $w_{1}^{*},w_{2}^{*}\text{…}$ constitutes a realization of $G_{\beta }$.

The support points $\beta_{1}^{*},\beta_{2}^{*},\text{…}$ are independently sampled from the base distribution:

(8) $$\beta_{k}^{*}∼N\left(\mu_{\beta },\tau_{\beta }^{2}\right).$$

In the presence of a positive bias, where some studies exaggerate results, we expect that $\mu_{\beta }>0$ and the ordinate values $\beta_{1}^{*},\beta_{2}^{*},\text{…}$ will be sampled to the right from the model of interest (3).

The random weights $w_{k}^{*}$ are sampled with decreasing expectations as follows:

(9) $$w_{k}^{*}=q_{k}\prod_{j<k}\left(1-q_{j}\right)\;\text{with}\;q_{k}∼\text{Beta}\left(1,\alpha \right).$$

The stick‐breaking weights $w_{1}^{*},w_{2}^{*},\text{…}$, determine the amount of clustering of the process, which is driven by the concentration parameter α.

A simple way to understand the influence of the parameter α in the number of clusters is by observing that $w_{1}^{*}∼\text{Beta}\left(1,\alpha \right)$, then

$$E\left(w_{1}^{*}\right)=\frac{1}{1+\alpha }.$$

In the limit when α→0, the first weight approaches $w_{1}^{*}\to 1$, and the random distribution $G_{\beta }$ has a single support point. In this situation, $G_{\beta }$ is quite different from the base distribution $N(\mu_{\beta },\tau_{\beta }^{2})$. When α increases, no finite collection of weights dominates and each random draw of $G_{\beta }$ becomes arbitrarily close to $N(\mu_{\beta },\tau_{\beta }^{2})$, that is, the process becomes concentrated at the base distribution. In Section 2.3, we will see that the value of α can be bounded to a finite value that reflects the sample size of the meta‐analysis and the prior expected number of biased studies.

We can summarize the model construction of $\theta_{i}^{B}$ as follows:

(10) $$\begin{array}{c} \theta_{i}^{B}∼\left\{\begin{array}{cc} N(\mu_{\theta },\tau_{\theta }^{2}), & \text{with probability}\;(1-\pi^{B}),\\ N\left(\mu_{\theta },\tau_{\theta }^{2}\right)+\sum_{k=1}^{\infty }w_{k}^{*}\delta_{\beta_{k}^{*}}, & \text{with probability}\;\pi^{B}. \end{array}\right. \end{array}$$

In (10), the biased study effect $\theta_{i}^{B}$ follows a convex combination of two stochastic processes. With probability $\left(1-\pi^{B}\right)$ we obtain a constant process, where all sequences of the process are the same, and they correspond to model of interest (3). With probability $\pi^{B}$, we obtain a random process where each sequence of the process is different and each $\beta_{i}$ is draw from a common distribution that we model with a DP.

This model construction aims to separate diversity and bias from the total heterogeneity in the meta‐analysis. Although the model is a BNP model for random effects, it has the advantage that the parameters $\mu_{\theta }$ and $\tau_{\theta }$ have a familiar interpretation.

### 2.3 Specification of Priors of Hyperparameters

Prior distributions are a fundamental part of the model, in particular for meta‐analysis models intending to correct for internal validity bias. The BC‐BNP model has two sets of hyperparameters $\mu_{\theta }$, $\tau_{\theta }$ that corresponds to the model of interest (3), and $\mu_{\beta }$, $\tau_{\beta }$, $\pi^{B}$, and α that drive the process modeling the bias (4). We assume independent hyperpriors on these parameters, and we provide a default setting for the simulation experiment of Section 3 and the applications of Section 4. Although, this default setting is specific for these applications, it can be used as guideline for similar meta‐analysis. In addition, we give a simple approximation to the random probability distribution $G_{\beta }$ to make it more computationally tractable.

#### 2.3.1 Priors for the Location Parameters $\mu_{\theta }$ and $\mu_{\beta }$

For location parameter $\mu_{\theta }$, we use $\mu_{\theta }∼N\left(0,\sigma_{\mu }^{2}\right)$, and we set the default value $\sigma_{\mu }=10^{6}$ to represent a local flat prior for $\mu_{\theta }$.

For the bias hyperparameter $\mu_{\beta }$, we use a uniform distribution: $\mu_{\beta }∼\text{Uniform}\left(B_{lower},B_{upper}\right)$. In Section 3, and in Section 4, we set the default values $B_{lower}=-15$ and $B_{upper}=15$, which correspond to an unknown bias in any direction. In addition, in Section 4 we perform a sensitivity analysis by restricting the prior of $\mu_{\beta }$ to positive bias, that is, we set $\mu_{\beta }∼\text{Uniform}\left(0,15\right)$.

#### 2.3.2 Prior for the Standard Deviation Parameters $\tau_{\theta }$ and $\tau_{\beta }$

We parametrize the prior of $\tau_{\theta }$ in terms of the precision parameter $1/\tau_{\theta }^{2}$, and we use a scale gamma with scale parameter S and with degrees of freedom df as a prior: $1/\tau_{\theta }^{2}∼\text{Scale.Gamma}\left(S,df\right)$ (Huang and Wand 2013). We set the default values S=0.5 and df=1.

The scale gamma distribution on $1/\tau_{\theta }^{2}$ implicitly defines a prior on the standard deviation $\tau_{\theta }∼S\;t^{+}\left(df\right)$, where $t^{+}\left(df\right)$ represents the t‐distribution with df degrees of freedom restricted to positive values. In the limit as the degrees of freedom df increases to infinity, the distribution then becomes a half‐normal with standard deviation S. Gelman (2006) called this prior a “weakly informative prior,” and Röver et al. (2021) discussed “weakly informative priors” in the context of the Bayesian normal–normal meta‐analysis model.

We set as default values S=0.5 and df=1, which implicitly corresponds to using a half‐Cauchy prior for $\tau_{\theta }$. This prior has $P(\tau_{\theta }<0.5)=0.5$ and $P(\tau_{\theta }>4\times 0.5)=0.16$, which covers plausible values of $\tau_{\theta }$ in the log(OR) and mean difference scale.

In a similar way, we parameterize the prior of $\tau_{\beta }$ in terms of the precision parameter $1/\tau_{\beta }^{2}$, and we use a scale gamma with scale parameter S=0.5 and with degrees of freedom df=1 as default prior for $1/\tau_{\beta }^{2}$.

#### 2.3.3 Prior for the Probability of Bias Parameter $\pi^{B}$

We model the probability of bias $\pi^{B}$ with a Beta distribution $\pi^{B}∼\text{Beta}\left(a_{0},a_{1}\right)$, and we take the default values $a_{0}=0.5$ and $a_{1}=1$. This prior reflects that we expect about one third of biased studies in the meta‐analysis, that is, $E(\pi^{B})\approx 0.33$. However, this prior is asymmetric to the right with a spike at $\pi^{B}\approx 0$. Therefore, if bias adjustment is unnecessary, posterior values of $\pi^{B}$ will be concentrated close to zero.

In addition, if we have information about the proportion of low‐quality studies that might be at risk of bias, this information can be incorporated in the analysis by eliciting the values of $a_{0}$ and $a_{1}$. Suppose that after evaluating the quality of the N studies, we suspect that $\pi^{B}$ is around ${\tilde{\pi }}^{B}$ and it would be fairly surprising that it could be greater than $Q_{bias}$. Then, we calculate $a_{0}$ and $a_{1}$, such that the median of the distribution is ${\tilde{\pi }}^{B}$ and the 90% quantile is $Q_{bias}$.

#### 2.3.4 Finite Truncation of the DP and the Prior for α

Bayesian models with infinite‐dimensional parameters require special strategies for using Markov chain Monte Carlo (MCMC) techniques to approximate posterior distributions. Two popular approaches in DP modeling are: marginalization and truncation of the process. Marginalization leads to a Pólya urn schema that allows efficient MCMC algorithms. The disadvantage of marginalization is that we cannot make inference about functions of the process, for example, calculate the expected value $E(G_{\beta })$.

Truncation allows to have a finite parameter approximation for the distribution of the process. A recent overview of truncated Dirichlet process (TDP) is given by Griffin (2016), and further developments using algorithms for adaptive empirical truncation are provided by Arbel, De Blasi, and Prünster (2019) and Zhang and Dassios (2023).

A TDP approximates $G_{\beta }$ with a maximum of K components:

(11) $$\sum_{k=1}^{\infty }w_{k}^{*}\delta_{\beta_{k}^{*}}\approx \sum_{k=1}^{K}w_{k}^{*}\delta_{\beta_{k}^{*}}.$$

In practical terms, this approximation allows clustering the bias effects $\beta_{i}$ into a maximum of K clusters, where *the maximum number of clusters* K is empirically adapted on the application context.

A pragmatic way to choose K is by making the expected probability of the final component $w_{K}^{*}=1-\sum_{k=1}^{K-1}w_{k}^{*}$ to be small:

(12) $$E(w_{K}^{*})=\varepsilon .$$

After K−1 random breaks, the remaining probability has expectation

(13) $$E\left(1-\sum_{k=1}^{K-1}w_{k}^{*}\right)=E\left(\prod_{k=1}^{K-1}\left(1-q_{k}\right)\right)={\left(\frac{\alpha }{1+\alpha }\right)}^{\left(K-1\right)}.$$

From (12) and (13), we have

(14) $$K=1+\frac{\log(\varepsilon )}{\log\left(\frac{\alpha }{1+\alpha }\right)},$$

and by taking ε=0.01 we can approximate K as

(15) K≈1+5α.

On the other hand, the maximum number of clusters $K^{max}$ is related to the prior distribution of α, that we choose to be

(16) $$\alpha ∼\text{Uniform}(0.5,\alpha^{max}).$$

We take the lower bound of 0.5 to avoid very low values of $w_{k}^{*}$ that may produce numerical issues.

Therefore, by using (15), and the upper bound of the prior (16), we can approximate the maximum number of clusters by

(17) $$K^{max}=1+5\;\alpha^{max}.$$

Now, the prior expectation of $\pi_{B}$ is

$$E\left(\pi^{B}\right)=\frac{a_{0}}{a_{0}+a_{1}},$$

which gives as an expected number of biased studies in the meta‐analysis $N^{B}$:

(18) $$N^{B}=N\;\left(\frac{a_{0}}{a_{0}+a_{1}}\right).$$

In practice, the maximum number of clusters $K^{max}$ in the bias component should not exceed the expected number of biased studies $N^{B}$. Then, we have

(19) $$K^{max}=1+5\;\alpha^{max}<N\;\left(\frac{a_{0}}{a_{0}+a_{1}}\right),$$

and solving for $\alpha^{max}$ resulted that this hyperconstant should be

(20) $$\alpha^{max}<\frac{1}{5}\left(\frac{\left(N-1\right)\;a_{0}-a_{1}}{a_{0}+a_{1}}\right).$$

We follow this pragmatic approach to specify $\alpha^{max}$ and $K^{max}$ in our applications.

### 2.4 Implementation, Bayesian Computations, and Reporting Results

The models presented in this paper are implemented in the R package **jarbes** (Verde 2024). The name of the package stands for *J*ust *a* *r*ather *B*ayesian *E*vidence *s*ynthesis. This package implements a number of Bayesian meta‐analysis models within the family of hierarchical metaregression (HMR) models. The main characteristic of HMR models is the explicit modeling of the bias process in CDS and cross‐evidence synthesis, that is, when different study types, for example, RCT, OS, and different data types, aggregated data (AD) with individual participant data (IPD) are combined in a meta‐analysis. The model presented in Section 2 is an example of an HMR, where AD data from studies of different types or varying quality are combined.

The implementation of the functions in **jarbes** follows the same strategy as the package **bamdit** (Verde 2018), where each function represents a Bayesian meta‐analysis model. Once the user selects a model's function, the function automatically writes the BUGS language script needed to perform the MCMC computations. The function sends the BUGS script to JAGS (Plummer 2003), and results are sent back to R.

The JAGS software has an internal directed acyclic graph (DAG) representation of the model. The software automatically factorizes the DAG and chooses the sample algorithm for each node, the algorithm decides the sampler according to local conjugate between the parent–child distribution. After this process, a hybrid Gibbs sampler is used for MCMC simulations.

The function bcmixmeta() implements the BC‐BNP model presented in Section 4. The outcome of this function is an R object from the class “bcmixmeta,” which contains the MCMC simulations, the model parameterization, the data used in the calculations, the JAGS script, and so forth. In particular, the JAGS script created during the application of bcmixmeta() could be useful to practitioners interested in expanding the model or using other MCMC software, such as MultiBUGS, Stan, or Nimble.

Another useful functionality is a diagnostic function, which recognizes the “bcmixmeta” object and plots the posterior results of $\Delta =\mu_{\beta }-\mu_{\theta }$ against $\pi^{B}$. This diagnostic plot was developed in Verde (2021) to assess if the bias correction was necessary. If the 95% posterior interval of Δ overlaps the horizontal line at $E(2\tau_{\theta }|\text{Data})$ then bias correction is not needed.

We summarize the results of the meta‐analyses by reporting the posterior distributions of the hyperparameters: $\mu_{\theta }$, $\tau_{\theta }$, $\mu_{\beta }$, $\tau_{\beta }$, and $\pi^{B}$. At the level of the studies, we present the posterior probabilities that a study is biased, the posteriors of the bias effect $\theta_{i}^{B}$, and the bias‐corrected effect $\theta_{i}$. For each posterior distribution, we present the mean, the standard deviation, and the 95% posterior interval.

### 2.5 Coclustering Studies

One potential advantage of the BC‐BNP model is the ability to identify subgroups of studies that tend to visit the same cluster in the MCMC iterations. This model feature could help clustering similar studies and identify those that disagree with others, and learn about disparate sources of bias across studies.

Let $L_{i}$ be the cluster label of study i, where L=1 indicates the cluster of the unbiased studies and L=2,…,K+1 the different clusters in the bias component. In each MCMC iteration t, we monitor the coclustering between studies by calculating an N×N matrix $S^{t}$ of the indicator variables $S_{i,j}^{t}$, where

(21) $$S_{i,j}^{t}=\left\{\begin{array}{cc} 1 & \text{if}\;L_{i}=L_{j}\\ 0 & \text{if}\;L_{i}\neq L_{j}. \end{array}\right.$$

The variable $S_{i,j}^{t}$ indicates whether or not two studies visit the same cluster at iteration t. Therefore, $\hat{S}$, the average of the matrix of $S^{t}$ after T iterations, estimates the coclustering probability between studies.

We define the dissimilarity distance between studies as $\hat{D}=1-\hat{S}$, and we display the coclustering results using a heatmap plot generated by the R package pheatmap (Kolde 2019). The heatmap is generated by clustering columns and rows of $\hat{D}$ with the default hierarchical clustering algorithm in R (method = “complete”).

## 3 A Simulation Study

This simulation study aims to determine whether the posterior distributions of the BC‐BNP model can detect bias under simulation conditions where bias is present and can confirm the absence of bias under data simulation condition where bias is not present. The simulation study is designed to have similar characteristics to the meta‐analyses that are presented in Section 4.

We compare the performance of the BC‐BNP model with three alternative meta‐analysis models: (1) The usual normal–normal “Bayesian random effects,” which corresponds to (3) without bias correction, (2) an “Oracle Bayesian random effects,” where biased studies were manually excluded before running the previous model, and (3) the parametric BC model (Verde 2021). We run the four models with the default hyperpriors presented in Section 2.3. Further computational details and results can be found in the R code supplement in the folder “Simulations‐Section‐3” of Supporting Information of the paper.

### 3.1 Simulation Model and Different Levels of Bias

To simplify the presentation, we work in the scale of the logarithm of the odds ratio (i.e., log(OR)), which would be a typical situation in practice. For a meta‐analysis of size N, we simulate the studies' results using a normal–normal random effects model:

(22) $$\begin{array}{ccc} & & y_{i}\left|\theta_{i},{\text{SE}}_{i}∼N\left(\theta_{i},{\text{SE}}_{i}\right),\;\text{and}\;\theta_{i}\right|\mu_{\theta },\tau_{\theta }∼N\left(\mu_{\theta },\tau_{\theta }^{2}\right)\\ & & \;(i=1,2\text{…},N), \end{array}$$

where $y_{i}$ is the simulated log(OR), and $\theta_{i}$ the effect of study i. The parameters $\mu_{\theta }$ and $\tau_{\theta }$ represent the pooled log(OR) and the between‐studies standard deviation, respectively.

In model (22), ${\text{SE}}_{i}$ is the standard error of $y_{i}$, which is equivalent to $\sqrt{\sigma^{2}/n_{i}}$. The parameter σ is the within‐study standard deviation, and $n_{i}$ is the sample size of study i. Thus, by marginalization over the study effect $\theta_{i}$, the model (22) is equivalent to

(23) $$y_{i}∼N\left(\mu_{\theta },\;\tau_{\theta }^{2}+\sigma^{2}/n_{i}\right).$$

For each simulated scenario, the sample sizes $n_{i}$ are generated at random from those of the real studies presented in Section 4. For a meta‐analysis of size N=20, we sample $n_{i}$ from the data in Section 4.2, and for N=30 we sample $n_{i}$ from the data in Section 4.1.

For all scenarios, we set the pooled log(OR) at μ=1, and τ=0.5, representing “a fairly high” heterogeneity in the log(OR) scale (Spiegelhalter, Abrams, and Myles 2004, 170). The standard error of the log(OR) derived from a 2×2 table is approximately given by ${\text{SE}}_{i}=\sigma /\sqrt{n_{i}}$. We set the value of σ=4.95, which was empirically determined by calculating the median of σ from the studies, analyzed in Section 4.

Given a sample of simulated data $y_{1},\text{…},y_{N}$, we generate B biased studies by taking a random sample without replacement of B studies out of N. The selected studies are shifted by a mean bias $\mu_{\beta }$, that is, $y_{b}+\mu_{\beta }$ (b=1,⋯,B). We add uncertainty of the amount of bias by considering that a study could have a *mild*, *large*, or *extreme bias*. We introduce this feature by considering three possible values of the mean bias: $\mu_{\beta }^{M}$, $\mu_{\beta }^{L}$, and $\mu_{\beta }^{E}$, where

(24) $$\begin{array}{ccc} \mu_{\beta }^{M} & = & Q_{3}+1.50\;\text{IQR},\;\mu_{\beta }^{L}=Q_{3}+2.25\;\text{IQR},\;\text{and}\\ \mu_{\beta }^{E} & = & Q_{3}+3.0\;\text{IQR}. \end{array}$$

The quantities $Q_{3}$ and IQR correspond to the third quartile and the inter‐quartile range of (23), respectively. For a particular simulation scenario, $Q_{3}$ and IQR are calculated by taking the marginal variance of y at $var\left(y\right)=\tau^{2}+\sigma^{2}/\bar{n}$, where $\bar{n}=1/N\;\sum_{i=1}^{N}n_{i}$.

### 3.2 Scenarios

Table 2 presents the configuration of the simulated scenarios. We consider eight possible scenarios corresponding to two meta‐analysis sample sizes, N=20 and N=30, and four percentages of biased studies, 0%, 10%, 30%, and 50%. For each scenario, we include studies with mild, large, and extreme bias. For example, in the scenario with N=20 and 50% biased studies, we assigned three studies with mild bias, three studies with large bias, and four studies with extreme bias.

**TABLE 2:** Configuration of the simulated scenarios: We considered two total sample sizes for the meta‐analysis, N=20, and N=30, and four percentages of the number of biased studies, 0%, 10%, 30%, and 50%. For each percentage, we consider different distributions of biased studies: mild, large, and extreme bias.

N	% Bias	Biased studies	Mild	Large	Extreme
20	0%	0	0	0	0
	10%	2	0	1	1
	30%	6	2	2	2
	50%	10	3	3	4
30	0%	0	0	0	0
	10%	3	1	1	1
	30%	9	3	3	3
	50%	15	5	5	5

For all models, we compare the resulting posterior distributions of $\mu_{\theta }$ and $\tau_{\theta }$. In addition, for the BC‐BNP model, we present the forest plot comparing the biased $\theta^{B}$ and bias‐corrected effects θ, and the heatmap plot for coclustering.

### 3.3 Simulation Results

The results for N=20 and N=30 were fairly similar. Therefore, in this section, we present the results for N=20. The results for N=30 are summarized in the supplementary material of this section.

**FIGURE 2:**
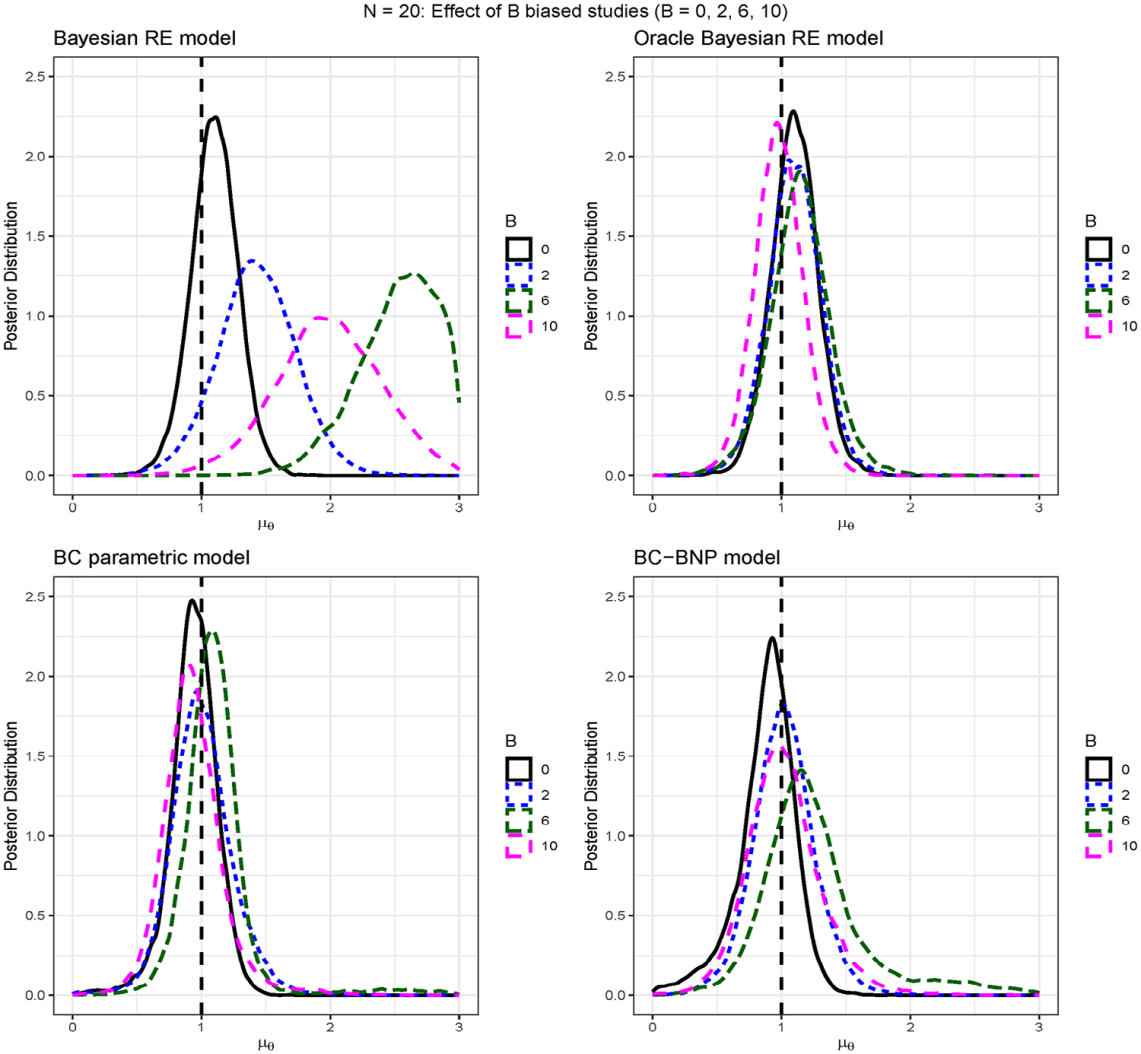
Simulation results for N=20: Comparison of four meta‐analysis models and the effect on the posterior distribution of $\mu_{\theta }$ by increasing the number of biased studies B=0,2,6,10. The models are: Bayesian random effects, Oracle Bayesian random effects (i.e., biased studies excluded), BC parametric, and BC nonparametric. The dashed vertical line corresponds to $\mu_{\theta }=1$.

Each panel of Figure 2 shows the posterior distributions of $\mu_{\theta }$ for the meta‐analysis models, and the different amounts of biased studies. In the nonbiased scenario, the Bayesian random effects model shows a posterior of $\mu_{\theta }$ centered at the true value of $\mu_{\theta }=1$. However, this model is sensitive to the biased studies, where the inclusion of two biased studies shifted the posterior to the right. As expected, the Oracle model performs correctly in all scenarios.

The lower panels of Figure 2 present the results of the parametric BC and the BC‐BNP. We see that in the nonbiased scenario, the posteriors of $\mu_{\theta }$ are correctly centered at the true value of $\mu_{\theta }=1$. In the biased scenarios, the parametric and the BC‐BNP remained robust against biased studies.

**FIGURE 3:**
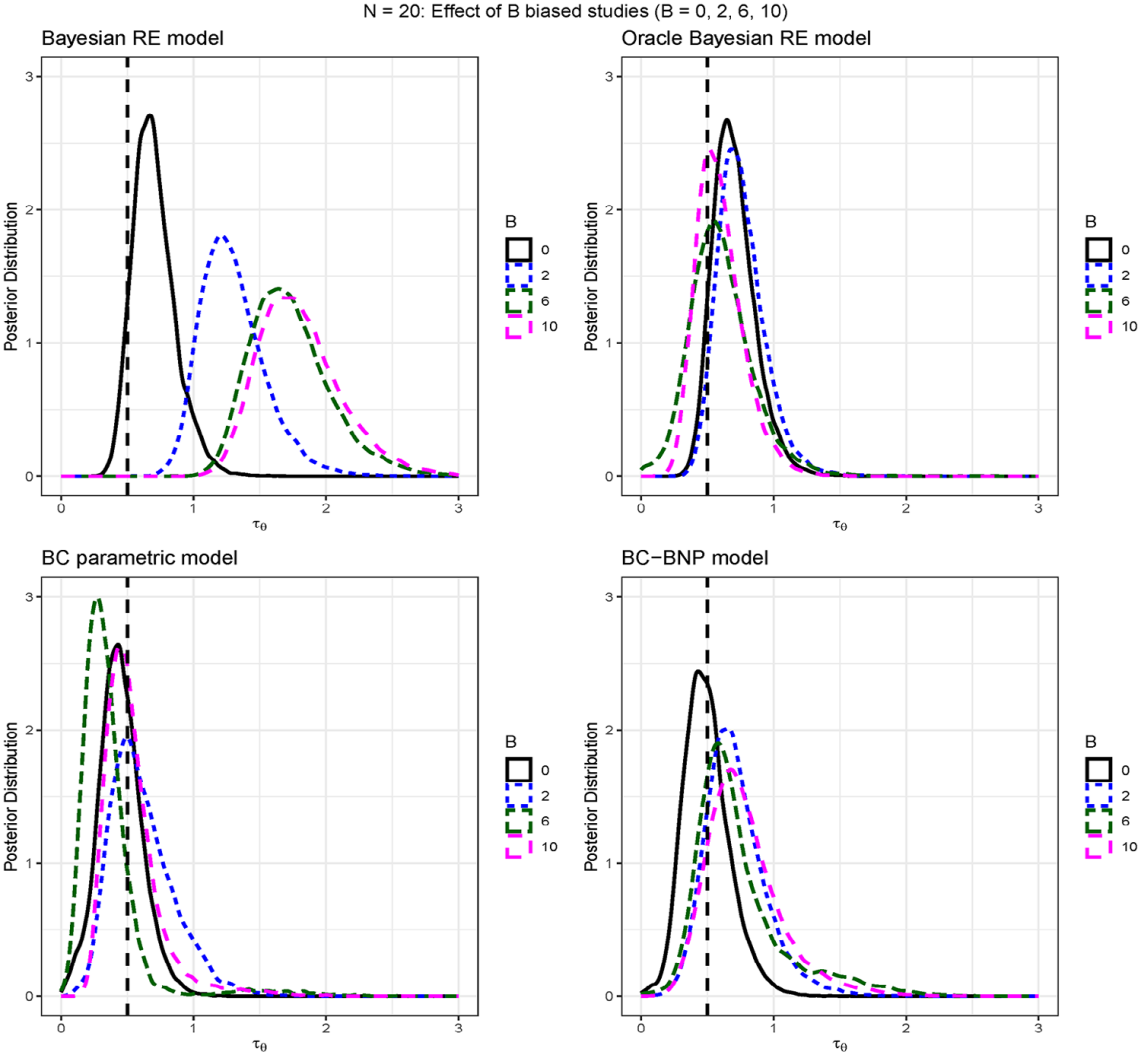
Simulation results for N=20: Comparison of four meta‐analysis models and the effect on the posterior distribution of $\tau_{\theta }$ by increasing the number of biased studies B=0,2,6,10. The models are: Bayesian random effects, Oracle Bayesian random effects (i.e., biased studies excluded), BC parametric, and BC nonparametric. The dashed vertical line corresponds to $\tau_{\theta }=0.5$.

The four panels of Figure 3 present the posterior of $\tau_{\theta }$ for each model and different biased scenarios. Similarly to the posterior of $\mu_{\theta }$, the Bayesian random effects model performed correctly in the nonbiased scenario, and was very sensitive to biased studies. The lower panels of Figure 3 show that the posteriors of the parametric BC and the BC‐BNP performed correctly in the nonbiased and the biased scenarios.

**FIGURE 4:**
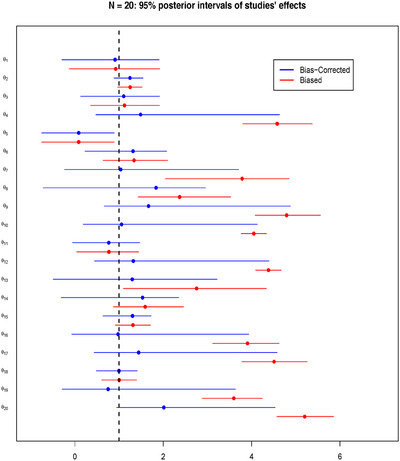
Simulation results for N=20: Forest plot comparing for each study the 95% posterior interval of the biased effect $\theta^{B}$ and the bias‐corrected effect θ. The dashed vertical line corresponds to $\mu_{\theta }=1$.

Taking the scenario of N=20 and 50% of biased studies, Figure 4 illustrates the resulting forest plot with the posterior medians and the 95% posterior intervals comparing the biased effect $\theta^{B}$, and the bias‐corrected effect θ of each study. The figure displays the amount of bias correction performed by the BC‐BNP model at the study level. For example, study number 1 has $P(I_{1}=1|\text{Data})=0.04$, and bias correction is not required. In this case, the posterior medians and the 95% intervals of $\theta_{1}^{B}$ and $\theta_{1}$ overlapped.

Different situations correspond to studies numbered 16, 19, and 20, which are generated with large, moderate, and extreme biases, respectively. These studies have the following posteriors probability of been biased: $P(I_{16}=1|\text{Data})=0.92$, $P(I_{19}=1|\text{Data})=0.90$, and $P(I_{20}=1|\text{Data})=1.00$. For these studies, Figure 4 illustrates an adaptive bias correction toward the true value $\mu_{\theta }=1$. In particular, for the extremely biased study number 20, the posteriors of $\theta_{20}^{B}$ and $\theta_{20}$ do not overlap.

**FIGURE 5:**
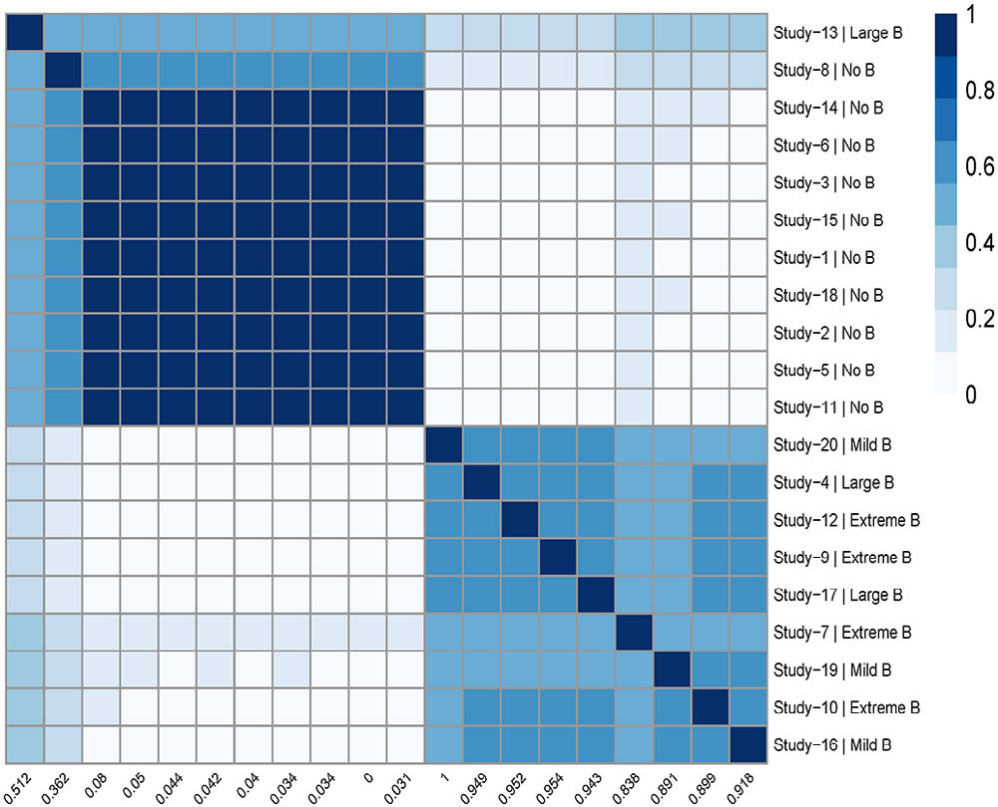
Simulation results for N=20: Heatmap of coclustering between studies sharing the same cluster. The label of the columns corresponds to the posterior probability of bias $P(I_{i}|\text{Data})$ and the label of the rows to the true bias status (No B = no bias, Mild B = mild bias, and Large B = large bias).

For the same scenario, N=20 and 50% of biased studies, the heatmap of Figure 5 illustrates the coclustering results. At the bottom of the figure, we labeled the columns with the posterior probability of bias $P(I_{i}|\text{Data})$, and on the right, we labeled the rows with the studies' numbers and the true bias status. For example, in the top left corner, Study‐13 is detected to have “large bias” with a posterior probability of bias of 0.512. The heatmap shows that the studies are grouped into two distinct clusters corresponding to the unbiased and biased studies. However, a unique feature of the BC‐BNP model is the possibility to explore coclustering within the biased studies generated by the DP. In this example, five studies (20, 9, 17, 4, and 12) and two studies (10, 16) shared the same cluster, while the remaining three biased studies (7, 13, and 19) did not cocluster with the others.

### 3.4 Conclusions of the Simulation Results

The simulation experiment of this section was neither extensive nor fully conclusive but it was challenging for any meta‐analysis model. The aim was to evaluate the BC‐BNP under different situations that represented the case studies of Section 4, and that can be found in practice. We can summarize the trends in the results of our simulation experiment as follows: (1) For the scenarios generated, the BC‐BNP model can detect bias at the study level when bias is present, and can detect nonbias when bias is absent. (2) The bias correction is adaptive according to the level of bias (no bias, mild, large, and extreme). (3) The posteriors of $\mu_{\theta }$ and $\tau_{\theta }$ are robust against biased studies. (4) It is possible to explore the coclustering of bias studies. These conclusions were the same for N=20 and N=30.

## 4 Applications

In this section, we present two real meta‐analyses. In each one, we apply the BC‐BNP model using two settings of the prior distributions. First, we apply the BC‐BNP model with informative priors, where the prior distribution of $\mu_{\beta }$ reflects the direction of bias, and the prior distribution of $\pi^{B}$ is elicited using some quality information from the studies. Second, a sensitivity analysis is performed using the default priors of the BC‐BNP model. In addition, we compare the results of the BC‐BNP model with the parametric BC model and the Bayesian random effects model.

In the Supporting Information of the paper, we present the R script to run the analyses of this section. The numerical results are based on four parallel MCMC chains, with a length of 50,000 iterations. In each chain, we discarded the first 10,000 iterations as a burn‐in period, and we apply the remaining 40,000 iterations to approximate the posterior distributions.

### 4.1 Meta‐Analysis of RCTs With Varying Quality: Stem‐Cell Treatment and Cardiovascular Disease Patients

Nowbar et al. (2014) performed a meta‐analysis consisting in N=31 RCTs of heart disease patients, where a treatment based on bone marrow stem cell was assessed for efficacy. In each trial, patients were randomized to a treatment group receiving bone marrow stem cell or a control group receiving placebo treatment. The primary endpoint was the ejection fraction. The treatment effect was defined as the difference in means of the ejection fraction between groups. The data of this meta‐analysis can be found in dataframe “stemcells” in the R package jarbes (Verde 2024).

What makes this meta‐analysis particularly interesting is the large heterogeneity between studies and the context in which these 31 RCTs were published. On the one hand, there was a high interest in assessing the efficacy of this type of treatment, but on the other hand, the varying quality of the published RCTs cast doubts on their results (Francis et al. 2013), and further investigation has found evidence of scientific misconduct (Abbott 2014).

In particular, Nowbar et al. (2014) investigated if the number of detected discrepancies in the published studies were correlated with efficacy. In this context, discrepancies were defined as two or more reported facts that cannot both be true because they are logically or mathematically incompatible. The median number of discrepancies in the meta‐analysis were 7, with a range of [0, 55], and only three studies have 0 discrepancies.

To assess the heterogeneity between studies, we applied a noninformative Bayesian normal random effects model to this meta‐analysis. The results showed a posterior pooled mean difference of $\mu_{\theta }=2.92$ with 95% posterior interval of [1.43, 4.37], which pointed out efficacy. However, the posterior between‐studies standard deviation $\sigma_{\theta }=3.44$, with a posterior 95% interval [2.40, 4.80] shows a very large heterogeneity. In addition, the predictive 95% posterior predictive interval of a future study effect $\theta^{new}$ was [−4.09, 9.93], indicating no efficacy. We can suspect that the heterogeneity was a clear combination of studies' internal validity biases and diversity, making this meta‐analysis an interesting case study for the BC‐BNP model.

We apply the BC‐BNP model presented in Section 2 to this meta‐analysis, using weakly informative priors for the model of interest. For the bias component, we elicit the hyperparameters as follows:

- We assume a positive direction of bias with a prior $\mu_{\beta }∼\text{Uniform}\left(0,15\right)$.
- For illustration, we elicit the prior of $\pi^{B}∼\text{Beta}\left(a_{0},a_{1}\right)$ by using the number of discrepancies reported by Nowbar et al. (2014), where 18 studies have five or more discrepancies. We calculate $a_{0}$ and $a_{1}$ such that the median of the $\text{Beta}(a_{0},a_{1})$ is 18/31≈0.581 and its 90th quantile is 28/31≈0.903, which result in $a_{0}=1.51$ and $a_{1}=1.17$. It is worth mentioning that in a real application, this prior should be elicited without using the data of the meta‐analysis.
- For the concentration parameter α we used a uniform distribution $\alpha ∼\text{Uniform}(0.5,\alpha^{Max})$, and by using (20) we have that $\alpha^{Max}=3.29$, and $K^{Max}=17$.
- For a prior‐to‐posterior sensitivity analysis we used the default priors, without bias direction $\mu_{\beta }∼\text{Uniform}\left(-15,15\right)$, and $\pi^{B}∼\text{Beta}\left(0.5,1\right)$, which resulted in $\alpha^{Max}=1.87$, and $K^{Max}=10$.

**FIGURE 6:**
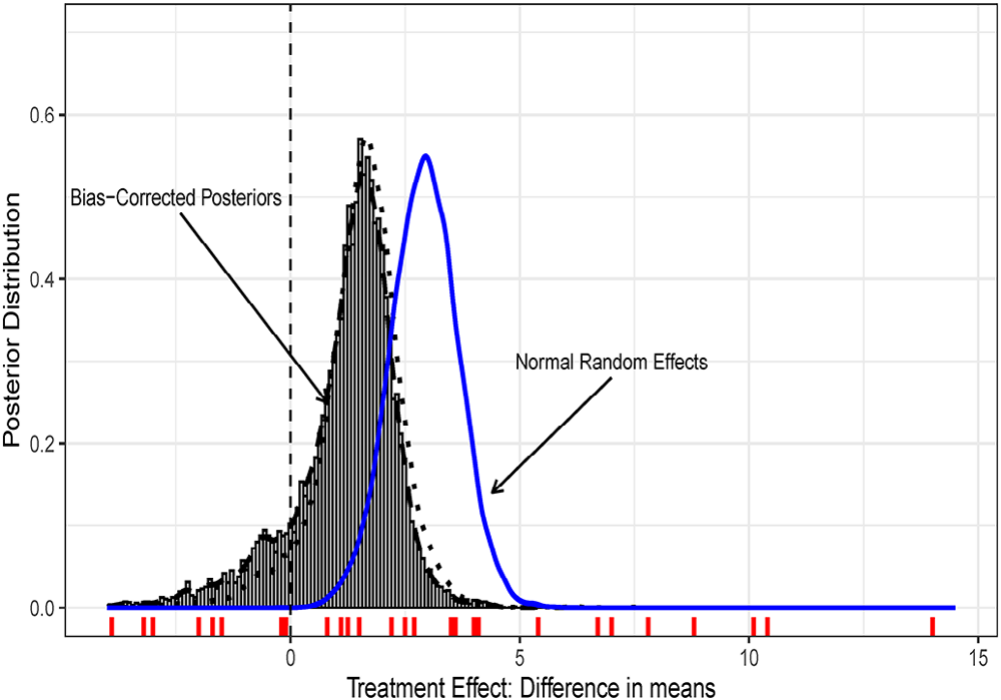
Results of the BC‐BNP model applied to efficacy of stem‐cell treatment for cardiovascular disease patients. The posterior distributions of the pooled difference in means between treatments are displayed for the BC‐BNP with informative priors (dashed line), the BC‐BNP with default priors (dotted line), and the normal–normal random effects model (solid line).

The solid line in Figure 6 presents the posterior distribution of the $\mu_{\theta }$ using a normal–normal random effects model, while the dashed line corresponds to the BC‐BNP with informative priors. The posterior mean of $\mu_{\theta }$ using the BC‐BNP supports inferring no efficacy with a posterior mean and 95% posterior interval of 1.12 [−2,29, 2.92], respectively. The dotted line presents the resulting posterior of $\mu_{\theta }$ by applying the BC‐BNP with default priors. In this case, the posterior mean was 1.51 [−0.74, 3.17]. This analysis shows that the conclusion of the lack of efficacy was not sensitive with respect to the priors of the bias component.

In addition, the posterior of $\mu_{\theta }$ using the parametric BC with default priors resulted in a posterior mean of 1.45, with a posterior 95% interval of [−1.27, 2.99], which is consistent with the BC‐BNP model.

**FIGURE 7:**
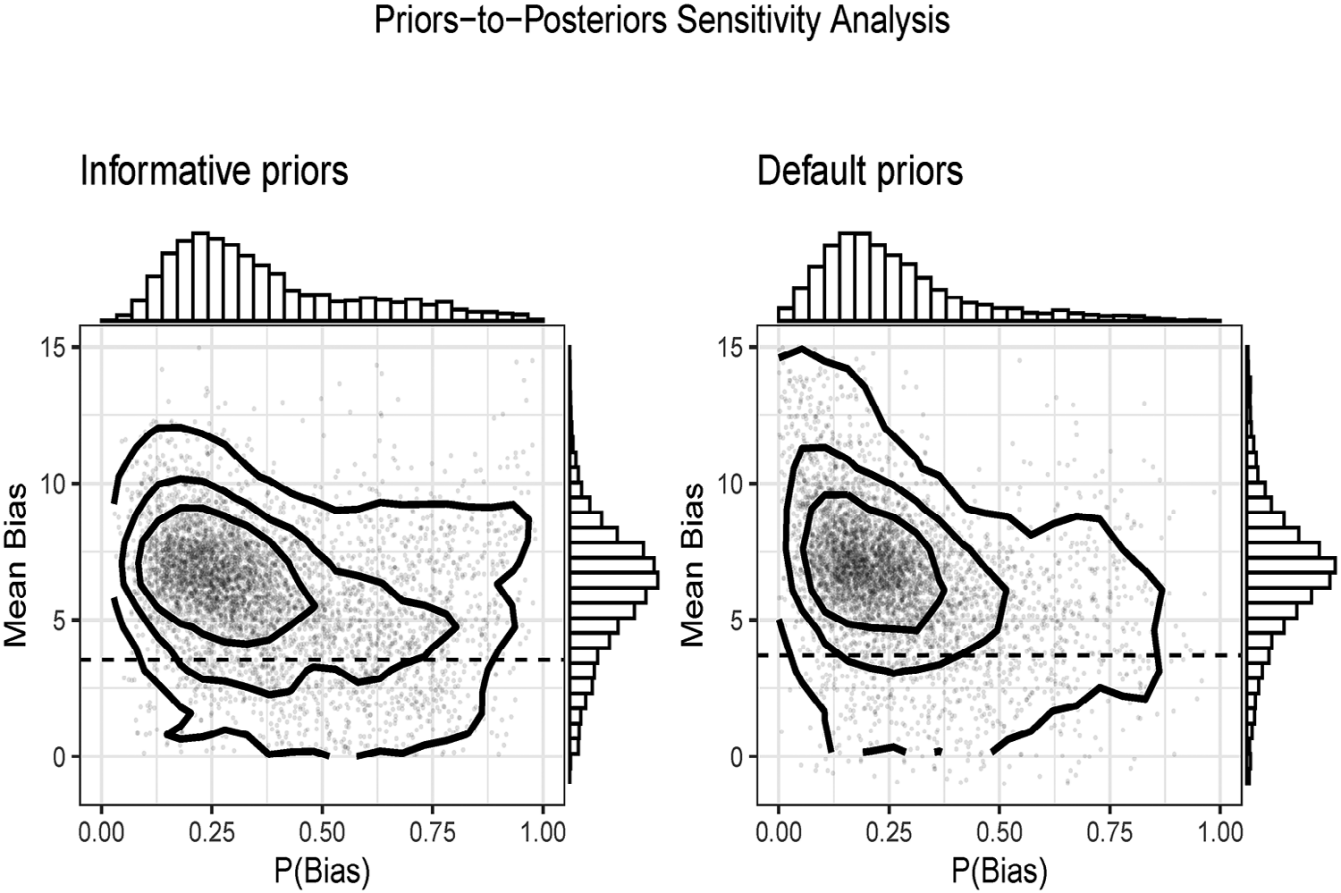
Stem‐cell treatment and cardiovascular disease patients case study. Sensitivity analysis of the priors for the bias component in the BC‐BNP model: Joint posterior distribution of the mean bias and probability of bias. The scatter plots correspond to random samples from the MCMC iterations. Left panel: Using informative prior distributions has concentrated the range of the posterior distributions. Right panel: Effect of using default priors for the bias direction and the probability of bias. The posterior of the bias remains stable, and more concentrated than the left panel.

Figure 7 shows the model diagnostic for the direction of bias. The left panel displays the joint posterior distribution between the bias correction $\Delta =\mu_{\beta }-\mu_{\theta }$ and the probability of bias $\pi^{B}$ resulted from the BC‐BNP analysis with informative priors. The right panel shows the resulting posteriors using the BC‐BNP with default priors. These diagnostic plots show that results remain stable after applying the two priors' settings.

With respect to the structure of the bias model, the posterior distributions of the number of clusters K showed lower number of clusters for default priors. For the informative priors, K has a posterior median of 6 with a 95% posterior interval [2, 10], and for default priors a median of 4 and a 95% posterior interval of [1, 8].

**FIGURE 8:**
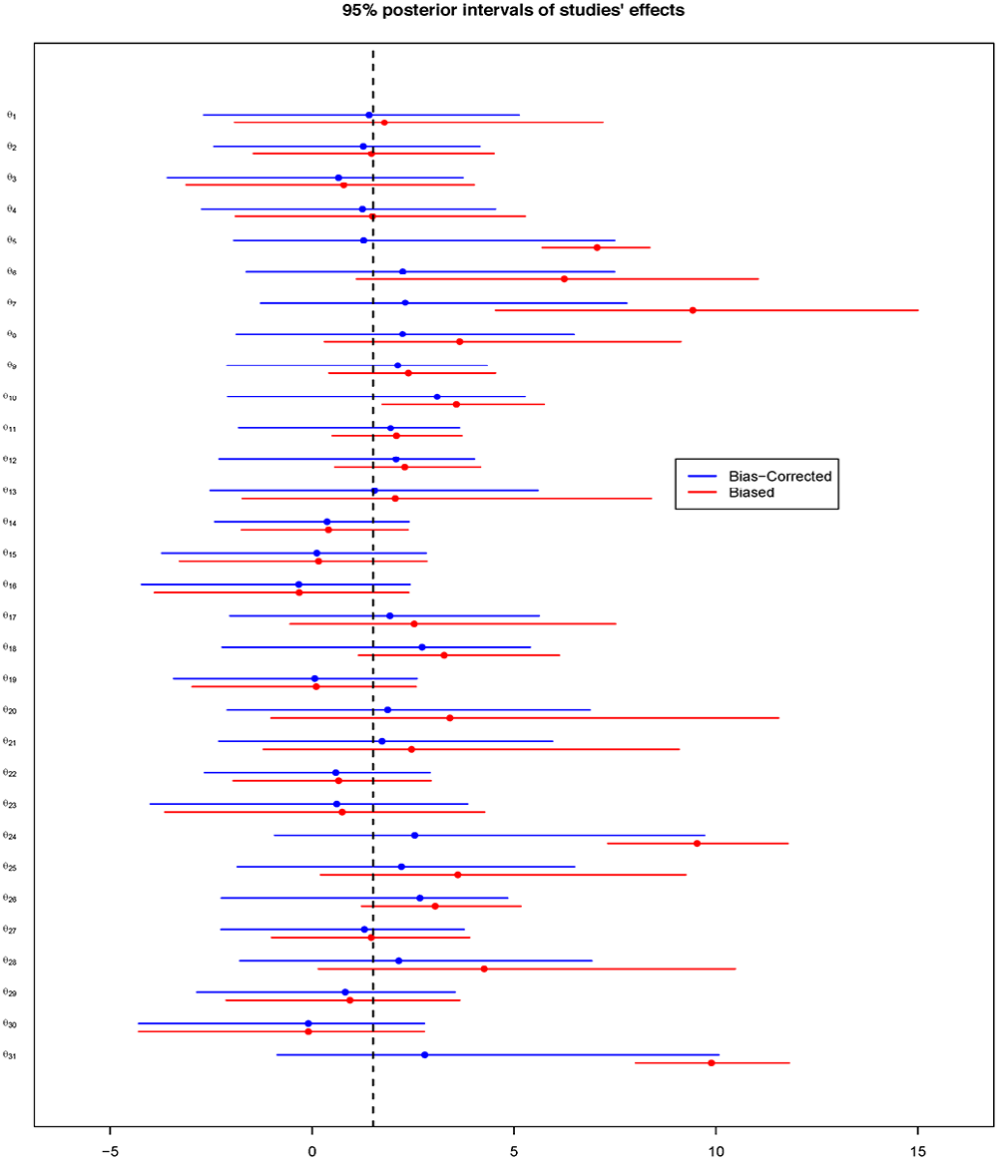
Stem‐cell treatment and cardiovascular disease patients case study. BC‐BNP model with default priors, forest plot comparing for each study the posterior distributions of the biased study effect $\theta^{B}$ (biased), and the biased‐corrected θ. The vertical dashed line corresponds to $E(\mu_{\theta }|\text{Data})=1.51$.

The forest plot in Figure 8 presents the effect of the bias correction at the study level using the BC‐BNP model with default priors. The main pattern observed in this forest plot is a shrinkage and bias correction effect for studies with larger values of $\theta^{B}$ in the direction of the posterior mean $E(\mu_{\theta }|\text{Data})=1.51$. The bias correction is adaptive; for example, studies 1, 5, 24, and 31 have posterior probabilities of being biased of 0.24, 0.88, 0.96, and 0.96, respectively.

In study 1, where bias correction is unnecessary, both posteriors overlap. For studies 5, 24, and 31, a strong bias correction is automatically performed in the direction of the posterior mean of $\mu_{\theta }=1.51$. It is worth mentioning that in the presence of bias the posteriors of θ are wider than the posterior of $\theta^{B}$ for studies 5, 24, and 31, which is “the price” we pay for bias correction at the study level.

**FIGURE 9:**
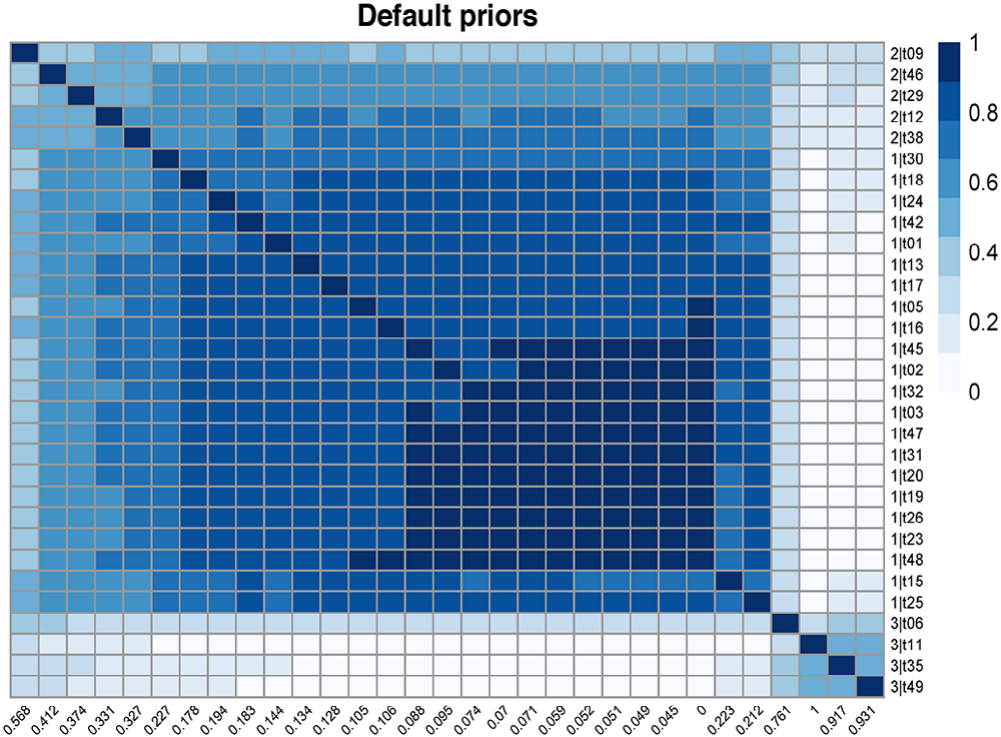
Heatmap of coclustering analysis of studies using default priors for the bias component. The labels of the columns correspond to the posterior probability of bias $P(I_{i}|\text{Data})$ and the labels of the rows correspond to the cluster label and the study number. In this example, the BC‐BNP model generated three clusters: Cluster 1 corresponds to a posterior probability of bias in the range [0–0.22], cluster 2 with a range of [0.32–0.56], and four studies coclustered at the bottom right with a range of [0.76–1].

The heatmap of Figure 9 shows a coclustering structure that resulted in three clusters. Cluster 1 corresponds to the unbiased studies with a posterior probability of bias in the range [0–0.21], cluster 2 with a range of [0.32–0.56], and four studies coclustered at the bottom right with a range of [0.76–1].

Nowbar et al. (2014) reported a lack of information about sequence allocation (randomization) in the *Risk of Bias* evaluation was linked with exaggerated treatment effects. Among the five studies in cluster 2, three did not provide clear information about sequence allocation while none of the four studies in cluster 3 had clear information. These results suggest that coclustering may be useful in linking the lack of study quality to exaggerated results.

### 4.2 Generalized Evidence Synthesis of the Relationship Between Hypertension and Severity in COVID‐19 Patients

This case study, which we briefly presented in the introduction, is a meta‐analysis of OS performed by de Almeida‐Pititto et al. (2020). The aim of the authors was to evaluate the impact of diabetes, hypertension, cardiovascular disease, and the use of angiotensin converting enzyme inhibitors/angiotensin II receptor blockers (ACEI/ARB) with the primary outcomes (1) COVID‐19 severity (including need for invasive mechanical ventilation or intensive care unit admission or O2 saturation less than90%) and (2) intrahospital mortality due to confirmed COVID‐19.

We illustrate the BC‐BNP model by analyzing the relationship between hypertension and COVID‐19 severity, where the number of included studies was N=18. From these 18 studies, five were case series, three were cross‐sectional, and 10 were retrospective cohorts. According to their study design, these studies are at the lower level of clinical evidence (Guyatt et al. 1995). Therefore, the 18 studies in this meta‐analysis are prone to internal validity bias.

This type of meta‐analysis, which combines studies of different types, is called *Generalized Evidence Synthesis*. There are two simple approaches for this type of meta‐analysis. On the one hand, we can ignore study types and combine all studies using a random effects meta‐analysis model. This is the approach that was applied by de Almeida‐Pititto et al. (2020). On the other hand, we can perform a meta‐analysis by study types, this approach has the assumption that the variability between study types is so large that we cannot gain any information by combining studies' results. We can expect that neither ignoring study types nor assuming that results are extremely different is the best way to synthesize these data.

We apply the BC‐BNP model by using weakly informative priors for the model of interest. For the bias component, we elicit the hyperparameters as follows:

- We truncate the bias prior to be positive Δ∼Uniform(0,15).
- For the probability of bias $\pi^{B}∼\text{Beta}\left(a_{0},a_{1}\right)$, we elicit the $a_{0}$ and $a_{1}$ by using the distribution of the different study types. We calculate $a_{0}$ and $a_{1}$ such most of the studies in this meta‐analysis are at risk of internal validity bias. We arbitrarily take that the median of the $\text{Beta}(a_{0},a_{1})$ is 15/18≈0.83 and its 90th quantile is 17/18≈0.944, which results in $a_{0}=8.6$ and $a_{1}=1.97$. This is an informative prior with higher probability values for large values of $\pi^{B}$.
- For the concentration parameter α, we use a uniform distribution $\alpha ∼\text{Uniform}(0.5,\alpha^{Max})$, by using (20) $\alpha^{Max}=2.73$. In this setup, the maximum number of clusters is $K^{Max}=15$.
- We perform a sensitivity analysis of the direction of bias setting the default prior $\mu_{\beta }∼\text{Uniform}\left(-15,15\right)$ and the bias information of the study types by using $\pi_{bias}∼\text{Beta}\left(0.5,1\right)$. This prior resulted in $\alpha^{Max}=1$ and the maximum number of clusters $K^{Max}=6$.

**FIGURE 10:**
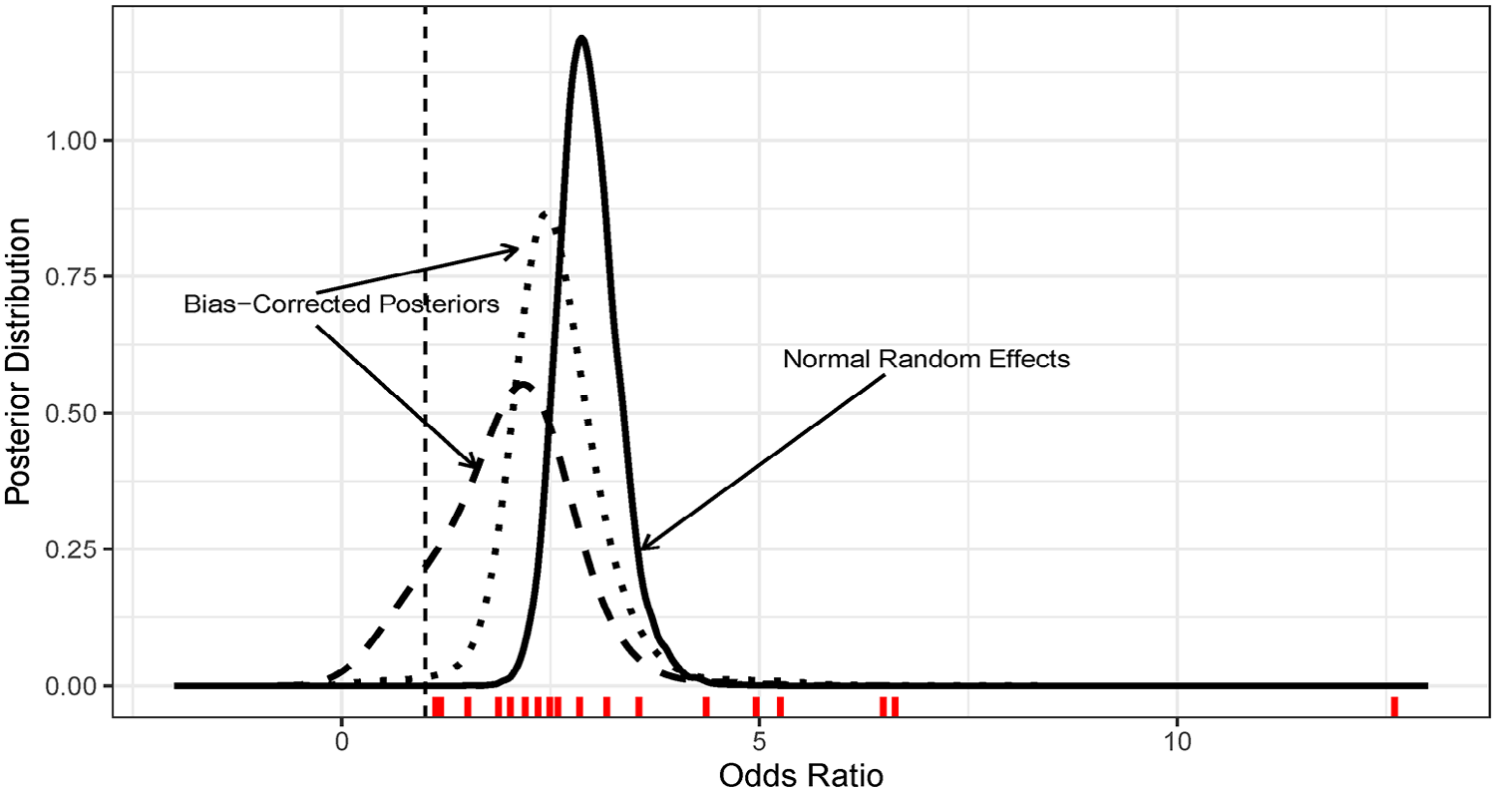
COVID‐19‐infected patients case study. Results of the BC‐BNP model applied to the relationship between hypertension and complications. The posterior distributions of the pooled OR are displayed for the BC‐BNP with informative priors (dashed line), the BC‐BNP with default priors (dotted line), and the normal–normal random effects model (solid line). The vertical dashed line corresponds to OR=1.

The solid line in Figure 10 shows the posterior distribution of the OR using a normal random effects model that shows a strong association between hypertension and complications, 2.96 [2.33, 3.76], while the dashed line corresponds to the bias‐corrected posterior using the BC‐BNP with informative priors that indicates far less support for there being an association 1.98 [0.52, 3.35]. The dotted line shows the resulting posterior when we ignore bias direction and study types, the OR has a mean posterior of 2.57 [1.60, 3.86]. The posterior of the OR using the parametric BC with default priors resulted in a posterior mean of 2.60 [1.96, 3.53], which is consistent with the default BC‐BNP model.

**FIGURE 11:**
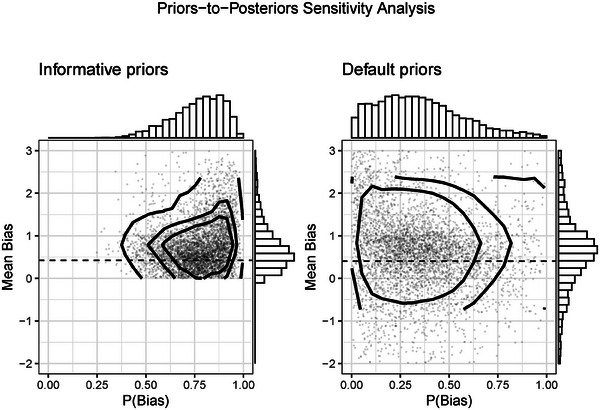
COVID‐19‐infected patients case study. Sensitivity analysis of the priors for the bias component in the BC‐BNP model: Joint posterior distribution of the mean bias and probability of bias. The scatter plots correspond to random samples from the MCMC iterations. Left panel: Using informative prior distributions has concentrated the range of the posterior distributions. Right panel: Effect of using default distributions for the bias direction and the probability of bias.

The left panel of Figure 11 shows the joint posterior distribution between the bias correction Δ and the probability of bias $\pi^{bias}$ by using informative priors. We see a high probability of bias and strong concentration of the bias correction. We can compare this result with the right panel of Figure 11, which shows the same joint posterior by using the default priors. Clearly, the use of informative priors plays an important role in this meta‐analysis.

These results show that the assessment of the study quality, and the bias direction strongly influence the conclusions from this meta‐analysis. Therefore, given the low level of clinical evidence of these N=18 studies, we could conclude that there is great uncertainty about the strength of an association.

Regarding the structure of the bias model, the posterior distributions of the number of clusters K are as following: For the informative priors, K has a median of 4 with a 95% posterior interval [1, 8], while for the default priors, K has a median of 2 with a 95% posterior interval of [1, 5].

**FIGURE 12:**
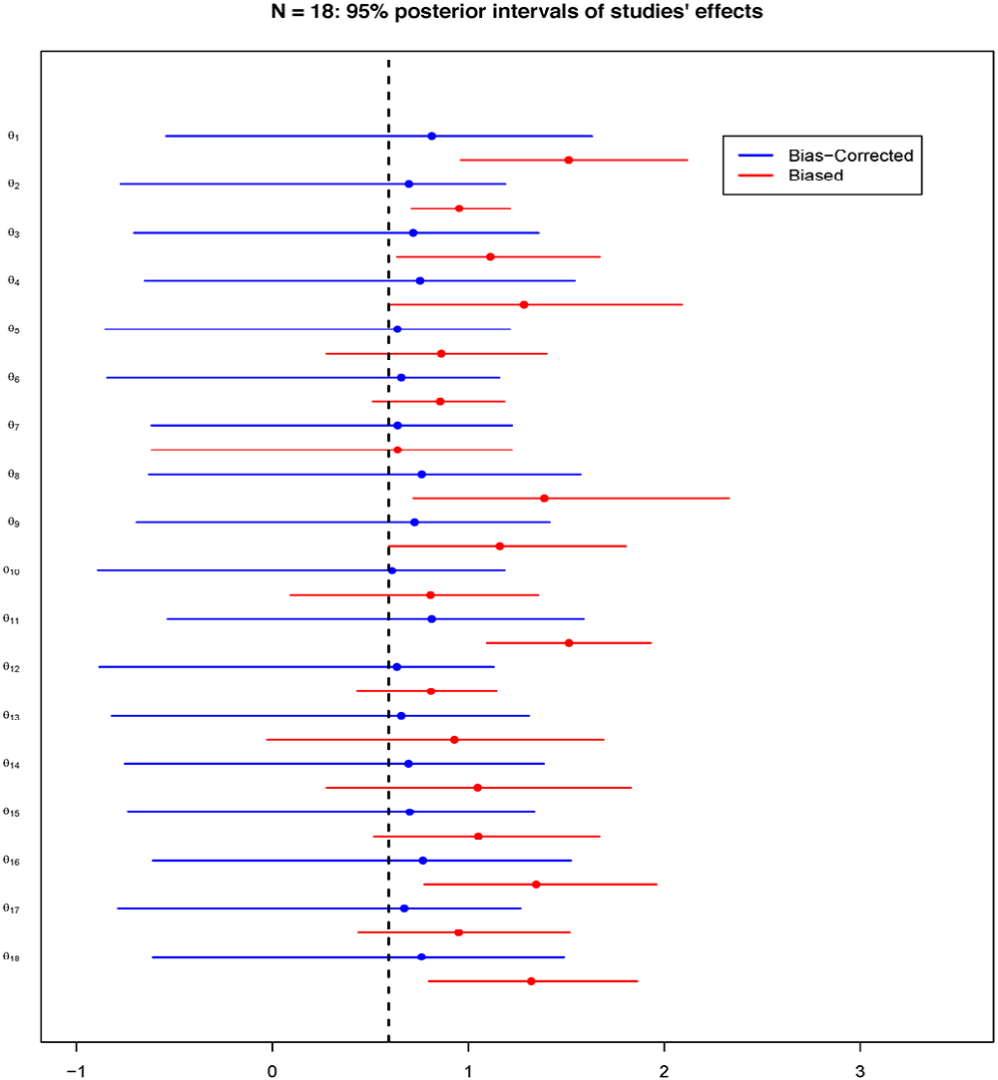
COVID‐19‐infected patients case study using BC‐BNP with informative priors. Forest plot comparing for each study the posterior distributions of the biased study effect $\theta^{B}$, and the biased‐corrected θ. The vertical dashed line corresponds to $E(\mu_{\theta }|\text{Data})=0.59$.

The forest plot of Figure 12 presents the effect of the bias correction using the BC‐BNP with informative priors. We can see that the effect of informative priors is to shift the posteriors of θ to the posterior mean $E(\mu_{\theta }|\text{Data})=0.59$.

We can compare the results for studies 1, 8, and 18. Under the BC‐BNP model with informative priors, these studies have posterior probabilities of being biased of 0.95, 1, and 0.90, respectively. For studies 1, 8, and 18, we observe a shift to the left in the posteriors of θ due to the bias correction effect. In order to interpret these results, we can refer to Table 1, where the estimated OR for studies 1 (Gua et al. 2020), 8 (Xiang et al. 2020), and 18 (Zhang et al. 2020), were 6.48, 12.60, and 4.37, respectively.

**FIGURE 13:**
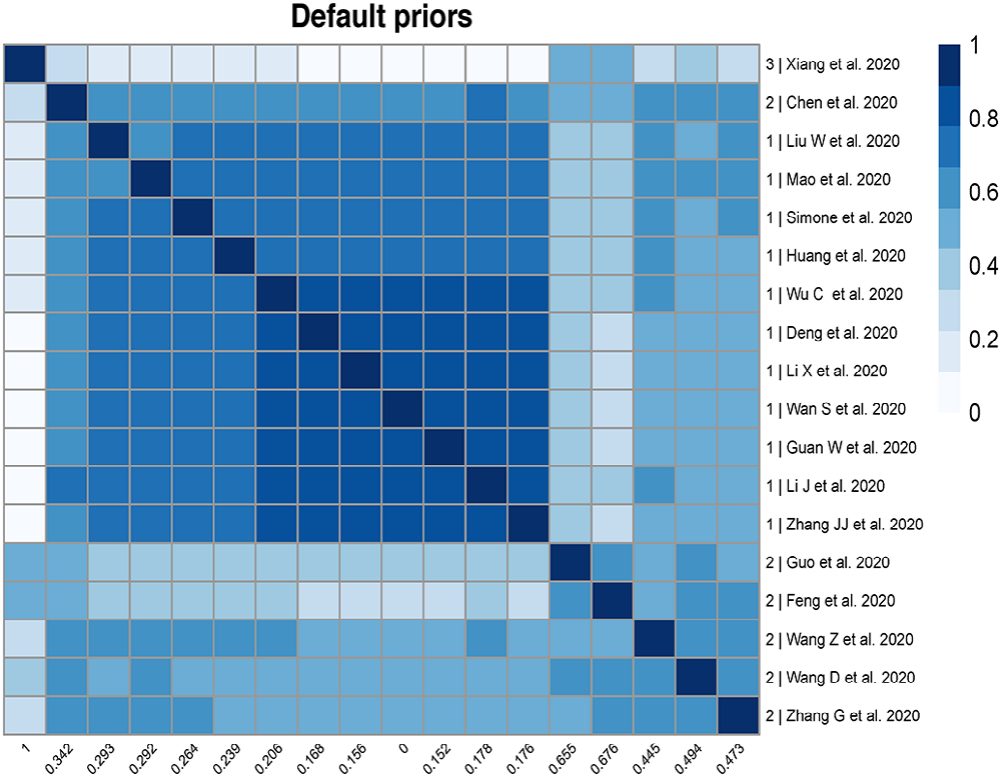
Heatmap of coclustering analysis of studies using default priors for the bias component. The labels of the columns correspond to the posterior probability of bias $P(I_{i}|\text{Data})$ and the labels of the rows correspond to the cluster label and first author of the study. In this example, the BC‐BNP model generated three clusters: The cluster 1 corresponds to studies with a probability of bias in the range [0–0.287], the cluster 2 to [0.332–0.648], and a single study, Xiang et al. (2020), is assigned to cluster 3 representing an extreme reported result. *Note:* Source of the data de Almeida‐Pititto et al. 2020.

The heatmap of Figure 13 shows that the cocluster information using the default priors agglomerated studies in three clusters. Xiang et al. (2020) is the only study in cluster 3, while the other two studies shared cluster 2. In this example, the BC‐BNP model has detected studies with overestimated ORs and low‐quality study design. It is worth mentioning that we requested a risk of bias evaluation from de Almeida‐Pititto et al. (2020), but this evaluation was neither published nor sent to us. This case study shows that the BC‐BNP model can be a useful tool to assess risk of bias by only using information about the reported effects and their standard errors.

## 5 Discussion and Conclusion

Meta‐analysis deals with combining indirect evidence in statistics, where meta‐analysis of RCTs represents the highest level of clinical evidence. However, the COVID‐19 pandemic showed that urgent informed decisions sometimes have to be based on imperfect pieces of evidence, evidence that may contain conflicting results, or be prone to bias.

In this paper, we presented the BC‐BNP model, which aims to automatically correct for internal validity bias in meta‐analysis by relaxing the parametric assumptions of the bias model introduced by Verde (2021).

We evaluated the BC‐BNP model using simulated data sets and we illustrate its application with two real case studies. Our results showed several potential advantages of the BC‐BNP in practical applications. First, the BC‐BNP model can detect bias when present and yields results similar to a simple normal–normal random effects model when bias is absent. Second, relaxing the parametric assumptions of the bias component does not affect the model of interest and provides results consistent with Verde (2021) model. Third, having a BNP component of bias may offer a better insight into the nature of biases by clustering studies with similar biases.

The setup of the default hyperpriors we provided represent a neutral elicitation on the direction of bias and a relative optimistic assumption about the number of studies at risk of bias. However, external validity bias represents unobserved external factors that cannot be directly estimated from studies' results. Therefore, we recommend performing a sensitivity analysis on the direction of bias and on the number of studies at risk of bias.

The examples in Section 4 demonstrated the extent to which bias correction is possible and whether this correction remains stable after a sensitivity analysis. For the stem‐cell example, the results were consistent across different hyperpriors, indicating that there is sufficient observed information to assert the presence of bias. However, in the COVID example, the results differed between the informative and default priors. In this case, the BC‐BNP model indicates that combining results at face value maybe misleading, and a prior elicitation is crucial to arrive at useful conclusions.

The BNP component of the BC‐BNP model demonstrates its potential as an exploratory tool for clustering studies with varying levels of bias. We can expect this feature to be even more useful in more complex meta‐analysis models (e.g., metaregression, hierarchical meta‐analysis of subgroups), but it remains a topic for further research.

There are several potential extensions of the BC‐BNP model that we did not cover in this paper. One important topic is using exact likelihood contributions in the meta‐analysis model. In this paper, we simplify this point by assuming normal likelihood approximations, but we know that different endpoint types may influence meta‐analysis results. An important type of bias in meta‐analysis is the publication bias, where small‐sample studies may overreport significant results. A possible extension of the BC‐BNP could involve modifying the base distribution of the DP to include the sample sizes of the studies and correct for publication bias. Other important extensions include a hierarchical BC‐BNP model combining several studies subgroups within each publication, and external validity bias correction by combining AD and IPD in meta‐analysis (Verde 2017). Furthermore, a methodological comparison of BNP models in meta‐analysis and the extent to which these models indirectly correct for bias remains an open research topic.

This work leads to two important conclusions. First, extending the flexibility of the random effects distribution is a valuable approach. One practical aspect, demonstrated empirically in this paper, is the possibility to obtain results that are robust against biased studies. Second, ignoring internal validity bias in meta‐analysis constitutes a form of model misspecification that can lead to misleading conclusions. The approach presented in this paper offers a promising solution to this important problem in meta‐analysis.

## Conflicts of Interest

The authors declare no conflicts of interest.

### Open Research Badges

This article has earned an Open Data badge for making publicly available the digitally‐shareable data necessary to reproduce the reported results. The data is available in the Supporting Information section.

This article has earned an open data badge “**Reproducible Research**” for making publicly available the code necessary to reproduce the reported results. The results reported in this article could fully be reproduced.

## Supporting information

Supporting Information

## Data Availability

The data that support the findings of this study are openly available in the R package jarbes at https://cran.r‐project.org/web/packages/jarbes/index.html.
