# Supplementary material for: A Bias‐Corrected Bayesian Nonparametric Model for Combining Studies With Varying Quality in Meta‐Analysis
Source: Biom J. 2025 Feb 7;67(1):e70034. doi: 10.1002/bimj.70034 (PMC11803498; doi:10.1002/bimj.70034)
Supplement: Supplementary file 1 — Supporting Information [file BIMJ-67-e70034-s001.zip › Data_and_Software/Case-studies-Section-4/BC-BNP-Section-4-January-2025.pdf]

A Bias-Corrected Bayesian Nonparametric Model for Combining Studies with Varying Quality in Meta-Analysis

Supplementary Material: Sections 1, 2, and 4

Pablo E. Verde

and

Gary L. Rosner

08 Januar 2025

Contents

1 Statistical computations **2**
1.1 Covid example 2
1.1.1 Data setup for hypertension and severity 2
1.1.2 Bayesian Normal Random Effects 2
1.1.3 BC-BNP informative priors 2
1.1.3.1 Determination of the priors 2
1.1.4 BC-BNP defualt priors 4
1.1.4.1 Determination of priors 4
1.1.5 BC parametric model with default priors 5
1.2 Stem cells example 5
1.2.1 Data setup 5
1.2.2 Bayesian Normal Random Effects 5
1.2.3 BC-BNP: informative priors 5
1.2.3.1 Determination of the priors 5
1.3 BC-BNP: default priors 7
1.4 BC parametric model with default priors 8

2 Results of the COVID-19 example **8**
2.0.1 Table 1 for the introduction 8
2.0.2 Text for the introduction 9
2.0.3 Figure 1: Introduction 10
2.1 Text Section 2.1 10
2.2 Results for Section 4.2 10
2.2.1 Figure 10 12
2.2.2 Figure 11: diagnostic plots 12
2.2.3 Figure 12: Forest plot informative priors comparing θ^B and θ 14
2.2.4 Individual cases informative priors 15
2.2.5 Figure 13: Co-clustering analysis with default priors 15

3 Results of the stem cells example **17**
3.0.1 Results of Section 4.1 17
3.0.2 Figure 6 of Section 4.1 18

3.0.3 Figure 7 of Section 4.1 19
3.0.4 Figure 8 of Section 4.1: Forest plot default priors comparing θ^B and θ 20
3.0.5 Four cases informative priors 20
3.0.6 Figure 9 of Section 4.1 21
3.0.7 Interpretation about co-clustering based on the Risk of Bias evaluation 22

1 Statistical computations

1.1 Covid example

1.1.1 Data setup for hypertension and severity

```
data("covid19")
hyper.severity = covid19 %>%
                 filter(
                  risk.factor=="hypertension"&
                  endpoint=="severity") %>%
                 select(author, design,TE,seTE,N) %>% arrange(design)
dim(hyper.severity)
```

```
## [1] 18  5
```

```
table(hyper.severity$design)
```

```
##
##          Case Series      Cross Sectional Retrospective Cohort
##                    5                    3                   10
```

1.1.2 Bayesian Normal Random Effects

1.1.3 BC-BNP informative priors

1.1.3.1 Determination of the priors

Informative prior distribution for the probability of bias.

```
#  Number of papers with high risk of bias

table(hyper.severity$design)
```

```
##
##          Case Series      Cross Sectional Retrospective Cohort
##                    5                    3                   10
```

Text for section 4.2

```
# Probability to be biased
# Number of studies = 18
# most of the studies are biased ...

# median 15/18
# 90th percentile 17/18

quantile1=list(p = 0.5, x = 15/18)
quantile2=list(p = 0.9, x = 17/18)

#beta.select function from Learbayes:
beta.par = beta.select(quantile1, quantile2)
alpha.bias = beta.par[1]
```

```
 beta.bias = beta.par[2]

a.0 = alpha.bias
a.1 = beta.bias

a.0
```

```
## [1] 8.6
```

```
a.1
```

```
## [1] 1.97
```

Hyper-parameters for the prior of *alpha* and the maximum number of clusters K:

```
N = 18 #dim(hyper.severity)[1]
alpha.max = 1/5 * ( (N-1)*a.0 - a.1)/(a.0 + a.1)
alpha.max
```

```
## [1] 2.73
```

```
# K.max

K.max = 1 + 5*alpha.max
K.max = round(K.max)
K.max
```

```
## [1] 15
```

```
set.seed(20233)

bcmix.1.covid = bcmixmeta(hyper.severity,
              mean.mu.0=0, sd.mu.0=10^6,
              B.lower = 0,                  # Positive direction of bias
              B.upper = 15,                 #
              alpha.0 = 0.5,                # Very important
              alpha.1 = alpha.max,          # Important
              a.0 = alpha.bias,
              a.1 = beta.bias,
              K = K.max,
              df.scale.between = 1,
              scale.sigma.between = 0.5,
              scale.sigma.beta = 1,
              df.scale.beta = 0.5,
              nr.chains = 4,
              nr.iterations = 50000,
              nr.adapt = 1000,
              nr.burnin = 10000,
              nr.thin = 1
             )
```

```
## Compiling model graph
##    Resolving undeclared variables
##    Allocating nodes
## Graph information:
##    Observed stochastic nodes: 20
##    Unobserved stochastic nodes: 88
##    Total graph size: 1376
```

```
##
## Initializing model
```

1.1.4 BC-BNP defualt priors

1.1.4.1 Determination of priors

Hyper-constants for the probability of bias.

```
a.0 = 0.5
a.1 = 1
```

Hyper-parameters for the prior of *alpha* and the maximum number of clusters K:

```
N = dim(hyper.severity)[1]
alpha.max = 1/5 * ( (N-1)*a.0 - a.1)/(a.0 + a.1)
alpha.max
```

```
## [1] 1
```

```
# K.max

K.max = 1 + 5*alpha.max
K.max = round(K.max)
K.max
```

```
## [1] 6
```

```
set.seed(20233)

bcmix.2.covid = bcmixmeta(hyper.severity,
              mean.mu.0 = 0, sd.mu.0 = 10^6,
              B.lower = -15,                 # No bias direction
              B.upper =  15,                 #
              alpha.0 = 0.5,                 # Very important
              alpha.1 = alpha.max,           # Important
              a.0 = a.0,
              a.1 = a.1,
              K = K.max,
              df.scale.between = 1,
              scale.sigma.between = 0.5,
              scale.sigma.beta = 1,
              df.scale.beta = 0.5,
              nr.chains = 4,
              nr.iterations = 50000,
              nr.adapt = 1000,
              nr.burnin = 10000,
              nr.thin = 1
             )
```

```
## Compiling model graph
##    Resolving undeclared variables
##    Allocating nodes
## Graph information:
##    Observed stochastic nodes: 20
##    Unobserved stochastic nodes: 70
##    Total graph size: 1106
##
## Initializing model
```

1.1.5 BC parametric model with default priors

```
bc.covid = bcmeta(hyper.severity,
              B.lower = -15,                # No bias direction
              B.upper = 15,                 # Important
              a.0 = 0.5,
              a.1 = 1,
              df.scale.between = 1,
              scale.sigma.between = 0.5,
              nr.chains = 4,
              nr.iterations = 50000,
              nr.adapt = 1000,
              nr.burnin = 10000,
              nr.thin = 4
              )
```

```
## Compiling model graph
##    Resolving undeclared variables
##    Allocating nodes
## Graph information:
##    Observed stochastic nodes: 20
##    Unobserved stochastic nodes: 94
##    Total graph size: 274
##
## Initializing model
```

1.2 Stem cells example

1.2.1 Data setup

```
data("stemcells")
stemcells$TE = stemcells$effect.size
stemcells$seTE = stemcells$se.effect
```

1.2.2 Bayesian Normal Random Effects

```
m.0.stemcells = bmeta(stemcells, mean.mu = 0, sd.mu = 10^6,
                  scale.sigma.between = 0.5, df.scale.between = 1,
                  nr.iterations = 50000,
                  nr.thin = 4,
                  nr.burnin = 10000)
```

1.2.3 BC-BNP: informative priors

1.2.3.1 Determination of the priors

Prior for the probability of bias.

```
#  Number of papers with more than 5 discrepancies ...
 table(stemcells$n.discrep>5)
```

```
##
## FALSE  TRUE
##    13    18
```

```
 table(stemcells$n.discrep)
```

```
##
```

```
##  0  1  2  3  4  6  7  8  9 11 13 15 16 17 18 19 21 27 55
##  3  1  5  2  2  2  3  1  1  2  1  1  1  1  1  1  1  1  1
```

```
# The median of the prior is: (Number of with discrepancies >5)  / Total = 18/31
# and the 90th percentile of the prior is: (Number of with discrepancies >5)  / Total = 18/31
#
# The idea is to limit the number of biased studies up to the number of discrepancies >5
# and open the possibility to include unbiased studies, which are OS.

#
# quantile1=list(p = 0.5, x = 18/31) # 18 with more than 5 discrepancies
# quantile2=list(p = 0.9, x = 28/31) # Only 3 without discrepancies
#
quantile1=list(p = 0.5, x = 18/31) # 18 with more than 5 discrepancies
quantile2=list(p = 0.9, x = 28/31) # Only 3 without discrepancies

beta.par = beta.select(quantile1, quantile2)
alpha.bias = beta.par[1]
beta.bias = beta.par[2]

a.0 = alpha.bias
a.1 = beta.bias
a.0
```

```
## [1] 1.51
```

```
a.1
```

```
## [1] 1.17
```

Hyper-parameters for the prior of $alpha$ and the maximum number of clusters K:

```
N = dim(stemcells)[1]
alpha.max = 1/5 * ( (N-1)*a.0 - a.1)/(a.0 + a.1)
alpha.max
```

```
## [1] 3.29
```

```
# K.max

K.max = 1 + 5*alpha.max
K.max = round(K.max)
K.max
```

```
## [1] 17
```

```
set.seed(20233)

bcmix.1.stemcell = bcmixmeta(stemcells,
              mean.mu.0=0, sd.mu.0=10^6,
              B.lower = 0,
              B.upper = 15,                # Important
              alpha.0 = 0.5,
              alpha.1 = alpha.max,          # Important
              a.0 = alpha.bias,
              a.1 = beta.bias,
              K = K.max,
              df.scale.between = 1,
```

```
              scale.sigma.between = 0.5,
              nr.chains = 4,
              nr.iterations = 50000,
              nr.adapt = 1000,
              nr.burnin = 10000,
              nr.thin = 4
              )
```

```
## Compiling model graph
##    Resolving undeclared variables
##    Allocating nodes
## Graph information:
##    Observed stochastic nodes: 33
##    Unobserved stochastic nodes: 131
##    Total graph size: 3110
##
## Initializing model
```

1.3 BC-BNP: default priors

Prior for the probability of bias.

```
# Beta(0.5, 1)
a.0 = 0.5
a.1 = 1
```

Hyper-parameters for the prior of *alpha* and the maximum number of clusters K:

```
# alpha.max
N = dim(stemcells)[1]
alpha.max = 1/5 * ( (N-1)*a.0 - a.1)/(a.0 + a.1)
alpha.max
```

```
## [1] 1.87
```

```
# K.max
K.max = 1 + 5*alpha.max
K.max = round(K.max)
K.max
```

```
## [1] 10
```

```
set.seed(20233)

bcmix.2.stemcell = bcmixmeta(stemcells,
              mean.mu.0=0, sd.mu.0=100,
              B.lower = -15,
              B.upper = 15,                 # Important
              alpha.0 = 0.5,
              alpha.1 = alpha.max,          # Important
              a.0 = a.0,
              a.1 = a.1,
              K = K.max,
              df.scale.between = 1,
              scale.sigma.between = 0.5,
              nr.chains = 4,
              nr.iterations = 50000,
```

```
               nr.adapt = 1000,
               nr.burnin = 10000,
               nr.thin = 4
               )
```

```
## Compiling model graph
##    Resolving undeclared variables
##    Allocating nodes
## Graph information:
##    Observed stochastic nodes: 33
##    Unobserved stochastic nodes: 117
##    Total graph size: 2809
##
## Initializing model
```

1.4 BC parametric model with default priors

```
bc.stemcell = bcmeta(stemcells,
               B.lower = -15,
               B.upper = 15,                 # Important
               a.0 = 0.5,
               a.1 = 1,
               df.scale.between = 1,
               scale.sigma.between = 0.5,
               nr.chains = 4,
               nr.iterations = 50000,
               nr.adapt = 1000,
               nr.burnin = 10000,
               nr.thin = 4)
```

```
## Compiling model graph
##    Resolving undeclared variables
##    Allocating nodes
## Graph information:
##    Observed stochastic nodes: 33
##    Unobserved stochastic nodes: 159
##    Total graph size: 453
##
## Initializing model
```

2 Results of the COVID-19 example

2.0.1 Table 1 for the introduction

```
hyper.severity$OR = exp(hyper.severity$TE)
hyper.severity$seOR = exp(hyper.severity$seTE)

table.1 <- xtable(hyper.severity[, c("author", "design", "OR", "seOR", "N" )], label = "table-1", capti

print(table.1)
```

```
## % latex table generated in R 4.4.2 by xtable 1.8-4 package
## % Wed Jan  8 11:09:36 2025
## \begin{table}[ht]
```

```
## \centering
## \begin{tabular}{rllrrr}
##   \hline
##  & author & design & OR & seOR & N \\
##   \hline
## 1 & Guo et al. 2020 & Case Series & 6.48 & 1.43 & 187 \\
##   2 & Li J et al. 2020 & Case Series & 2.59 & 1.14 & 1178 \\
##   3 & Mao et al. 2020 & Case Series & 3.17 & 1.39 & 214 \\
##   4 & Wang Z et al. 2020 & Case Series & 6.63 & 2.07 &  69 \\
##   5 & Zhang JJ et al. 2020 & Case Series & 1.88 & 1.45 & 140 \\
##   6 & Li X et al. 2020 & Cross Sectional & 2.20 & 1.21 & 548 \\
##   7 & Wan S et al. 2020 & Cross Sectional & 1.12 & 1.83 & 135 \\
##   8 & Xiang et al. 2020 & Cross Sectional & 12.60 & 2.49 &  49 \\
##   9 & Chen et al. 2020 & Retrospective Cohort & 3.56 & 1.57 & 150 \\
##   10 & Deng et al. 2020 & Retrospective Cohort & 1.51 & 1.52 & 112 \\
##   11 & Feng et al. 2020 & Retrospective Cohort & 5.25 & 1.25 & 476 \\
##   12 & Guan W et al. 2020 & Retrospective Cohort & 2.02 & 1.22 & 1099 \\
##   13 & Huang et al. 2020 & Retrospective Cohort & 1.18 & 2.38 &  41 \\
##   14 & Liu W et al. 2020 & Retrospective Cohort & 2.49 & 2.27 &  78 \\
##   15 & Simone et al. 2020 & Retrospective Cohort & 2.85 & 1.50 & 124 \\
##   16 & Wang D et al. 2020 & Retrospective Cohort & 4.96 & 1.51 & 138 \\
##   17 & Wu C  et al. 2020 & Retrospective Cohort & 2.35 & 1.43 & 201 \\
##   18 & Zhang G et al. 2020 & Retrospective Cohort & 4.37 & 1.40 & 221 \\
##    \hline
## \end{tabular}
## \caption{Example of meta-analysis}
## \label{table-1}
## \end{table}
```

2.0.2 Text for the introduction

Bayesian random effects:

```
attach.jags(m.0.covid, overwrite = TRUE)

mu.re = exp(mu)

round(mean(mu.re), 2)
```

```
## [1] 2.96
```

```
round(quantile(mu.re, prob = c(0.025, 0.975)), 2)
```

```
##  2.5% 97.5%
##  2.33  3.76
```

BC-BNP with informative priors:

```
# Text introduction: Informative priors ...
attach.jags(bcmix.1.covid, overwrite = TRUE)

OR = exp(mu.0)

round(mean(OR), 2)
```

```
## [1] 1.98
```

```r
round(quantile(OR, prob = c(0.025,0.5, 0.975)), 2)
```

```
##  2.5%   50% 97.5%
##  0.52  2.06  3.35
```

2.0.3 Figure 1: Introduction

Comparison of the posterior distribution of the pooled treatment effect mu with the Normal and BC-BNP random effects.

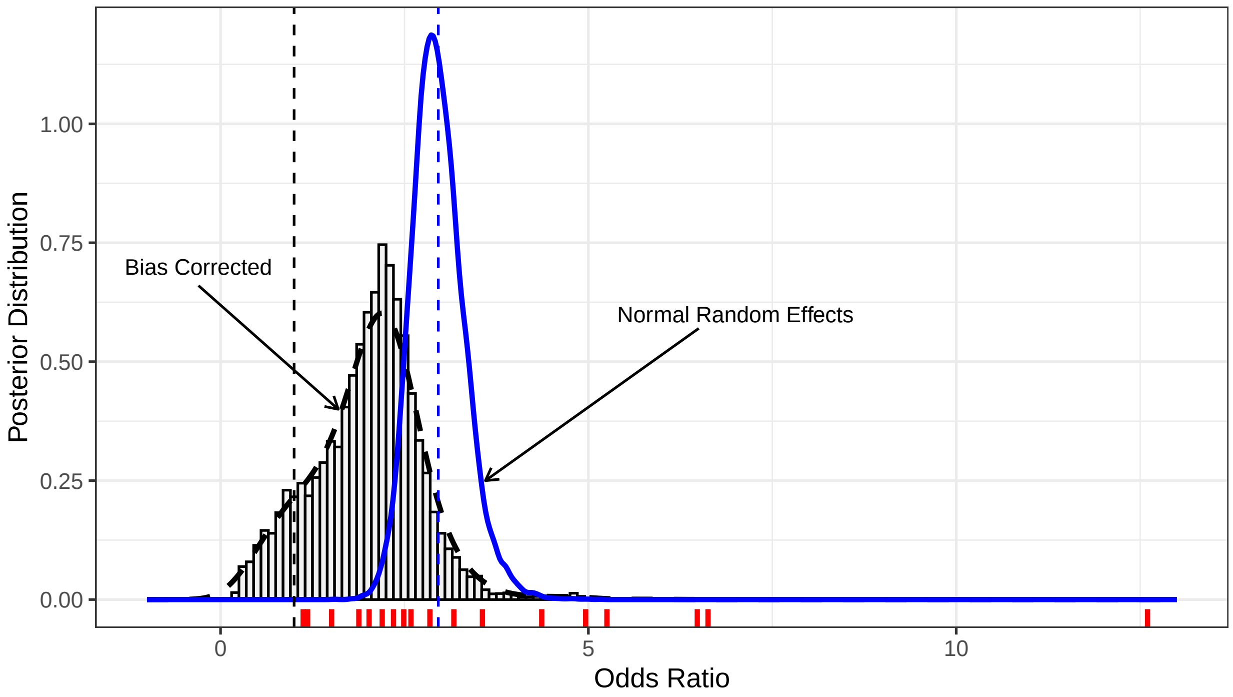

2.1 Text Section 2.1

Prior for the standard deviation parameters

```r
S = 0.5

pht(1*S, nu = 1, sigma = 0.5)
```

```
## [1] 0.5
```

```r
round(1-pht(4*S, nu = 1, sigma = 0.5),2)
```

```
## [1] 0.16
```

2.2 Results for Section 4.2

```r
# BC-BNP model ...
# Default priors ...
attach.jags(bcmix.2.covid, overwrite = TRUE)

OR = exp(mu.0)
round(mean(OR), 2)
```

```
## [1] 2.57
```

```r
round( quantile(OR, prob = c(0.025, 0.5, 0.975)), 2)
```

```
##  2.5%   50% 97.5%
##  1.60  2.51  3.86
```

```r
# BC-Parametric model ...
# Default priors ...
attach.jags(bc.covid, overwrite = TRUE)

OR.bc = exp(mu[ , 1])

round(mean(OR.bc), 2)
```

```
## [1] 2.6
```

```r
round(quantile(OR.bc, prob = c(0.025, 0.5, 0.975)),2 )
```

```
##  2.5%   50% 97.5%
##  1.96  2.54  3.53
```

```r
# Informative priors
# Posterior of K ...
attach.jags(bcmix.1.covid, overwrite = TRUE)

quantile(K.hat, prob = c(0.025, 0.5, 0.975))
```

```
##  2.5%   50% 97.5%
##     1     4     8
```

```r
# Default priors
# Posterior of K ...
attach.jags(bcmix.2.covid, overwrite = TRUE)

quantile(K.hat, prob = c(0.025, 0.5, 0.975))
```

```
##  2.5%   50% 97.5%
##     1     2     5
```

2.2.1 Figure 10

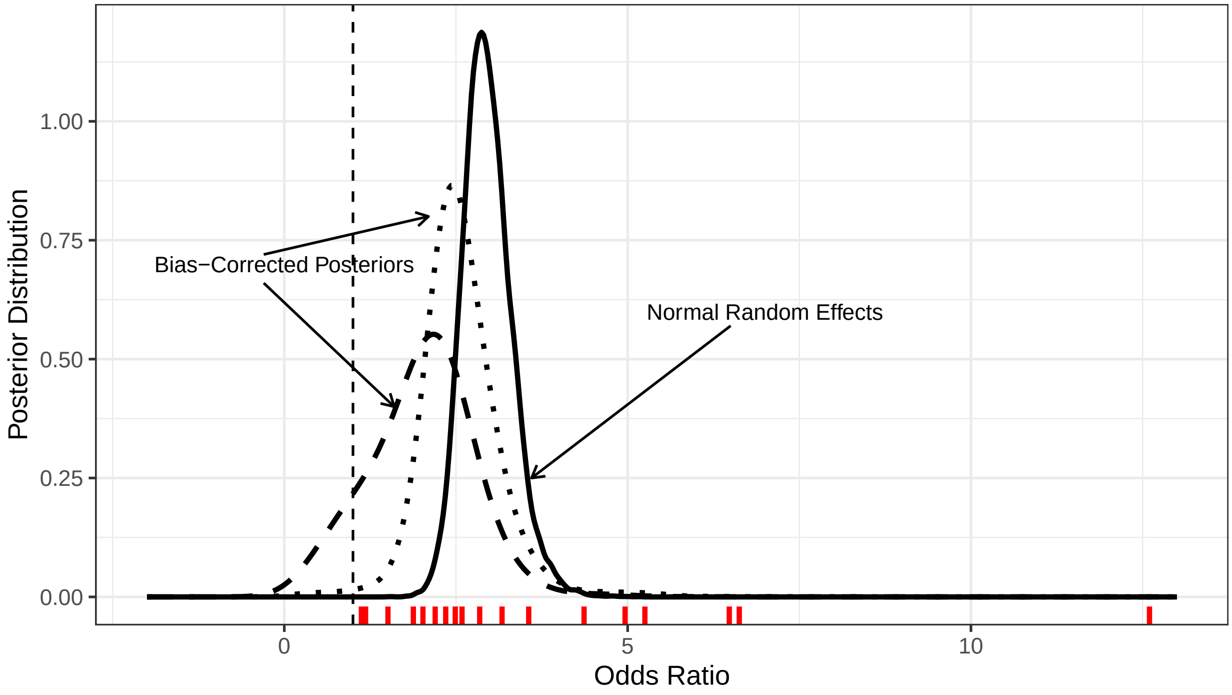

2.2.2 Figure 11: diagnostic plots

COVID-19 infected patients case study. Sensitivity analysis of the priors for the bias component in the BC-BNP model: Joint posterior distribution of the mean bias and probability of bias. The scatter plots correspond to random samples from the MCMC iterations.Left panel: Using informative prior distributions has concentrated the range of the posterior distributions. Right panel: Effect of using default distributions for the bias direction and the probability of bias.

Priors−to−Posteriors Sensitivity Analysis

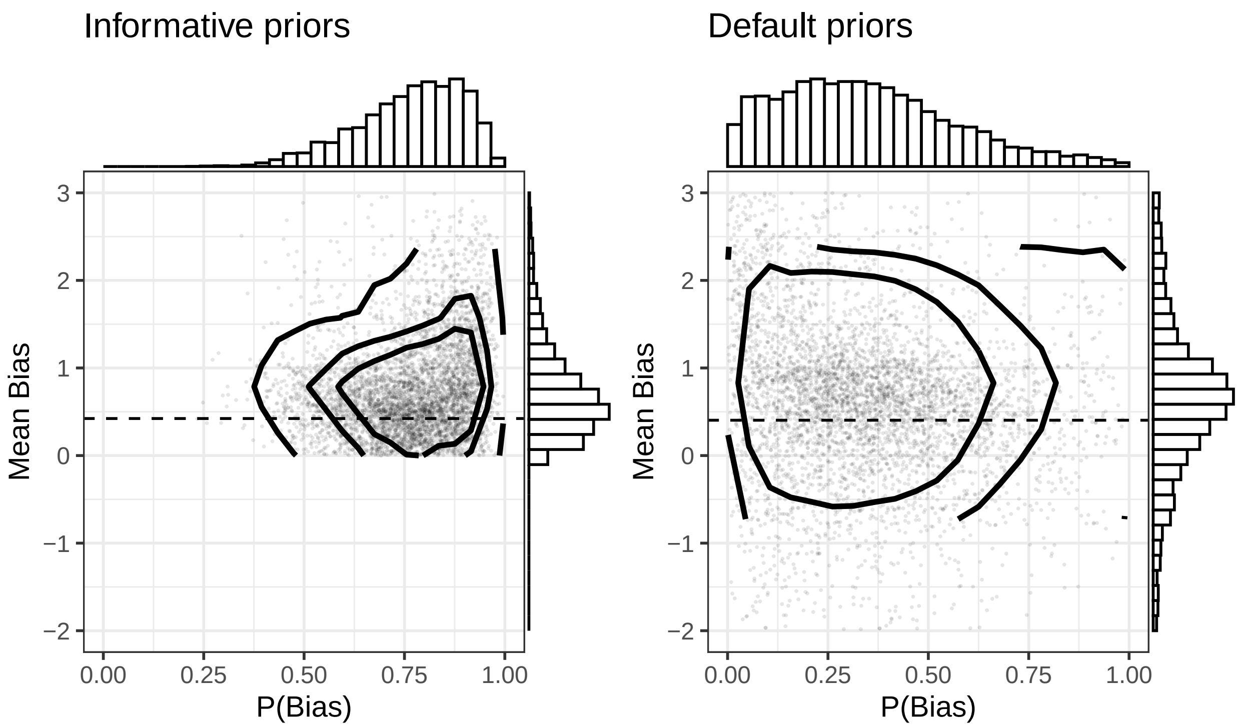

2.2.3 Figure 12: Forest plot informative priors comparing θ^B and θ

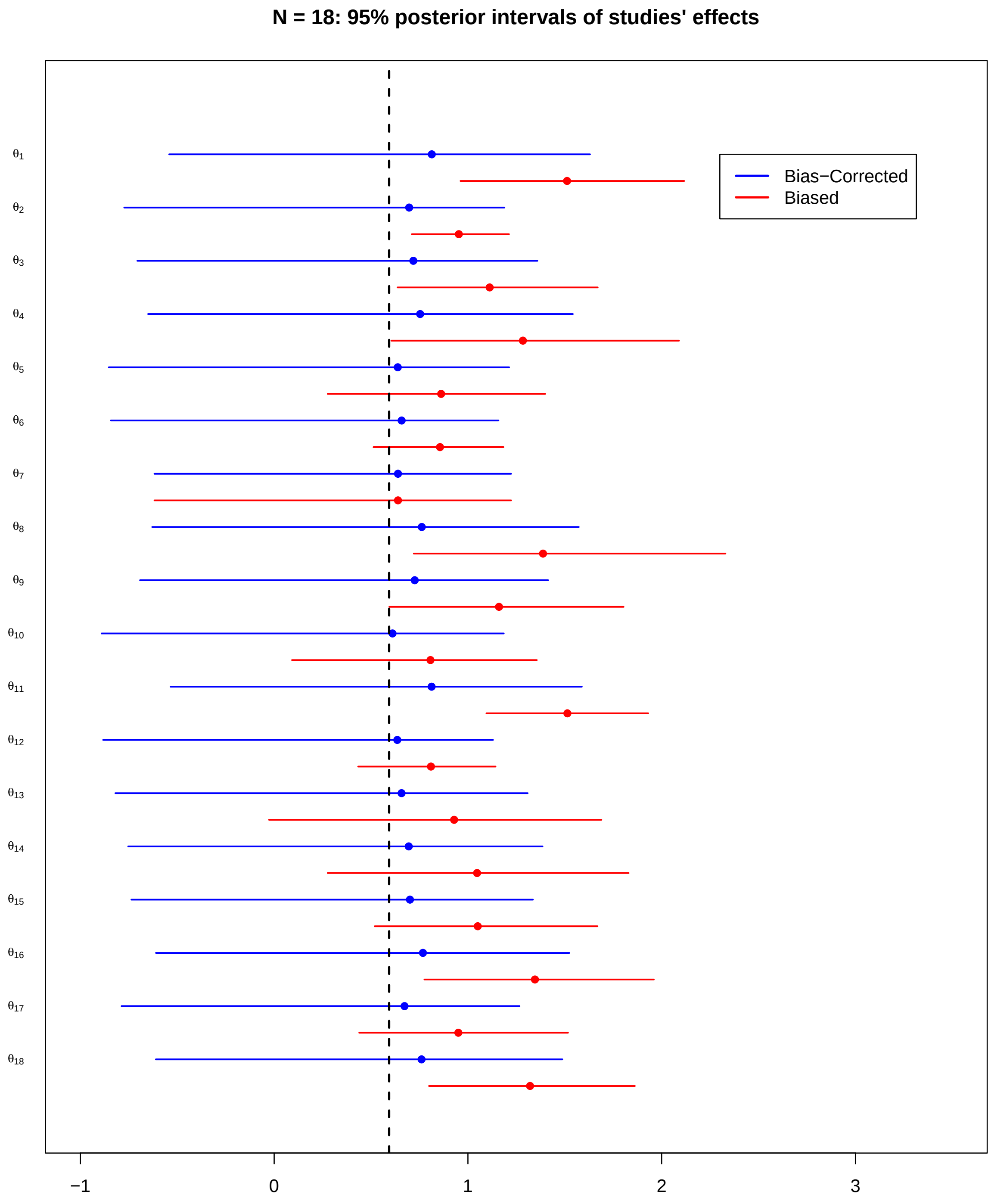


```
# mean biased corrected: location of the vertical line in the forest plot
attach.jags(bcmix.1.covid, overwrite = TRUE)

round(mean(mu.0),2)
```

```
## [1] 0.59
```

2.2.4 Individual cases informative priors

These are the posteriors of I_i for studies number 1, 8, and 18.

```r
# default priors
attach.jags(bcmix.1.covid, overwrite = TRUE)
round(apply(I[,c(1,8,18)], 2, mean),2)
```

```
## [1] 0.95 1.00 0.90
```

2.2.5 Figure 13: Co-clustering analysis with default priors

```r
require(pheatmap)
require(RColorBrewer)

# Default priors ...

attach.jags(bcmix.2.covid, overwrite = TRUE)
distance.studies.2 = apply(equalsmatrix.bias.2, c(2,3), mean)

rownames(distance.studies.2)= paste(round(apply(new.group, 2, mean),0),
                                    hyper.severity$author,
                                    sep = " | ")

colnames(distance.studies.2)= round(apply(I, 2, mean),3)

#distance.studies.2

pheatmap(distance.studies.2, color=brewer.pal(9, "Blues"), fontsize_row =7,
              fontsize_col = 6, angle_col =45,
              main = "Default priors",
              treeheight_row=0,
              treeheight_col=0,
              clustering_distance_cols = as.dist(1 - distance.studies.2),
              clustering_distance_rows = as.dist(1 - distance.studies.2))
```

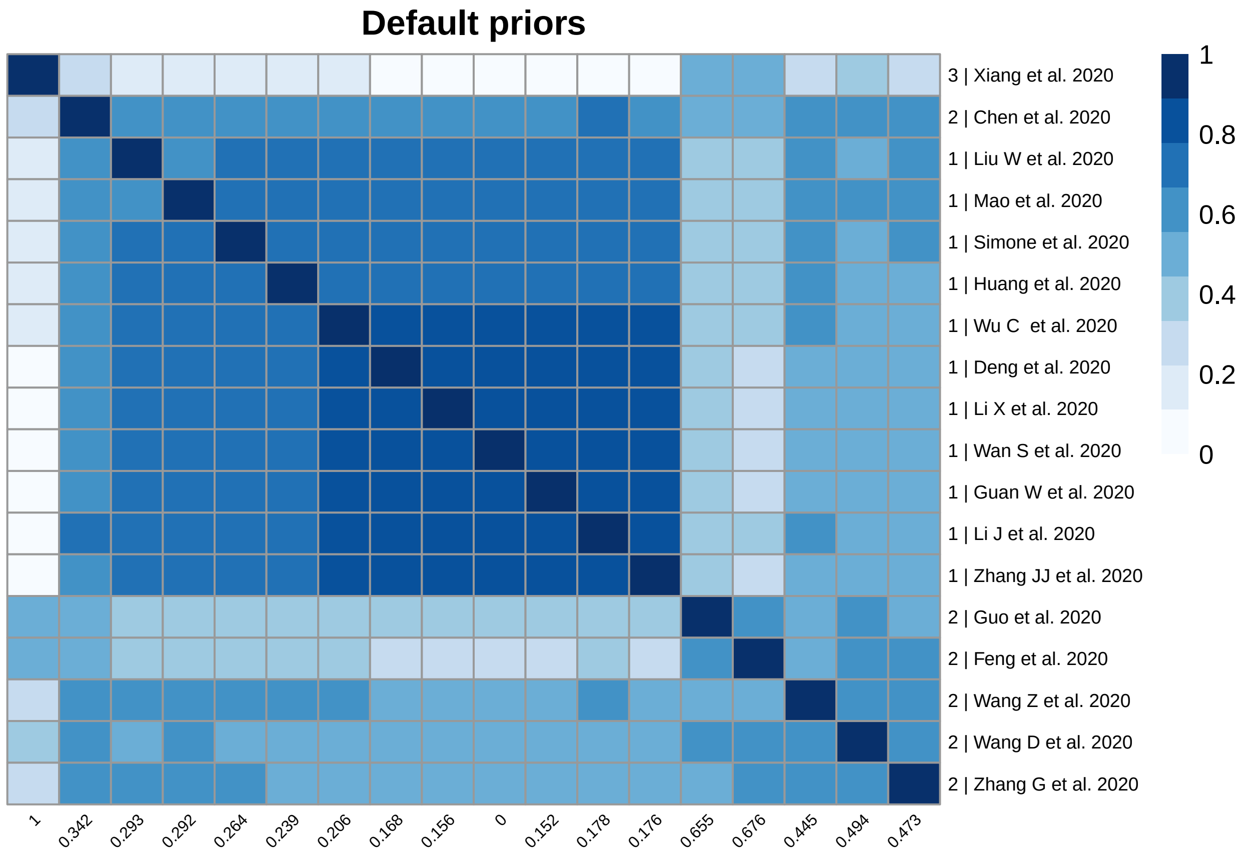
Default priors
3 | Xiang et al. 2020
2 | Chen et al. 2020
1 | Liu W et al. 2020
1 | Mao et al. 2020
1 | Simone et al. 2020
1 | Huang et al. 2020
1 | Wu C et al. 2020
1 | Deng et al. 2020
1 | Li X et al. 2020
1 | Wan S et al. 2020
1 | Guan W et al. 2020
1 | Li J et al. 2020
1 | Zhang JJ et al. 2020
2 | Guo et al. 2020
2 | Feng et al. 2020
2 | Wang Z et al. 2020
2 | Wang D et al. 2020
2 | Zhang G et al. 2020
1
0.342
0.293
0.292
0.264
0.239
0.206
0.168
0.156
0
0.152
0.178
0.176
0.655
0.676
0.445
0.494
0.473
1
0.8
0.6
0.4
0.2
0

3 Results of the stem cells example

3.0.1 Results of Section 4.1

Bayesian random effects model ...

```
summary(m.0.stemcells)
```

```
## Model specifications:
##   Link function: Normal approximation
##
##   Hyper-priors parameters:
##   Prior for mu: Normal[0, 1e+12]
##   Prior for 1/tau^2: Scale.Gamma[0.5, 1]
## Posterior distributions:
##                           mean    sd  2.5%   25%  50%  75% 97.5% Rhat n.eff
## Mean (Pooled mean)        2.92 0.743  1.43 2.426 2.92 3.41  4.37    1 12000
## Predictive effect         2.90 3.549 -4.09 0.581 2.90 5.20  9.93    1  9200
## Tau (between studies sd) 3.44 0.617  2.40 3.005 3.37 3.81  4.80    1 15000
##
## -------------------
## MCMC setup (fit using jags):  2  chains, each with  50000  iterations
## (first 10000 discarded )
## DIC: 160.799
## pD: 22.411
```

Informative priors ...

```
attach.jags(bcmix.1.stemcell, overwrite = TRUE)

# summary for mu_theta
round(mean(mu.0), 2)
```

```
## [1] 1.12
```

```
round(quantile(mu.0, prob=c(0.025, 0.975)), 2)
```

```
##  2.5% 97.5%
## -2.29  2.92
```

```
# summary for K

quantile(K.hat, prob=c(0.025, 0.5, 0.975))
```

```
##  2.5%   50% 97.5%
##     2     6    10
```

Default priors ...

```
attach.jags(bcmix.2.stemcell, overwrite = TRUE)

# summary for mu_theta
round(mean(mu.0), 2)
```

```
## [1] 1.51
```

```
round(quantile(mu.0, prob=c(0.025, 0.975)), 2)
```

```
##  2.5% 97.5%
## -0.74  3.17
```

```r
# summary for K

quantile(K.hat, prob=c(0.025, 0.5, 0.975))
```

```
##  2.5%   50% 97.5% 
##     1     4     8
```

Parametric BC

```r
summary(bc.stemcell)
```

```
## Model specifications:
##   Link function: Normal approximation
## 
##   Hyper-priors parameters: 
##   Prior for mu: Normal[0, 100]
##   Prior bias interval: Uniform[-15, 15]
##   Prior for 1/tau^2: Scale.Gamma[0.5, 1]
##   Prior bias probability: Beta[0.5, 1]
##   Prior nu: 0.5
## 
## Posterior distributions: 
##                                 mean    sd   2.5%   25%   50%   75%  97.5% Rhat
## Mean (corrected)               1.454 0.975 -1.274 1.118 1.608 2.012  2.991 1.01
## Predictive effect (corrected) 1.460 1.745 -2.027 0.506 1.523 2.357  5.165 1.01
## Tau (between studies sd)      1.212 0.753  0.219 0.683 1.049 1.543  3.217 1.01
## Mean bias                     6.039 2.641  0.847 4.599 6.134 7.594 11.122 1.02
## Prob. biased class            0.291 0.186  0.049 0.161 0.244 0.366  0.786 1.01
##                               n.eff
## Mean (corrected)                510
## Predictive effect (corrected)  1400
## Tau (between studies sd)        560
## Mean bias                      1100
## Prob. biased class              340
## 
## -------------------
## MCMC setup (fit using jags): 4 chains, each with 50000 iterations (first 10000 discarded)
## DIC: 184.02
## pD: 20.067
```

3.0.2 Figure 6 of Section 4.1

Posteriors for μ_θ ...

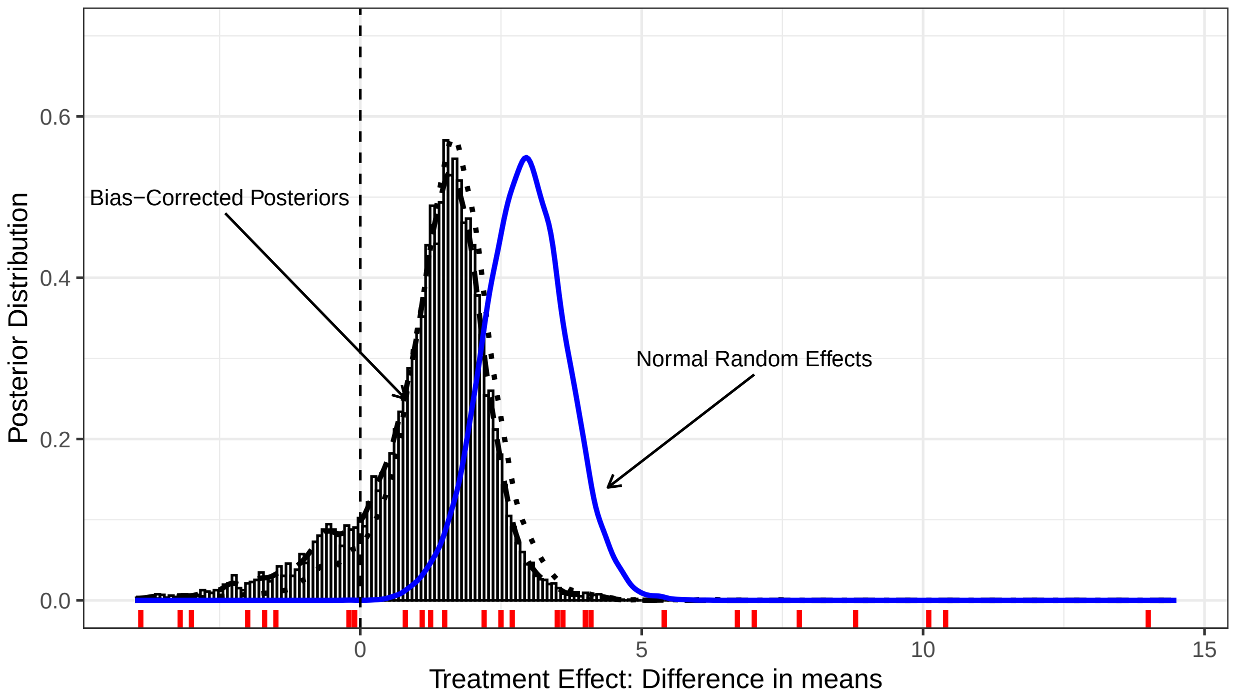

3.0.3 Figure 7 of Section 4.1

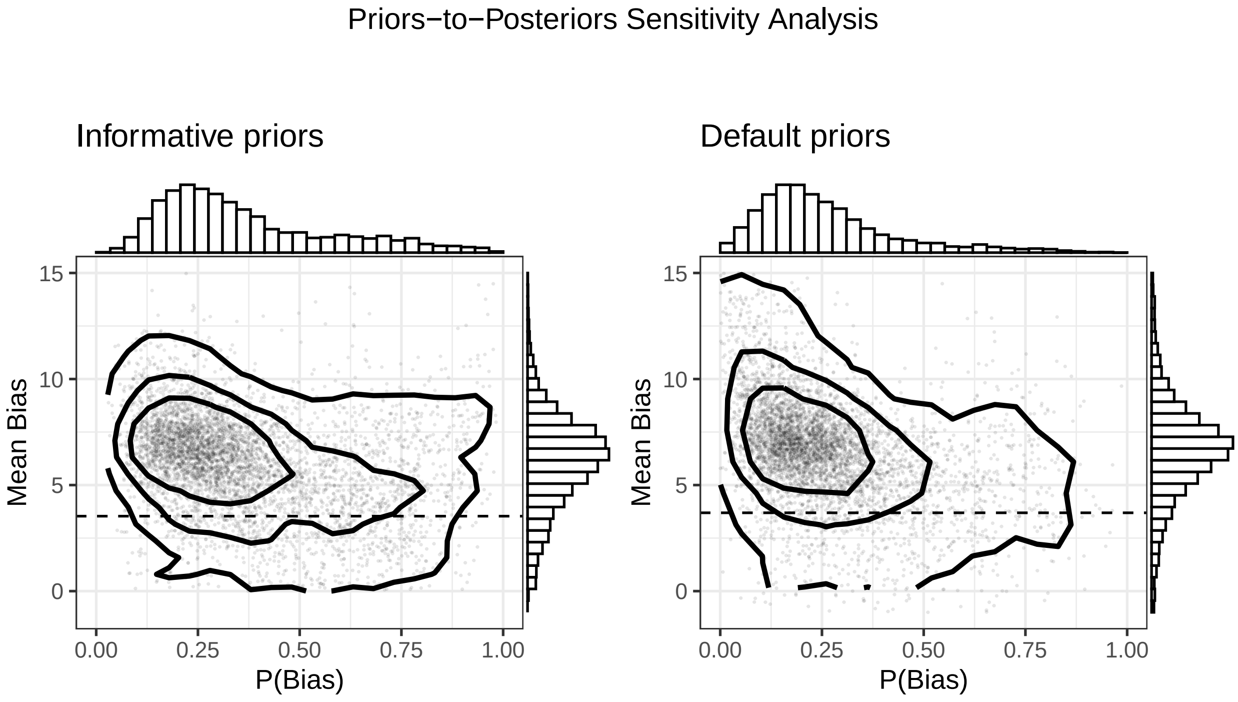

3.0.4 Figure 8 of Section 4.1: Forest plot default priors comparing θ^B and θ

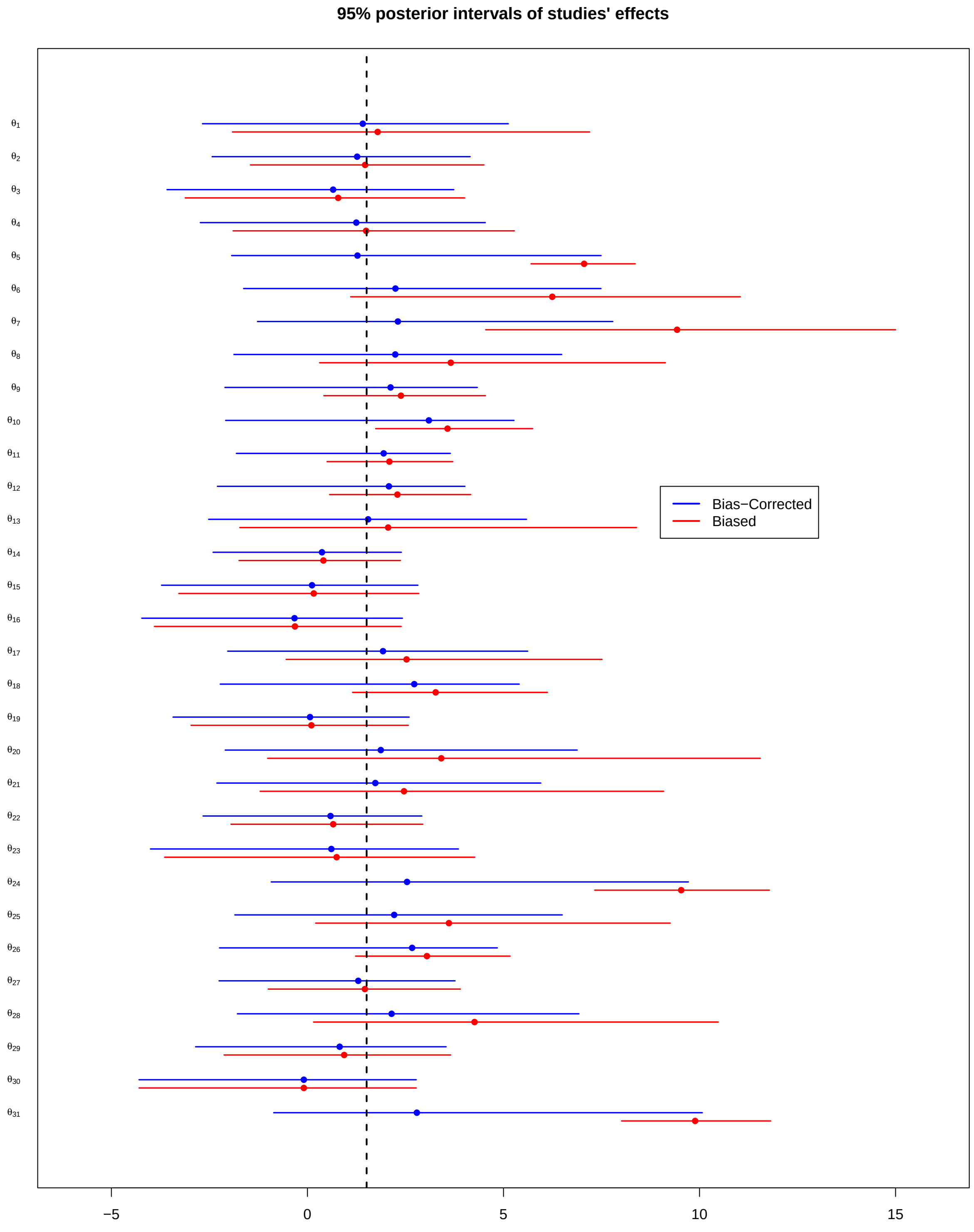

3.0.5 Four cases informative priors

These are the posteriors of I_i for studies number 1,5,24, and 31.

```r
attach.jags(bcmix.1.stemcell, overwrite = TRUE)
round(apply(I[,c(1,5,24, 31)], 2, mean),2)
```

```
## [1] 0.24 0.88 0.96 0.96
```

3.0.6 Figure 9 of Section 4.1

```r
require(pheatmap)
require(RColorBrewer)

# Default priors ...

attach.jags(bcmix.2.stemcell, overwrite = TRUE)
distance.studies.2 = apply(equalsmatrix.bias.2, c(2,3), mean)

rownames(distance.studies.2)= paste(round(apply(new.group, 2, mean),0),
                                    stemcells$trial,
                                    sep = "|")

colnames(distance.studies.2)= round(apply(I, 2, mean),3)

pheatmap(distance.studies.2,color=brewer.pal(9,"Blues"), fontsize_row =7,
              fontsize_col = 6, angle_col =45,
              main = "Default priors",
              treeheight_row=0,
              treeheight_col=0,
              clustering_distance_cols = as.dist(1 - distance.studies.2),
              clustering_distance_rows = as.dist(1 - distance.studies.2)
              )
```

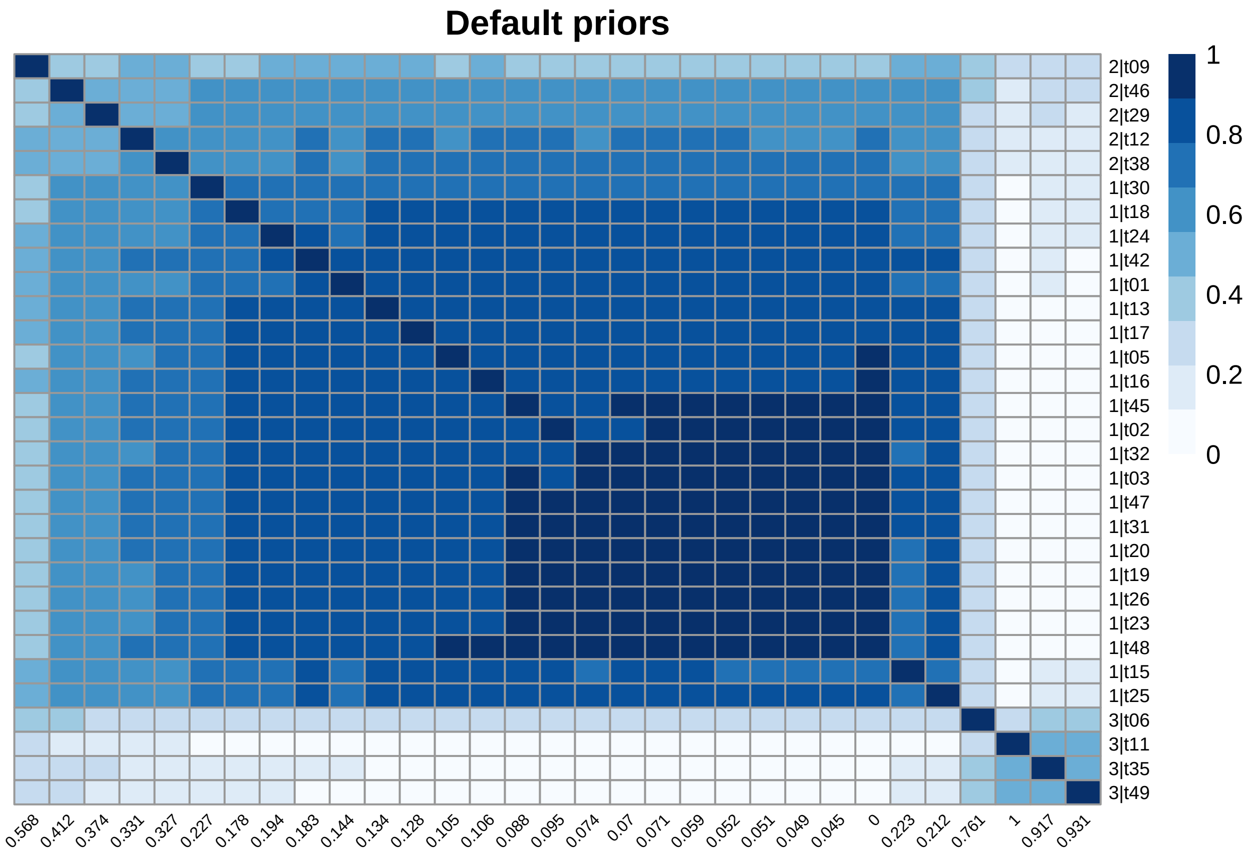

3.0.7 Interpretation about co-clustering based on the Risk of Bias evaluation

- We list the mean clustering label with the Risk of Bias evaluated by the authors:

```
# Default priors ...

attach.jags(bcmix.2.stemcell, overwrite = TRUE)

stem.risk = data.frame(trial = stemcells$trial,
           p.bias = round(apply(I, 2, mean),3),
          cluster = round(apply(new.group, 2, mean),0),
          n.discrepancies = stemcells$n.discrep,
          stemcells[,6:11])

stem.risk %>% arrange(cluster)
```

```
##    trial p.bias cluster n.discrepancies Sequence Allocation Blinding Outcome
## 1    t01  0.144       1              17  Unclear    Unclear  Unclear Unclear
## 2    t02  0.095       1               7      Yes        Yes       No     Yes
## 3    t03  0.070       1               7  Unclear    Unclear  Unclear     Yes
## 4    t05  0.105       1               4       No        Yes       No Unclear
## 5    t13  0.134       1              16      Yes    Unclear      Yes Unclear
## 6    t15  0.223       1               0  Unclear    Unclear       No Unclear
## 7    t16  0.106       1              27      Yes         No      Yes     Yes
## 8    t17  0.128       1              11  Unclear    Unclear      Yes Unclear
## 9    t18  0.178       1               2  Unclear        Yes       No Unclear
## 10   t19  0.051       1               3      Yes        Yes       No     Yes
```

```
## 11   t20  0.052       1              0     Yes    Unclear      Yes     Yes
## 12   t23  0.045       1              0  Unclear    Unclear      Yes     Yes
## 13   t24  0.194       1              2  Unclear    Unclear      Yes Unclear
## 14   t25  0.212       1              3  Unclear        Yes      Yes     Yes
## 15   t26  0.049       1              8     Yes        Yes      Yes Unclear
## 16   t30  0.227       1              2  Unclear    Unclear       No     Yes
## 17   t31  0.059       1             19     Yes        Yes      Yes     Yes
## 18   t32  0.074       1              7  Unclear    Unclear      Yes     Yes
## 19   t42  0.183       1              6     Yes        Yes  Unclear Unclear
## 20   t45  0.088       1              9  Unclear    Unclear       No      No
## 21   t47  0.071       1              2  Unclear    Unclear      Yes      No
## 22   t48  0.000       1              1     Yes    Unclear      Yes     Yes
## 23   t09  0.568       2             21  Unclear    Unclear       No Unclear
## 24   t12  0.331       2             18     Yes        Yes      Yes Unclear
## 25   t29  0.374       2              6  Unclear    Unclear       No      No
## 26   t38  0.327       2             15     Yes        Yes      Yes     Yes
## 27   t46  0.412       2              2  Unclear        Yes      Yes     Yes
## 28   t06  0.761       3              4  Unclear    Unclear       No      No
## 29   t11  1.000       3             13  Unclear        Yes       No     Yes
## 30   t35  0.917       3             11       No    Unclear       No     Yes
## 31   t49  0.931       3             55  Unclear    Unclear       No Unclear
##    Reporting Other
## 1    Unclear   Yes
## 2        Yes   Yes
## 3    Unclear   Yes
## 4        Yes   Yes
## 5        Yes   Yes
## 6        Yes   Yes
## 7        Yes   Yes
## 8        Yes   Yes
## 9    Unclear   Yes
## 10       Yes   Yes
## 11       Yes   Yes
## 12       Yes   Yes
## 13       Yes   Yes
## 14       Yes   Yes
## 15       Yes   Yes
## 16       Yes   Yes
## 17       Yes   Yes
## 18        No   Yes
## 19   Unclear   Yes
## 20       Yes   Yes
## 21       Yes   Yes
## 22       Yes   Yes
## 23       Yes   Yes
## 24   Unclear   Yes
## 25   Unclear   Yes
## 26       Yes   Yes
## 27        No   Yes
## 28   Unclear   Yes
## 29   Unclear   Yes
## 30        No   Yes
## 31   Unclear    No
```

- Newbar et al. (2014) reported that the lack of information about sequence allocation in the Risk of Bias was linked with exaggerated treatment effects.

```
# Information about sequence allocation (Randomization)
tab.sequence.allocation = table(stem.risk$Sequence, stem.risk$cluster)
tab.sequence.allocation
```

```
##
##            1  2  3
##   No       1  0  1
##   Unclear 12  3  3
##   Yes      9  2  0
```

```
round(prop.table(tab.sequence.allocation,2),2)
```

```
##
##              1    2    3
##   No      0.05 0.00 0.25
##   Unclear 0.55 0.60 0.75
##   Yes     0.41 0.40 0.00
```

```
tab.discrep = table(stem.risk$n.discrepancies,
      stem.risk$cluster)
tab.discrep
```

```
##
##       1 2 3
##   0   3 0 0
##   1   1 0 0
##   2   4 1 0
##   3   2 0 0
##   4   1 0 1
##   6   1 1 0
##   7   3 0 0
##   8   1 0 0
##   9   1 0 0
##   11  1 0 1
##   13  0 0 1
##   15  0 1 0
##   16  1 0 0
##   17  1 0 0
##   18  0 1 0
##   19  1 0 0
##   21  0 1 0
##   27  1 0 0
##   55  0 0 1
```

```
prop.discrep = round(prop.table(tab.discrep,2),2)
prop.discrep
```

```
##
##          1    2    3
##   0   0.14 0.00 0.00
##   1   0.05 0.00 0.00
##   2   0.18 0.20 0.00
##   3   0.09 0.00 0.00
##   4   0.05 0.00 0.25
```

```
##   6  0.05 0.20 0.00
##   7  0.14 0.00 0.00
##   8  0.05 0.00 0.00
##   9  0.05 0.00 0.00
##   11 0.05 0.00 0.25
##   13 0.00 0.00 0.25
##   15 0.00 0.20 0.00
##   16 0.05 0.00 0.00
##   17 0.05 0.00 0.00
##   18 0.00 0.20 0.00
##   19 0.05 0.00 0.00
##   21 0.00 0.20 0.00
##   27 0.05 0.00 0.00
##   55 0.00 0.00 0.25
```

```r
time.end = Sys.time()
#Total time to run the script
difference = difftime(time.end, time.start, units='mins')
difference
```

```
## Time difference of 24.1 mins
```

```r
sessionInfo()
```

```
## R version 4.4.2 (2024-10-31 ucrt)
## Platform: x86_64-w64-mingw32/x64
## Running under: Windows 11 x64 (build 22631)
##
## Matrix products: default
##
##
## locale:
## [1] LC_COLLATE=German_Germany.utf8  LC_CTYPE=German_Germany.utf8
## [3] LC_MONETARY=German_Germany.utf8 LC_NUMERIC=C
## [5] LC_TIME=German_Germany.utf8
##
## time zone: Europe/Berlin
## tzcode source: internal
##
## attached base packages:
## [1] grid      stats     graphics  grDevices utils     datasets  methods
## [8] base
##
## other attached packages:
##  [1] RColorBrewer_1.1-3 pheatmap_1.0.12    LearnBayes_2.15.1  readxl_1.4.3
##  [5] xtable_1.8-4       extraDistr_1.10.0  jarbes_2.2.3       GGally_2.2.1
##  [9] R2jags_0.8-9       rjags_4-16         mcmcplots_0.4.3    coda_0.19-4.1
## [13] gridExtra_2.3      lubridate_1.9.3    forcats_1.0.0      stringr_1.5.1
## [17] dplyr_1.1.4        purrr_1.0.2        readr_2.1.5        tidyr_1.3.1
## [21] tibble_3.2.1       ggplot2_3.5.1      tidyverse_2.0.0
##
## loaded via a namespace (and not attached):
##  [1] denstrip_1.5.4     gtable_0.3.6       xfun_0.49          lattice_0.22-6
##  [5] tzdb_0.4.0         vctrs_0.6.5        tools_4.4.2        generics_0.1.3
##  [9] parallel_4.4.2     fansi_1.0.6        pkgconfig_2.0.3    lifecycle_1.0.4
## [13] farver_2.1.2       compiler_4.4.2     munsell_0.5.1      httpuv_1.6.15
```

```
## [17] htmltools_0.5.8.1  yaml_2.3.10        pillar_1.9.0       later_1.3.2
## [21] MASS_7.3-61        boot_1.3-31        abind_1.4-8        mime_0.12
## [25] ggstats_0.7.0      tidyselect_1.2.1   digest_0.6.37      stringi_1.8.4
## [29] labeling_0.4.3     fastmap_1.2.0      colorspace_2.1-1   cli_3.6.3
## [33] magrittr_2.0.3     utf8_1.2.4         withr_3.0.2        scales_1.3.0
## [37] promises_1.3.0     timechange_0.3.0   rmarkdown_2.29     ggExtra_0.10.1
## [41] cellranger_1.1.0   hms_1.1.3          shiny_1.9.1        evaluate_1.0.1
## [45] knitr_1.49         miniUI_0.1.1.1     rlang_1.1.4        isoband_0.2.7
## [49] Rcpp_1.0.13-1      glue_1.8.0         rstudioapi_0.17.1  R6_2.5.1
## [53] plyr_1.8.9         R2WinBUGS_2.1-22.1 sfsmisc_1.1-20
```
