# Supplementary material for: A Bias‐Corrected Bayesian Nonparametric Model for Combining Studies With Varying Quality in Meta‐Analysis
Source: Biom J. 2025 Feb 7;67(1):e70034. doi: 10.1002/bimj.70034 (PMC11803498; doi:10.1002/bimj.70034)
Supplement: Supplementary file 1 — Supporting Information [file BIMJ-67-e70034-s001.zip › Data_and_Software/Simulations-Section-3/BC-BNP-Section-3-Simulations-January-2025.pdf]

A Bias-Corrected Bayesian Nonparametric Model for Combining Studies with Varying Quality in Meta-Analysis

Simulated Examples for Section 3

Pablo E. Verde

and

Gary L. Rosner

08 Januar 2025

Contents

1 Distribution of the sample sizes of the studies 3
1.1 Sample sizes for N=20 taken from the COVID-19 example: hypertension severity 3
1.1.1 Empirical determination of the within standard deviation σ 3
1.2 Sample sizes for N = 30 from the stem-cell example** 3

2 Definition of biased studies 5
2.1 Parameter settings 5
2.2 Bias locations for N=20 6
2.3 Bias locations for N=30 6

3 Function to simulate a data for a meta-analysis 8

4 Different scenarios: 10

5 Generate data sets for meta-analysis 11
5.0.1 sim.20.0 11
5.0.2 sim.20.10 11
5.0.3 sim.20.30 11
5.0.4 sim.20.50 11
5.0.5 sim.30.0 11
5.0.6 sim.30.10 12
5.0.7 sim.30.30 12
5.0.8 sim.30.50 12

6 Bayesian RE meta-analysis 12

7 Oracle Bayesian RE meta-analysis 12

8 BC Parametric 13
8.1 Prior for the probability of B 13

9 BC-BNP 13
9.1 Prior for the probability of bias 13

9.2 Determination of maximum number of clusters and prior for α . . . 13

10 Figures for Section 3 **15**
10.1 Comparision of different models N = 20 . . . 15
10.1.1 Bayesian RE . . . 15
10.1.2 Oracle Bayesian RE . . . 16
10.1.3 BC parametric . . . 17
10.1.4 BC-BNP . . . 18
10.1.5 All models together . . . 19
10.1.6 Forest plot: comparing θ^B and θ . . . 21
10.1.7 Example of 4 studies: without bias and with bias . . . 22
10.2 Summary of BC-BNP for N=20 and B=6 . . . 25
10.3 Comparison of different models N = 30 . . . 26
10.3.1 Bayesian RE . . . 26
10.3.2 Oracle Bayesian RE . . . 27
10.3.3 BC parametric . . . 28
10.3.4 BC-BNP . . . 29
10.3.5 All models together . . . 30

11 Clusters report n=20 with 50% bias **32**
11.1 Co-clustering Posterior probability study i shares the same cluster with j . . . 32

12 Clusters report n=30 with 50% bias **34**
12.1 Co-clustering Posterior probability study i shares the same cluster with j . . . 34

13 Session information **35**

1 Distribution of the sample sizes of the studies

For n_i $(i = 1, \ldots, N)$ we use a random sample from each of the examples in Section 4.

1.1 Sample sizes for N=20 taken from the COVID-19 example: hypertension severity

```
set.seed(042024)
hyper.severity = covid19 %>%
                 filter(
                  risk.factor=="hypertension"&
                  endpoint=="severity") %>%
                 select(author, design,TE, seTE,N) %>% arrange(design)


n.i.covid = hyper.severity$N
summary(n.i.covid)
```

```
##    Min. 1st Qu.  Median    Mean 3rd Qu.    Max.
##      41     115     145     287     219    1178
```

```
n.i.20 = sample(n.i.covid, size = 20, replace = TRUE)

summary(n.i.20)
```

```
##    Min. 1st Qu.  Median    Mean 3rd Qu.    Max.
##      49     129     140     319     303    1178
```

```
mean.n.20 = mean(n.i.20)
mean.n.20
```

```
## [1] 319.15
```

1.1.1 Empirical determination of the within standard deviation σ

We assume asymptotically that $\mathrm{SE}_i^2 = \sigma^2/n_i$, and we calculate the possible values of σ.

```
summary(hyper.severity$seTE*sqrt(hyper.severity$N))
```

```
##    Min. 1st Qu.  Median    Mean 3rd Qu.    Max.
##    4.37    4.60    4.95    5.35    5.92    7.26
```

$$\sigma \approx 4.95$$

1.2 Sample sizes for N = 30 from the stem-cell example**

```
set.seed(24052024)
data("stemcells")
n.i.stemcell = stemcells$sample.size
summary(n.i.stemcell)
```

```
##    Min. 1st Qu.  Median    Mean 3rd Qu.    Max.
##    10.0    20.5    40.0    62.3    84.0   187.0
```

```r
n.i.30 = sample(n.i.stemcell, size = 30, replace = TRUE)

summary(n.i.30)
```

```
##    Min. 1st Qu.  Median    Mean 3rd Qu.    Max.
##    14.0    21.0    40.0    59.3    79.0   187.0
```

```r
mean.n.30 = mean(n.i.30)
mean.n.30
```

```
## [1] 59.2667
```

2 Definition of biased studies

From the marginal model of y_i we use the following relationship between σ_y and the IQR (interquartile rate):

$$IQR = 1.35\,\sigma_y.$$

Let Q_3 be the 75% quartile of the marginal distribution of y_i, and

- Upper inner fence: Q3 + 1.5*IQR
- In between fences: Q3 + 2.25*IQR
- Upper outer fence: Q3 + 3*IQR.

We define **mild B**, **large B** and **extreme B** results, those values of y_i such us:

- Mild B: $Q3 + 1.5 * IQR < y_i^{\text{mild}} < Q3 + 2.25 * IQR$
- Large B: $Q3 + 2.25 * IQR < y_i^{\text{large}} < Q3 + 3 * IQR$
- Extreme B: $Q3 + 3 * IQR < y_i^{\text{extreme}}$.

2.1 Parameter settings

- Set $\mu = 1$ and $\sigma = 4.95$
- Set $\tau = 0.5$ => **moderate heterogeneity** in the log(OR) scale.
- Studies sample sizes n_i is a random sample from the examples.

$$\mu_\beta^M = \mathrm{Q}_3 + 1.5\,\mathrm{IQR}, \quad \mu_\beta^L = \mathrm{Q}_3 + 2.25\,\mathrm{IQR}, \quad \text{and} \quad \mu_\beta^E = \mathrm{Q}_3 + 3.0\,\mathrm{IQR}.$$

2.2 Bias locations for N=20

```
Sigma = 4.95
mean.y = 1
var.y = Sigma^2/mean.n.20 + 0.5^2

sigma.y = sqrt(var.y)

Q3 = qnorm(0.75, mean = mean.y, sd = sigma.y)

IQR = 1.35*sigma.y

# Upper inner fence
inner.fence = Q3 + 1.5*IQR

# Upper outer fence:
outer.fence = Q3 + 3*IQR

# Between fences
between.fence = (inner.fence+outer.fence)/2

# Means contamination ...

mu.beta.mild.20  = (inner.fence)
mu.beta.extreme.20 = (outer.fence)
mu.beta.large.20 = (between.fence)

mu.beta.mild.20
```

```
## [1] 2.54314
```

```
mu.beta.large.20
```

```
## [1] 3.12193
```

```
mu.beta.extreme.20
```

```
## [1] 3.70072
```

The location of the three mean B μ_β^M, μ_β^L, and μ_β^E are: 2.543141, 3.121928, and 3.700715.

2.3 Bias locations for N=30

```
Sigma = 4.95
mean.y = 1
var.y = Sigma^2/mean.n.30 + 0.5^2

sigma.y = sqrt(var.y)

Q3 = qnorm(0.75, mean = mean.y, sd = sigma.y)

IQR = 1.35*sigma.y

# Upper inner fence
inner.fence = Q3 + 1.5*IQR
```

```r
# Upper outer fence:
outer.fence = Q3 + 3*IQR

# Between fences
between.fence = (inner.fence+outer.fence)/2

# Means contamination ...

mu.beta.mild.30  = (inner.fence)
mu.beta.extreme.30 = (outer.fence)
mu.beta.large.30 = (between.fence)

mu.beta.mild.30
```

```
## [1] 3.19876
```

```r
mu.beta.large.30
```

```
## [1] 4.02346
```

```r
mu.beta.extreme.30
```

```
## [1] 4.84815
```

The location of the three mean B μ_β^M, μ_β^L, and μ_β^E are: 3.198764, 4.023456, and 4.848148.

3 Function to simulate a data for a meta-analysis

```
# Function simData
#
# This function simulates aggregated data for a meta-analysis of N studies.
#
# The simulation model utilizes normal likelihoods and incorporates
# normal random effects. After simulating the studies, a random sample is selected,
# and these studies are subjected to contamination at three bias levels: mild, large, and extreme.
#
# Arguments:
#
# mu:           scalar with the true pooled effect value.
# sigma:        scalar with the true intra-study standard deviation
# n.total:      a vector with the sample sizes of the studies.
# tau:          scalar with the between studies standard deviation.
# N:            scalar utih the total number of studies in the meta-analysis
# mu.beta.1:    scalar with the mean bias of studies in the mild bias class.
# mu.beta.2:    scalar with the mean bias of studies in the large bias class.
# mu.beta.3:    scalar with the mean bias of studies in the extreme bias class.
#
# n.B.1: scalar with the number of studies in the mild bias class.
# n.B.2: scalar with the number of studies in the large bias class.
# n.B.3: scalar with the number of studies in the extreme bias class.

# The function returns a dataframe with the following columns:
#
# TE:      Observed study's effect.
# seTE:    Standard error of the study's effect.
# theta:   True study's effect.
# n.total: Sample size of the study.
# B.flag:  Indicates if a study is biased, with the following possible values:
#         "No B" = No biased study.
#         "Mild B" = Mild biased study.
#         "Large B" = Large biased study.
#         "Extreme B = Extreme biased study.
#
#




simData = function(mu,
                   sigma,
                   n.total,
                   tau,
                   N,
                   mu.beta.1,
                   mu.beta.2,
                   mu.beta.3,
                   n.B.1,
                   n.B.2,
                   n.B.3)
```

```
{

     theta = rnorm(N, mean = mu, sd = tau)
      SE.y = sigma*sqrt(1/n.total)
        TE = rnorm(N, mean = theta, sd = SE.y)
     Study.id = paste("Study", 1:N, sep = "-")

# Generate Bed studies at random ...
      B.flag = rep("No B", N)

# check for any class of B and which one
if(any(c(n.B.1, n.B.2, n.B.3)>0)){
  case.B = which(c(n.B.1, n.B.2, n.B.3)>0)
  case.B = paste0(case.B, collapse = "")

# search for each possible way of B and contaminate the studies
switch(case.B,
 #.........................................................................
  "1" = {set.pick = 1:N
      pick.outlier.1 = sample(set.pick, n.B.1, replace = FALSE)
      TE[pick.outlier.1] = TE[pick.outlier.1] + mu.beta.1
  B.flag[pick.outlier.1] = "Mild B"
        },
 #.........................................................................
  "2" = {set.pick = 1:N
    pick.outlier.2 = sample(set.pick, n.B.2, replace = FALSE)
    TE[pick.outlier.2] = TE[pick.outlier.2] + mu.beta.2
   B.flag[pick.outlier.2] = "Large B"
       },
 #.........................................................................
  "3" = {set.pick = 1:N
   pick.outlier.3 = sample(set.pick, n.B.3, replace = FALSE)
   TE[pick.outlier.3] = TE[pick.outlier.3] + mu.beta.3
   B.flag[pick.outlier.3] = "Extreme B"
      },
 #.........................................................................
  "12" = {
   set.pick = 1:N
   pick.outlier.1 = sample(set.pick, n.B.1, replace = FALSE)
   set.pick = set.pick[-pick.outlier.1]
   pick.outlier.2 = sample(set.pick, n.B.2, replace = FALSE)
   TE[pick.outlier.1] = TE[pick.outlier.1] + mu.beta.1
   B.flag[pick.outlier.1] = "Mild B"
   TE[pick.outlier.2] = TE[pick.outlier.2] + mu.beta.2

  B.flag[pick.outlier.2] = "Large B"
        },
   "23" = {
   set.pick = 1:N
   pick.outlier.2 = sample(set.pick, n.B.2, replace = FALSE)
   set.pick = set.pick[-pick.outlier.2]
   pick.outlier.3 = sample(set.pick, n.B.3, replace = FALSE)
  TE[pick.outlier.2] = TE[pick.outlier.2] + mu.beta.2
```

```
    B.flag[pick.outlier.2] = "Large B"
    TE[pick.outlier.3] = TE[pick.outlier.3] + mu.beta.3
    B.flag[pick.outlier.3] = "Extreme B"
        },
    "13" = {
    set.pick = 1:N
    pick.outlier.1 = sample(set.pick, n.B.1, replace = FALSE)
    set.pick = set.pick[-pick.outlier.1]
    pick.outlier.3 = sample(set.pick, n.B.3, replace = FALSE)
    pick.outlier = c(pick.outlier.1, pick.outlier.3)

    TE[pick.outlier.1] = TE[pick.outlier.1] + mu.beta.1
    B.flag[pick.outlier.1] = "Mild B"

    TE[pick.outlier.3] = TE[pick.outlier.3] + mu.beta.3
    B.flag[pick.outlier.3] = "Extreme B"
        },
    "123" = {
    set.pick = 1:N

    pick.outlier.1 = sample(set.pick, n.B.1, replace = FALSE)
    pick.outlier.2 = sample(set.pick[-pick.outlier.1], n.B.2, replace = FALSE)
    pick.outlier.3 = sample(set.pick[-c(pick.outlier.1, pick.outlier.2)],
                            n.B.3, replace = FALSE)

    TE[pick.outlier.1] = TE[pick.outlier.1] + mu.beta.1
    B.flag[pick.outlier.1] = "Mild B"

    TE[pick.outlier.2] = TE[pick.outlier.2] + mu.beta.2
    B.flag[pick.outlier.2] = "Large B"

    TE[pick.outlier.3] = TE[pick.outlier.3] + mu.beta.3
    B.flag[pick.outlier.3] = "Extreme B"
       }
  )
} else cat("no biased studies in this simulations \n")

sims = data.frame(TE, seTE = SE.y, theta, n.total, B.flag)
rownames(sims)=Study.id
  return(sims)
}
```

4 Different scenarios:

Note: We selected the maximum contamination to 50%. In robust statistics this is the maximum break point of an estimator (e.g. median).

Number of studies N=20

% bias	Bias Studies	Mild	Large	Extreme
10%	2	0	1	1
30%	6	2	2	2
50%	10	3	3	4

Number of studies N=30

% bias	Bias Studies	Mild	Large	Extreme
10%	3	1	1	1
30%	9	3	3	3
50%	15	5	5	5

We compare the following models:

- Bayesian RE.
- BC Parametric.
- Oracle just omit biased studies.
- BC nonparametric.

5 Generate data sets for meta-analysis

Naming convention: **sim.N.percentage biased studies**

5.0.1 sim.20.0

```
## no biased studies in this simulations
```

```
##
## No B
##   20
```

5.0.2 sim.20.10

```
##
## Extreme B   Large B      No B
##         1         1        18
```

5.0.3 sim.20.30

```
##
## Extreme B   Large B    Mild B      No B
##         2         2         2        14
```

5.0.4 sim.20.50

```
##
## Extreme B   Large B    Mild B      No B
##         4         3         3        10
```

5.0.5 sim.30.0

```
## no biased studies in this simulations
```

```
##
## No B
##   30
```

5.0.6 sim.30.10

```
##
## Extreme B   Large B    Mild B      No B
##         1         1         1        27
```

5.0.7 sim.30.30

```
##
## Extreme B   Large B    Mild B      No B
##         3         3         3        21
```

5.0.8 sim.30.50

```
##
## Extreme B   Large B    Mild B      No B
##         5         5         5        15
```

6 Bayesian RE meta-analysis

7 Oracle Bayesian RE meta-analysis

8 BC Parametric

8.1 Prior for the probability of B

- We expect a-priori a small number of biased studies in the meta-analysis. We use a Beta(0.5, 1), which has:
  1) A spike close to 0
  2) A prior mean of ≈ 0.33 and the
  3) A prior probability $P(\pi_B < 0.2)$=0.105573.
- We ignore the direction of bias: The prior for the mean bias is Uniform(-15, 15)

9 BC-BNP

9.1 Prior for the probability of bias

Same approach as the BC parametric:

- We use a Beta(0.5, 1).
- We ignore the direction of bias and we use Uniform(-15, 15) prior.

9.2 Determination of maximum number of clusters and prior for α

Prior for $\alpha \sim \text{Uniform}(0.5, \alpha^{Max})$

$$\alpha^{Max} < \frac{1}{5}\left(\frac{(n-1)\,a_0 - a_1}{a_0 + a_1}\right)$$

The maximum number of clusters in the bias component is:

$$K^{Max} = 1 + 5\,\alpha^{Max}$$

```
# For N=20 studies and the Beta(0.5, 5) we have

a.0.20 = 0.5
a.1.20 = 1

N = 20

alpha.max.20 = 1/5 * ( (N-1)*a.0.20 - a.1.20)/(a.0.20 + a.1.20)
alpha.max.20
```

```
## [1] 1.13333
```

```
# K.max

K.max.20 = 1 + 5*alpha.max.20
K.max.20 = round(K.max.20)
K.max.20
```

```
## [1] 7
```

```
# For N=30 studies and the Beta(0.5, 5) we have

a.0.30 = 0.5
```

```r
a.1.30 = 1

N = 30

alpha.max.30 = 1/5 * ( (N-1)*a.0.30 - a.1.30)/(a.0.30 + a.1.30)
alpha.max.30
```

```
## [1] 1.8
```

```r
# K.max

K.max.30 = 1 + 5*alpha.max.30
K.max.30 = round(K.max.30)
K.max.30
```

```
## [1] 10
```

10 Figures for Section 3

10.1 Comparision of different models N = 20

10.1.1 Bayesian RE

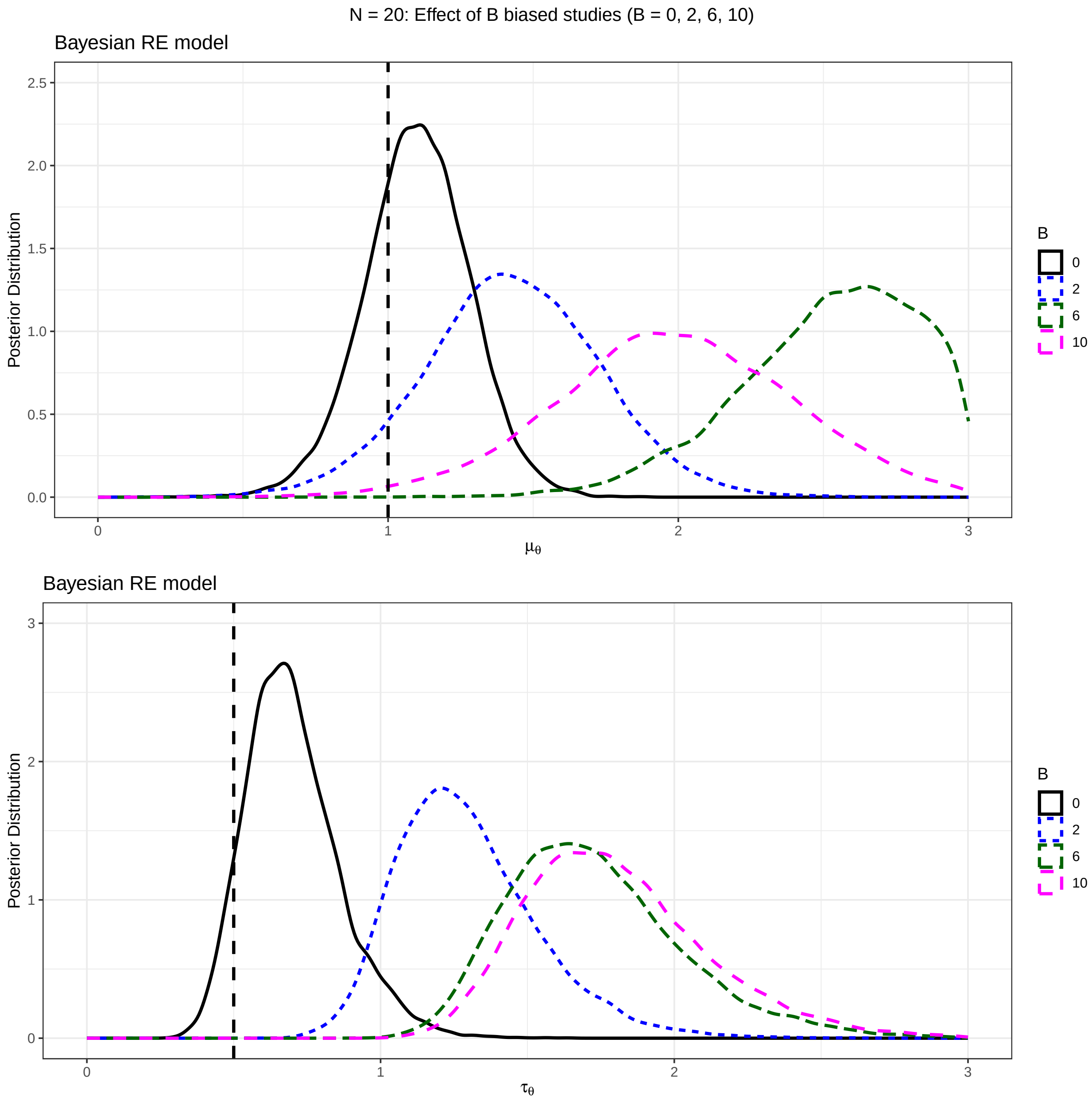

Figure 1: Comparison of the posterior distributions of μ and τ for different number of biased studies.

10.1.2 Oracle Bayesian RE

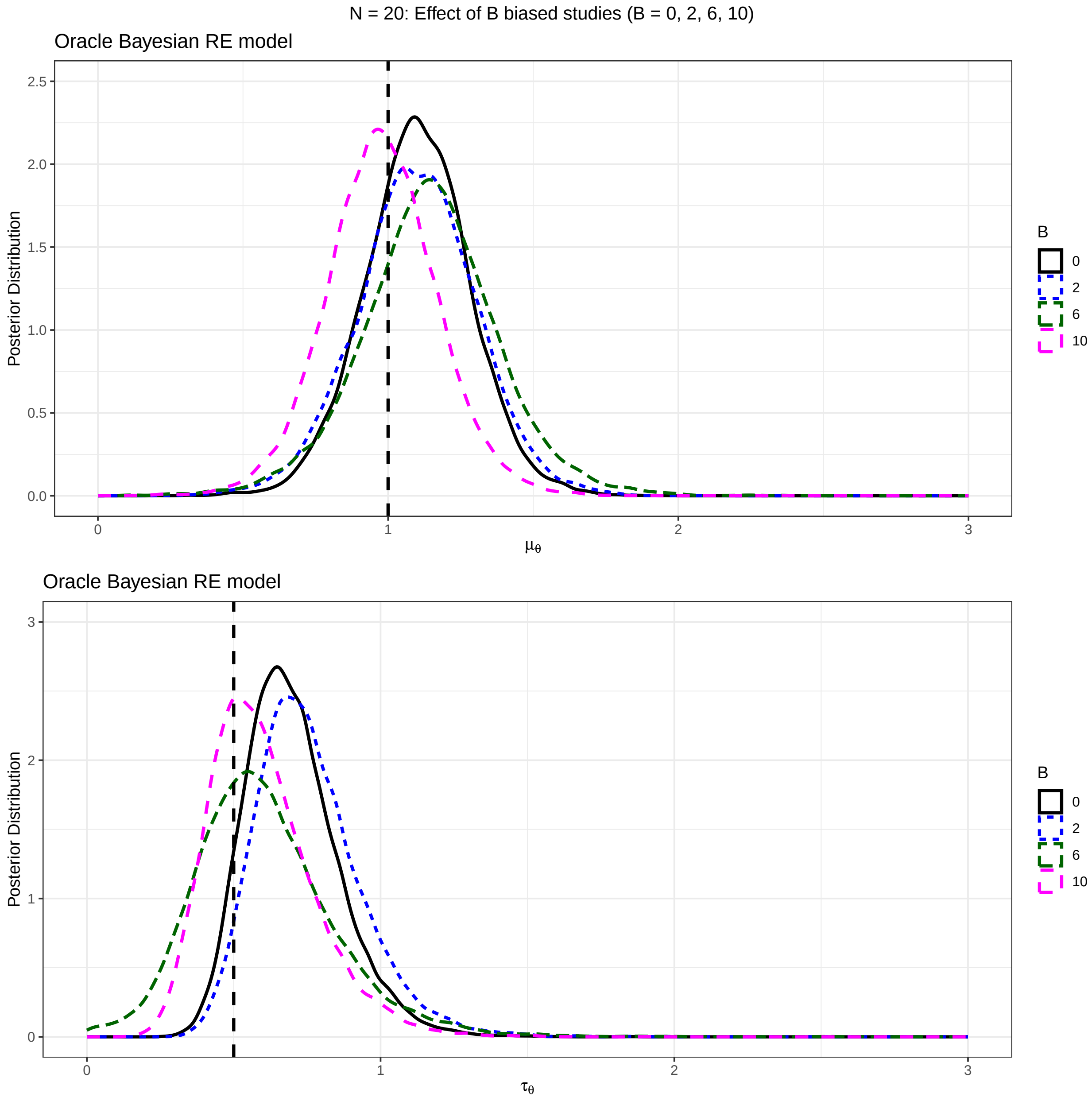

Figure 2: Comparison of the posterior distributions of μ and τ for different number of Bed studies.

10.1.3 BC parametric

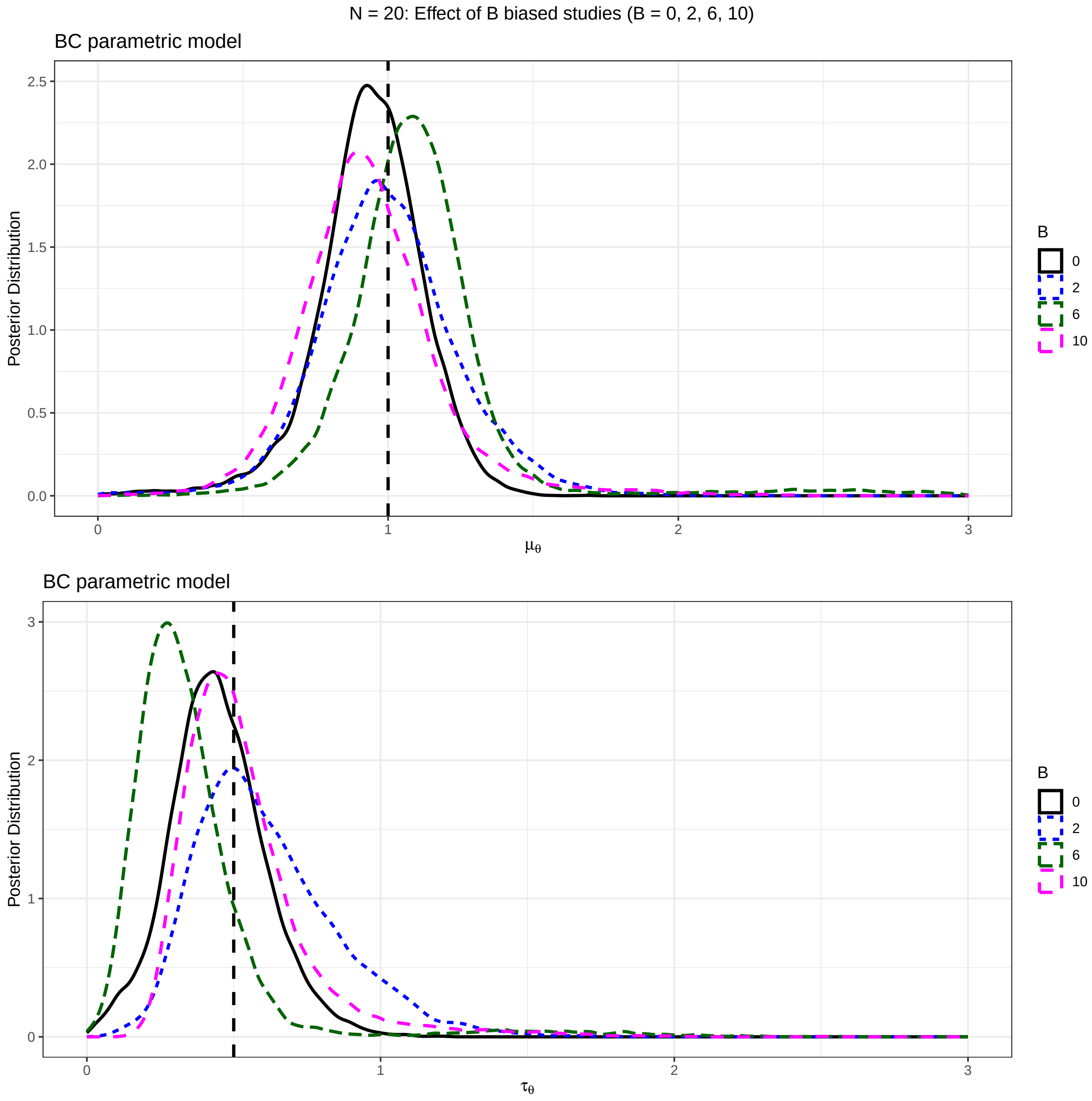

Figure 3: Comparison of the posterior distributions of μ and τ for different number of Bed studies.

10.1.4 BC-BNP

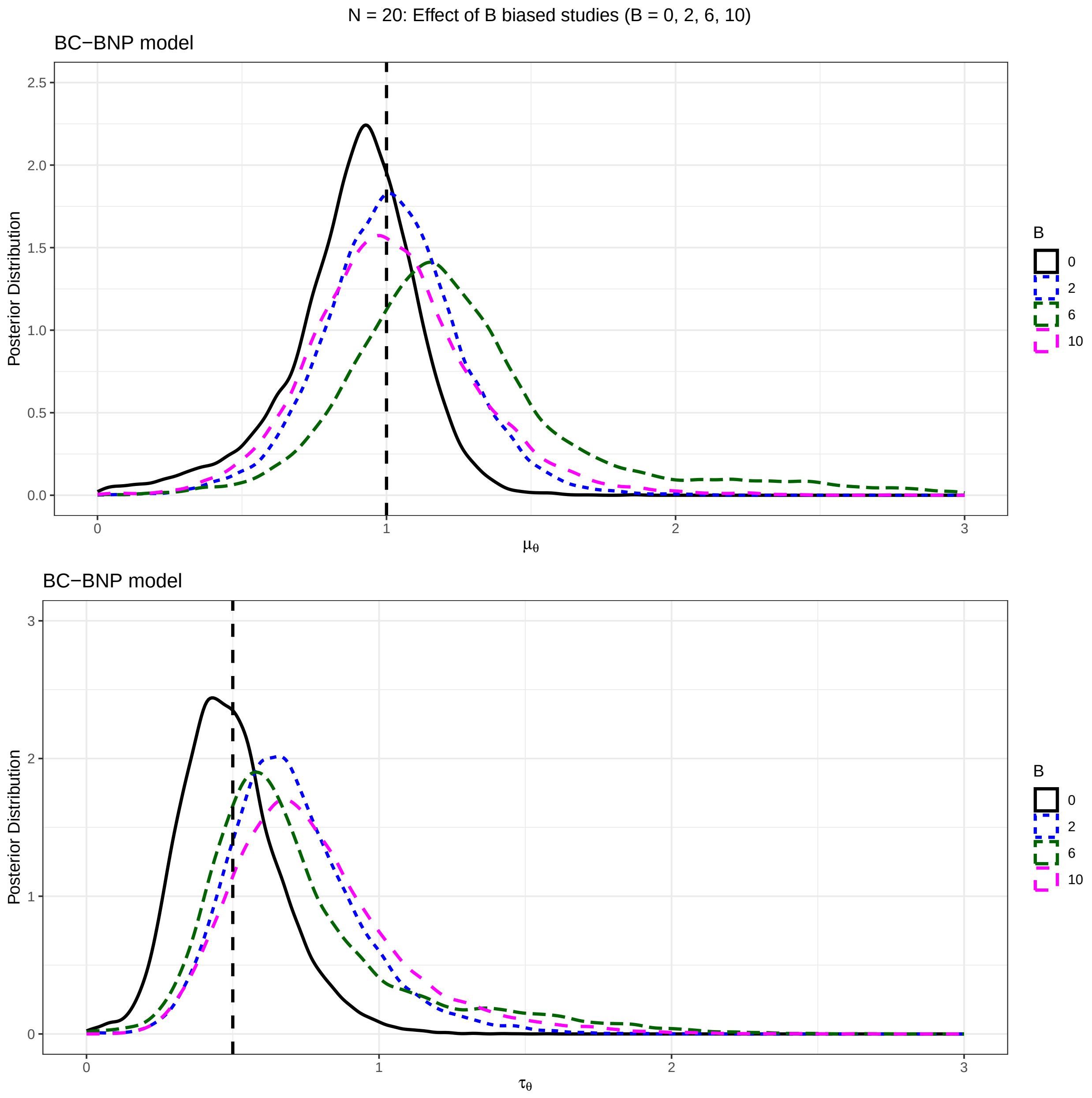

Figure 4: Comparison of the posterior distributions of μ and τ for different number of Bed studies.

10.1.5 All models together

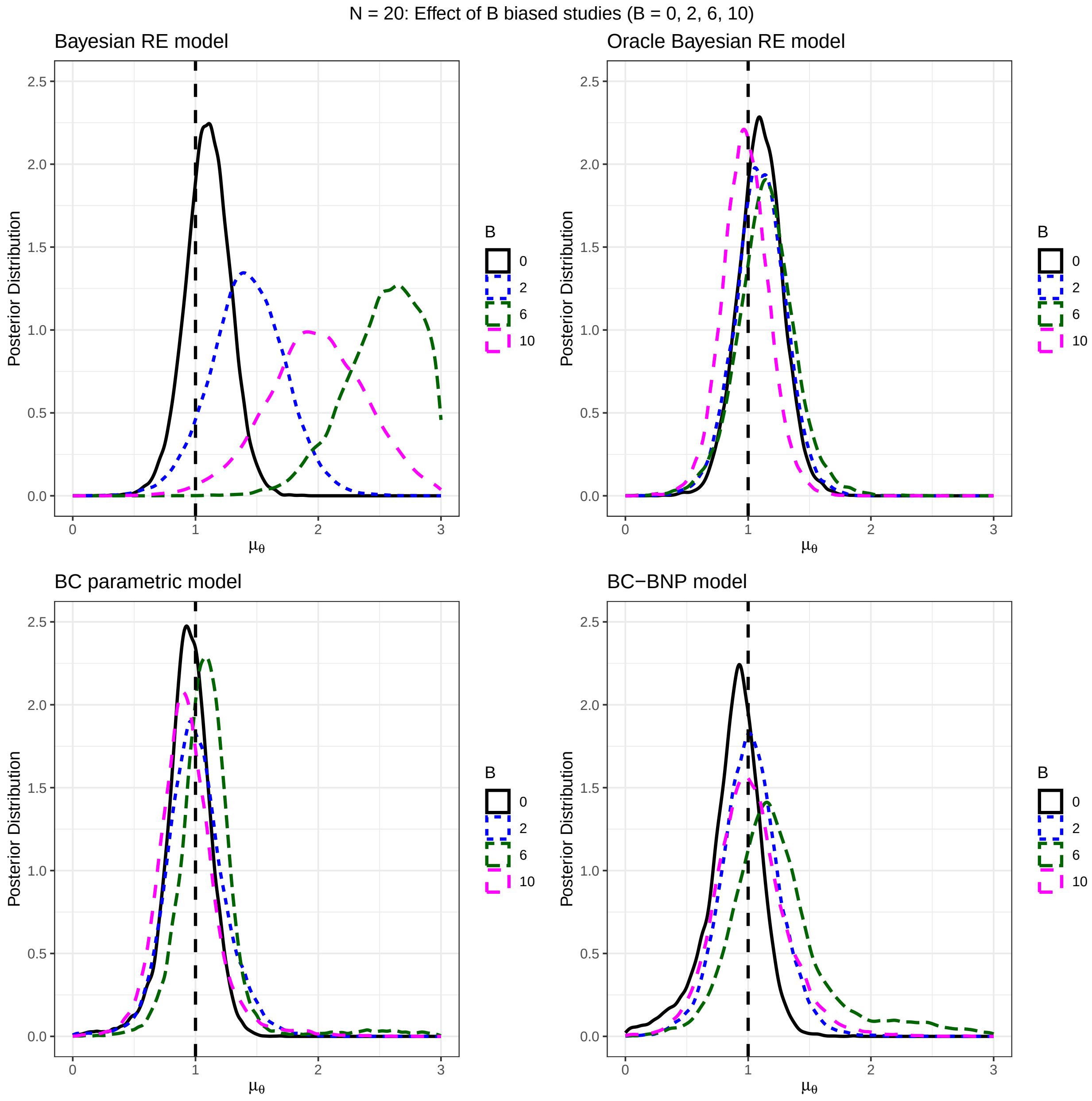

Figure 5: Comparison of the posterior distributions of μ for different number of bias studies.

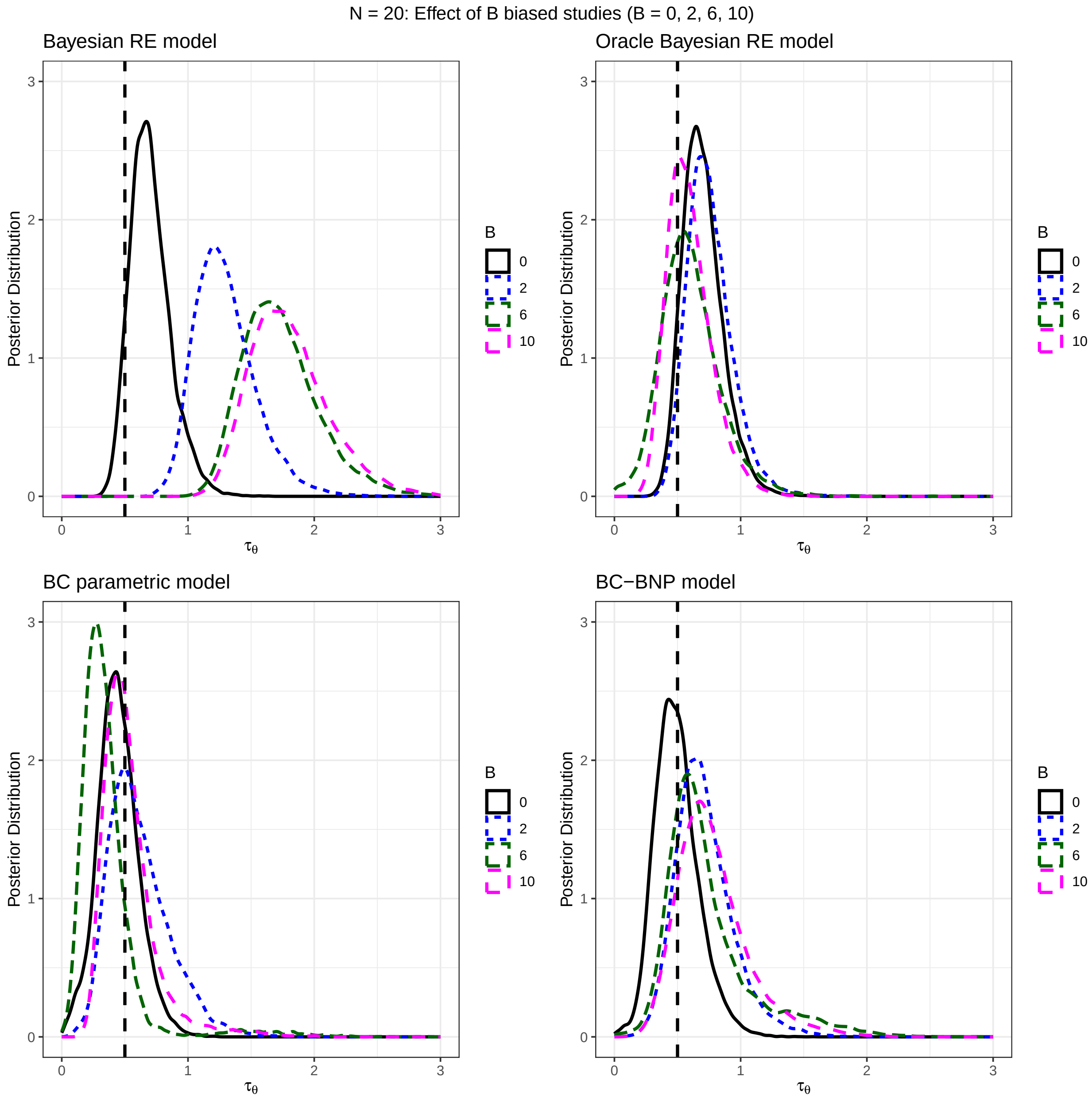

Figure 6: Comparison of the posterior distributions of τ for different number of bias studies.

10.1.6 Forest plot: comparing θ^B and θ

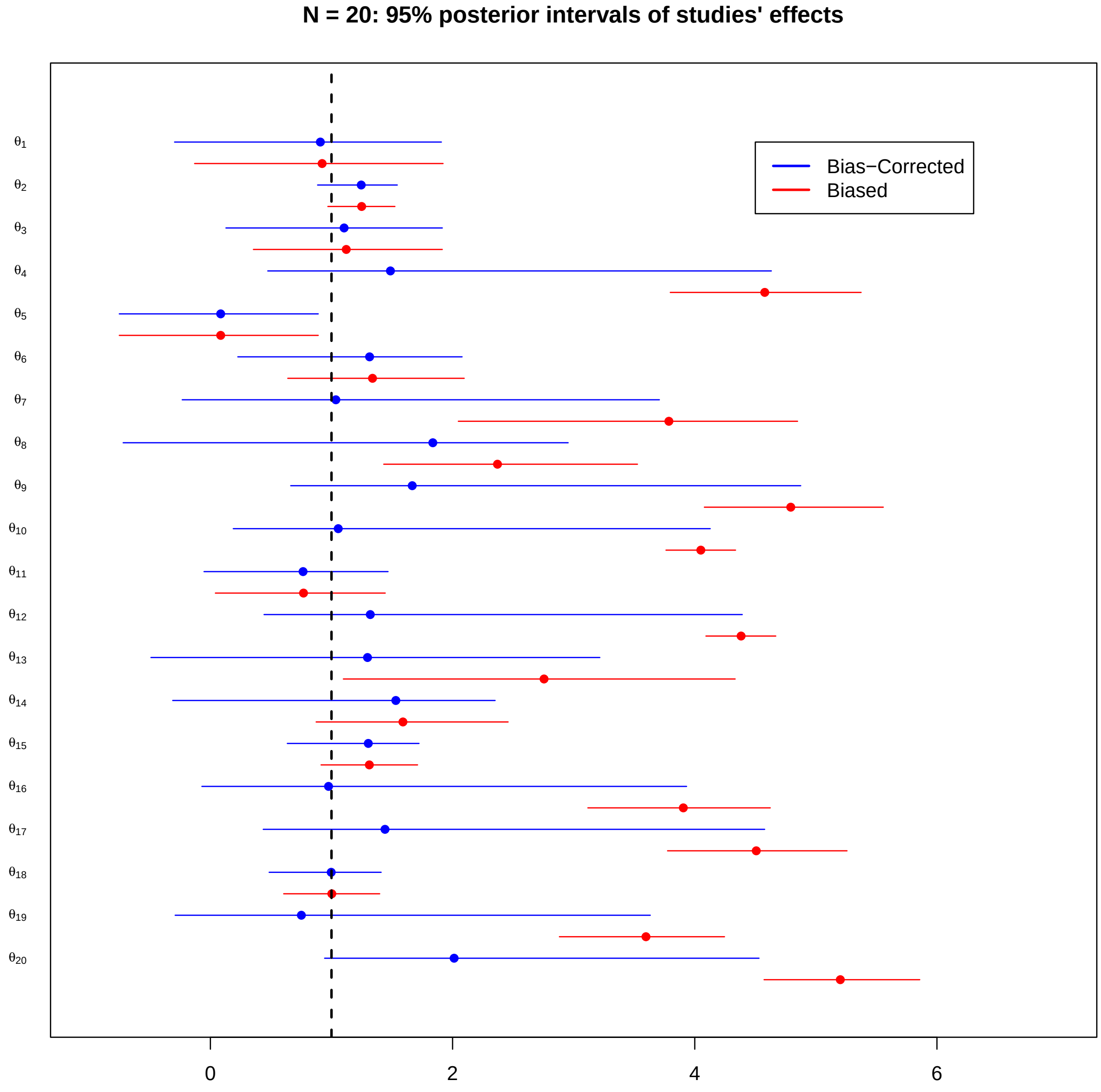

10.1.7 Example of 4 studies: without bias and with bias

```
attach.jags(bcmix.20.30, overwrite = TRUE)

# No bias
dat.theta.1 = data.frame(theta = c(theta.bias[1:1500,1], theta[1:1500,1]),
                         bias = factor(c(rep("biased",1500),
                                         rep("bias corrected", 1500))))

# Large bias
dat.theta.16 = data.frame(theta = c(theta.bias[1:1500,16], theta[1:1500,16]),
                          bias = factor(c(rep("biased",1500),
                                          rep("bias corrected", 1500))))

# Mild bias
dat.theta.19 = data.frame(theta = c(theta.bias[1:1500,19], theta[1:1500,19]),
                          bias = factor(c(rep("biased",1500),
                                          rep("bias corrected", 1500))))

# Extreme bias
dat.theta.20 = data.frame(theta = c(theta.bias[1:1500,20], theta[1:1500,20]),
                          bias = factor(c(rep("biased",1500),
                                          rep("bias corrected", 1500))))

cols <- c("red", "blue")


# Effect on the posterior of theta
theta_1 = ggplot(dat.theta.1, aes(x = theta, color = bias, linetype=bias))+
  geom_density(aes(x = theta), alpha=.2, lwd = 1) +
  scale_color_manual(values = cols) +
        scale_x_continuous(limits = c(-2.1, 8)) +
  scale_y_continuous(limits = c(0, 1.2)) +
        xlab(expression(theta[1])) +
        ylab("Posterior Distribution") +
    geom_vline(aes(xintercept=1), lty = 2, lwd = 1, color = "black")+
     ggtitle("No bias") +
      theme_bw()

theta_16 = ggplot(dat.theta.16, aes(x = theta, color = bias, linetype=bias))+
  geom_density(aes(x = theta), alpha=.2, lwd = 1) +
  scale_color_manual(values = cols) +
        scale_x_continuous(limits = c(-2.1, 8)) +
  scale_y_continuous(limits = c(0, 1.2)) +
        xlab(expression(theta[16])) +
        ylab("Posterior Distribution") +
    geom_vline(aes(xintercept=1), lty = 2, lwd = 1, color = "black")+
        theme_bw() + ggtitle("Large bias")


theta_19 = ggplot(dat.theta.19, aes(x = theta, color = bias, linetype=bias))+
  geom_density(aes(x = theta), alpha=.2, lwd = 1) +
  scale_color_manual(values = cols) +
        scale_x_continuous(limits = c(-2.1, 8)) +
```

```
  scale_y_continuous(limits = c(0, 1.2)) +
        xlab(expression(theta[19])) +
        ylab("Posterior Distribution") +
    geom_vline(aes(xintercept=1), lty = 2, lwd = 1, color = "black")+
      theme_bw() + ggtitle("Mild bias")


theta_20 = ggplot(dat.theta.20, aes(x = theta, color = bias, linetype=bias))+
  geom_density(aes(x = theta), alpha=.2, lwd = 1) +
  scale_color_manual(values = cols) +
        scale_x_continuous(limits = c(-2.1, 8)) +
  scale_y_continuous(limits = c(0, 1.2)) +
        xlab(expression(theta[20])) +
        ylab("Posterior Distribution") +
    geom_vline(aes(xintercept=1), lty = 2, lwd = 1, color = "black")+
      theme_bw() + ggtitle("Extreme bias")

grid.arrange(theta_1, theta_16, theta_19, theta_20,
             ncol = 2,  nrow = 2, top = "N = 20: Effect of bias correction")
```

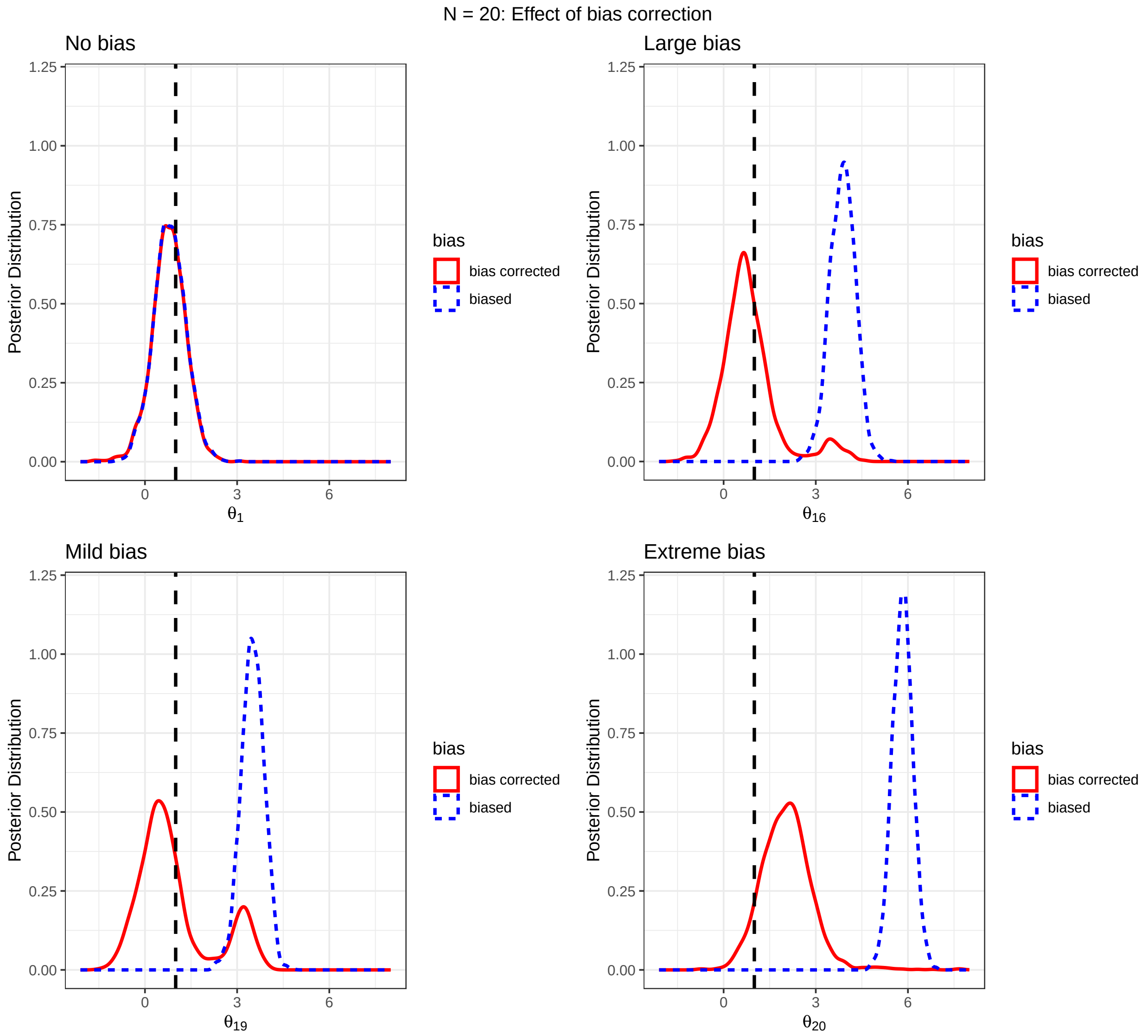
N = 20: Effect of bias correction
No bias
Large bias
Mild bias
Extreme bias
Posterior Distribution
1.25
1.00
0.75
0.50
0.25
0.00
0
3
6
θ_1
θ_{16}
θ_{19}
θ_{20}
bias
bias corrected
biased

10.2 Summary of BC-BNP for N=20 and B=6

These are the posteriors of I_i for studies number 1, 19, 16, and 20.

```
attach.jags(bcmix.20.50, overwrite = TRUE)
round(apply(I[,c(1, 16, 19, 20)], 2, mean), 2)
```

```
## [1] 0.04 0.92 0.89 1.00
```

10.3 Comparison of different models N = 30

10.3.1 Bayesian RE

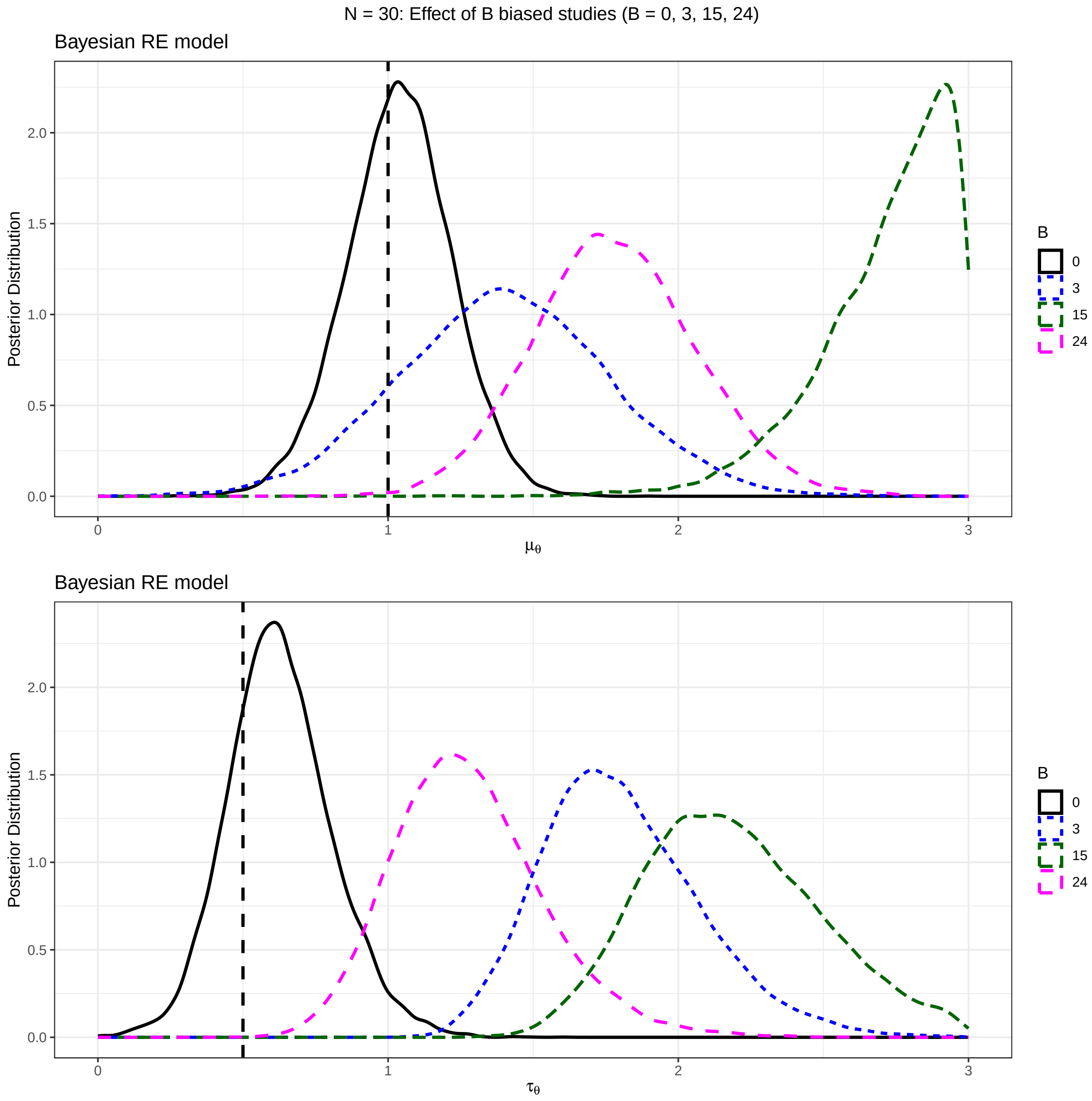

Figure 7: Comparison of the posterior distributions of μ and τ for different number of Bed studies.

10.3.2 Oracle Bayesian RE

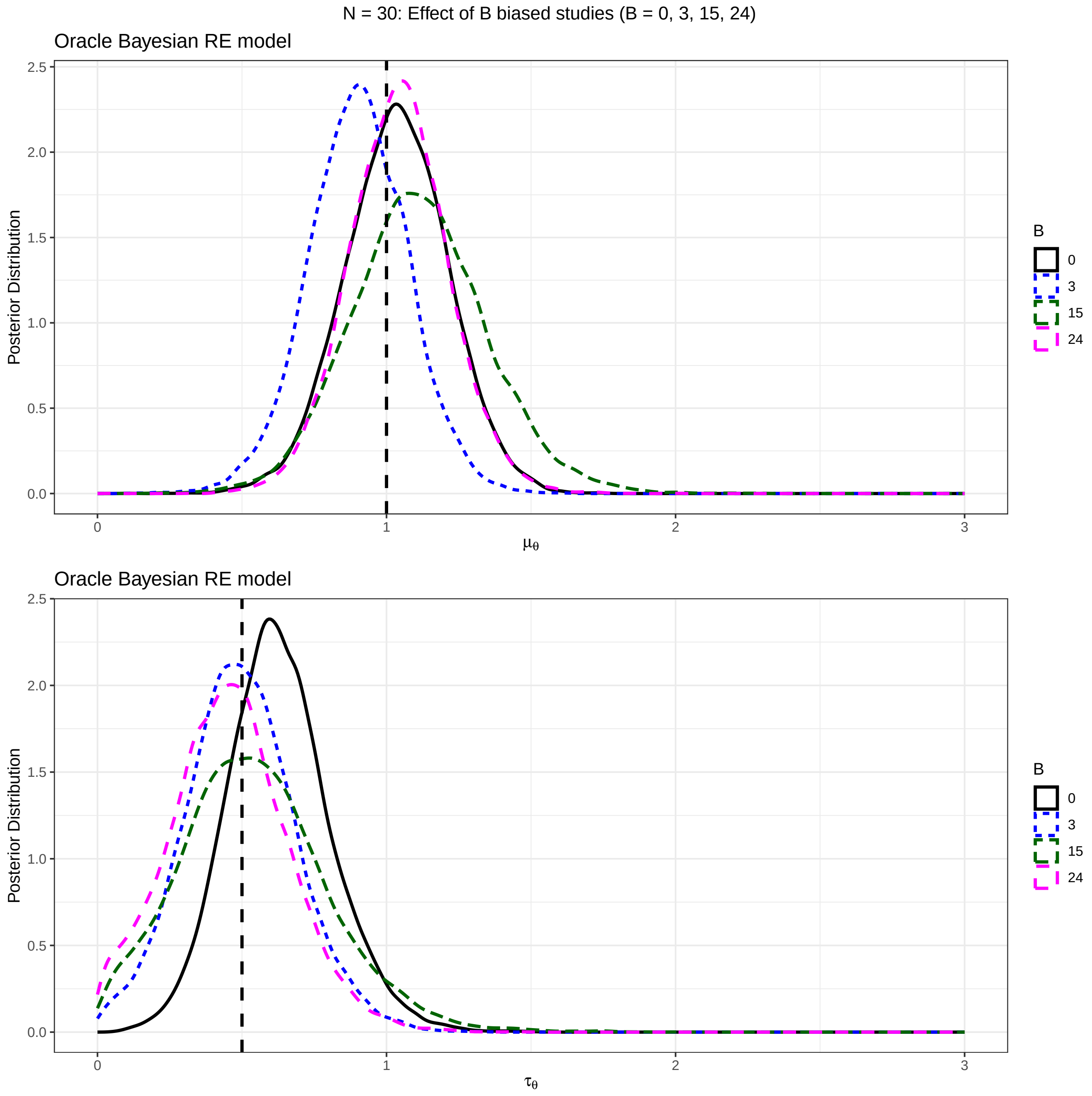

Figure 8: Comparison of the posterior distributions of μ and τ for different number of Bed studies.

10.3.3 BC parametric

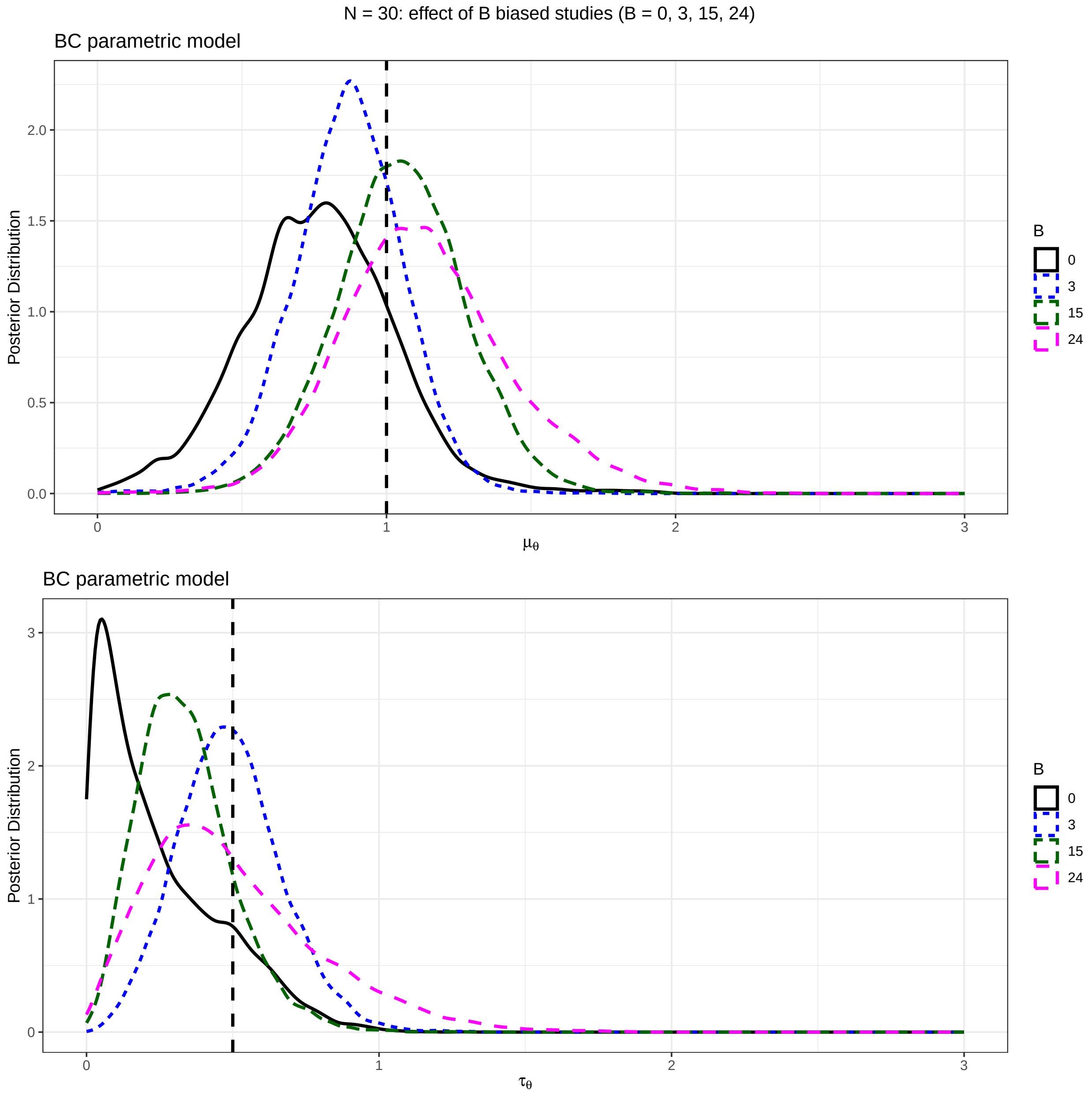

Figure 9: Comparison of the posterior distributions of μ and τ for different number of Bed studies.

10.3.4 BC-BNP

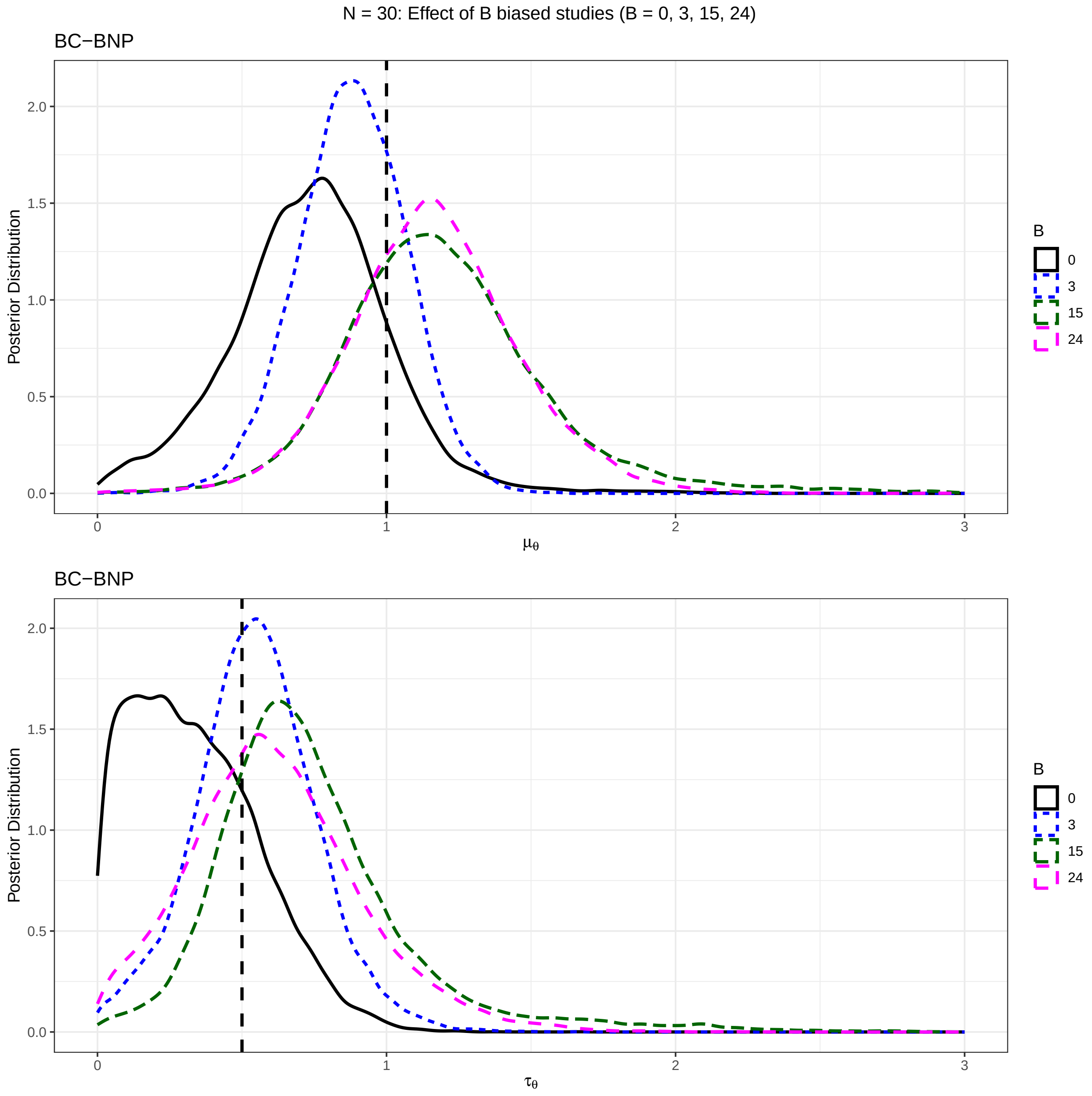

Figure 10: Comparison of the posterior distributions of μ and τ for different number of Bed studies.

10.3.5 All models together

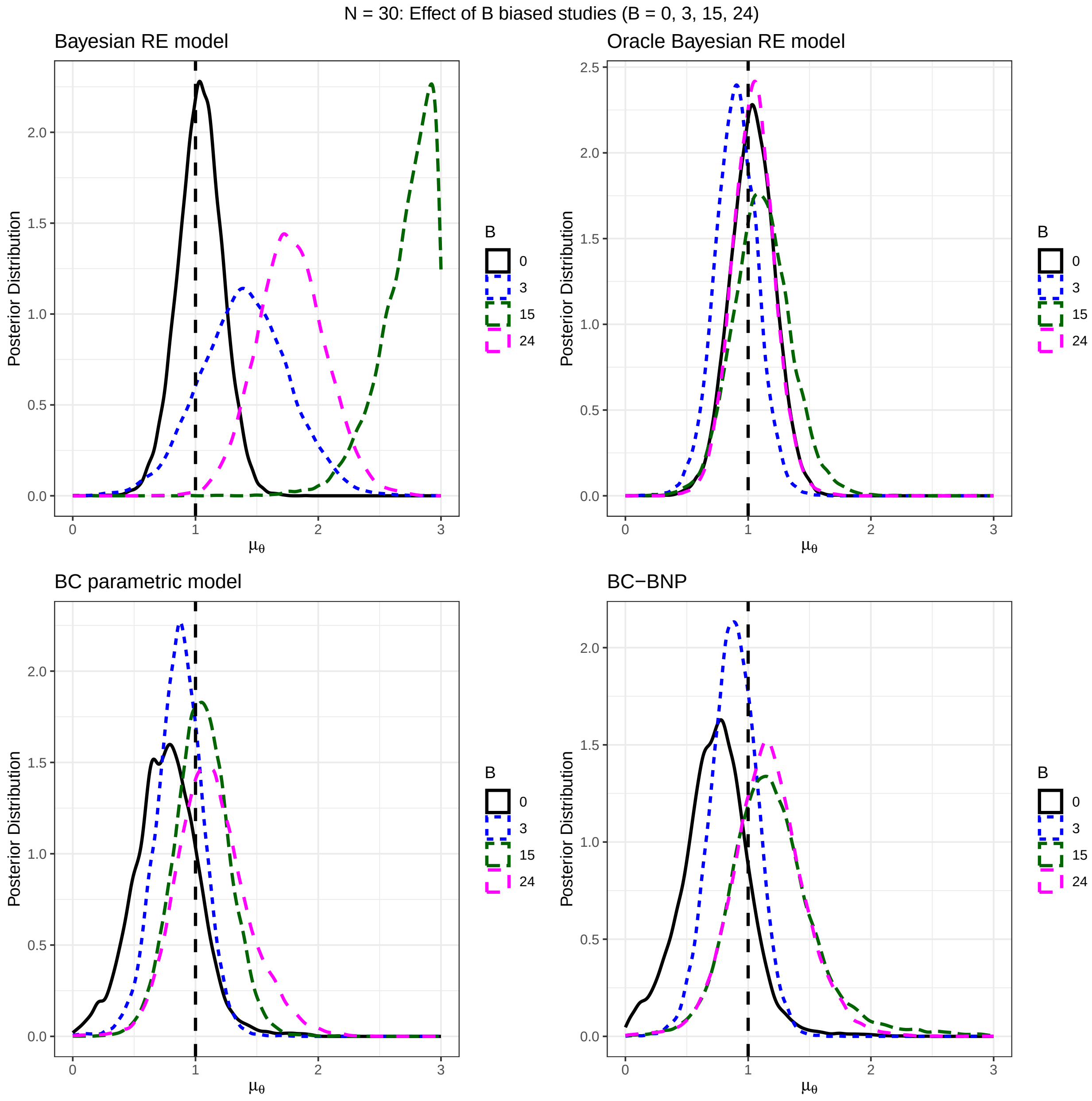

Figure 11: Comparison of the posterior distributions of μ for different number of bias studies.

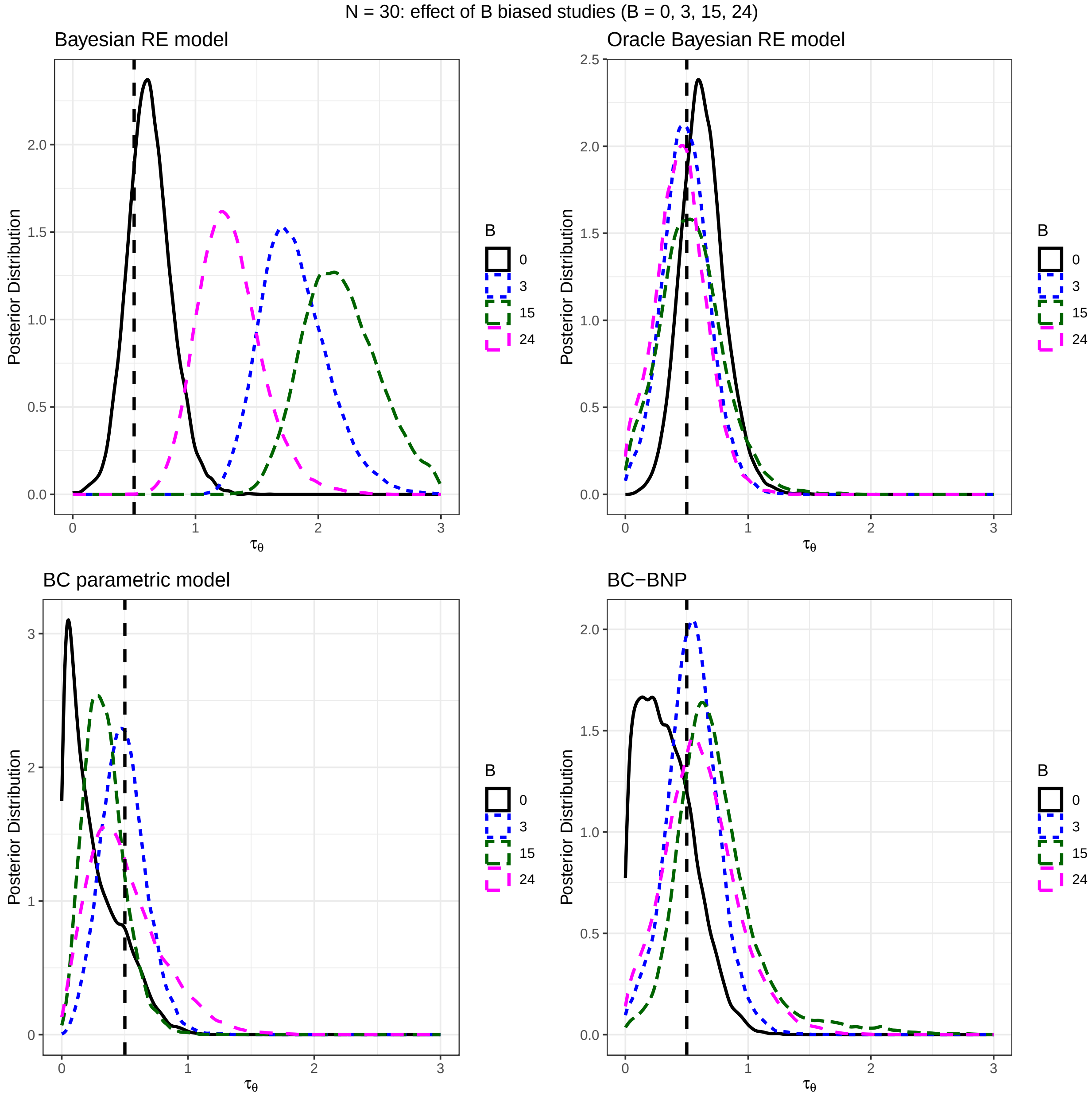

Figure 12: Comparison of the posterior distributions of τ for different number of bias studies.

11 Clusters report n=20 with 50% bias

11.1 Co-clustering Posterior probability study i shares the same cluster with j

- In the JAGS code we monitor: **equalsmatrix[i,j] <- equals(group[i], group[j])**, which gives, for each MCMC iteration, the number of times that each study "i" has the same cluster's label as cluster "j".
- We average this $N \times N$ matrix over the MCMC iterations.
- We display a cluster analysis to describe the results.

```
require(pheatmap)
```

```
## Loading required package: pheatmap
```

```
require(RColorBrewer)
```

```
## Loading required package: RColorBrewer
```

```
attach.jags(bcmix.20.50, overwrite = TRUE)
distance.studies.2 = apply(equalsmatrix.bias.2, c(2,3), mean)
colnames(distance.studies.2) = rownames(sim.20.50)

rownames(distance.studies.2)= paste(paste("Study",1:20, sep = "-"),
                                    sim.20.50$B.flag, sep=" | ")
colnames(distance.studies.2)= round(apply(I, 2, mean),3)

#distance.studies.2

p = pheatmap(distance.studies.2,color=brewer.pal(9,"Blues"), fontsize_row =7,
              fontsize_col = 6, angle_col =45,
              treeheight_row=0,
              treeheight_col=0,
              clustering_distance_cols = as.dist(1 - distance.studies.2),
              clustering_distance_rows = as.dist(1 - distance.studies.2),
             )
p
```

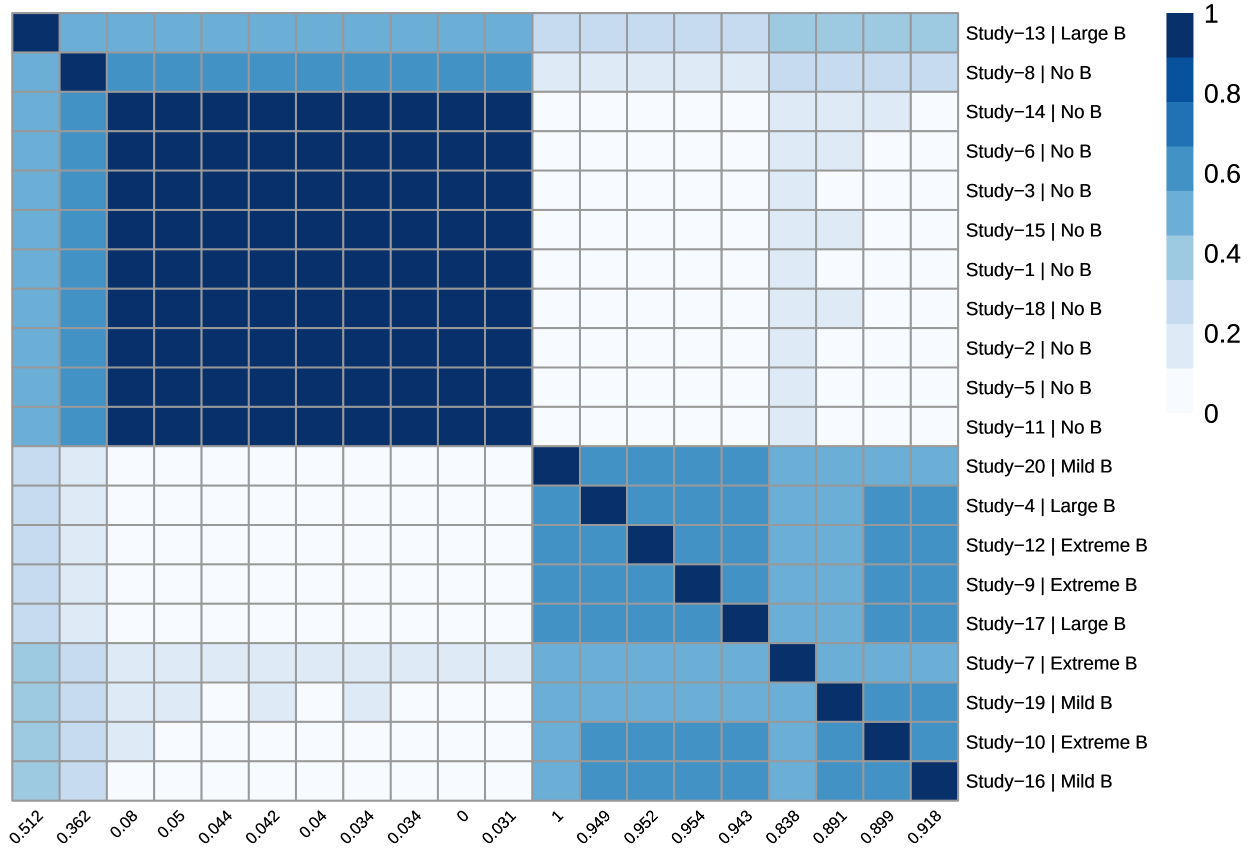

Figure 13: Heatmap based the co-clustering between studies. The row labels correspond to the level of bias. The column labels correspond to the probability of bias.

12 Clusters report n=30 with 50% bias

12.1 Co-clustering Posterior probability study i shares the same cluster with j

```
require(pheatmap)
require(RColorBrewer)

attach.jags(bcmix.30.50, overwrite = TRUE)
distance.studies.2 = apply(equalsmatrix.bias.2, c(2,3), mean)

colnames(distance.studies.2)= round(apply(I, 2, mean),3)
rownames(distance.studies.2)= sim.30.50$B.flag

#distance.studies.2

pheatmap(distance.studies.2,color=brewer.pal(9,"Blues"), fontsize_row =7,
              fontsize_col = 6, angle_col =45,
              treeheight_row=0,
              treeheight_col=0,
              clustering_distance_cols = as.dist(1 - distance.studies.2),
              clustering_distance_rows = as.dist(1 - distance.studies.2))
```

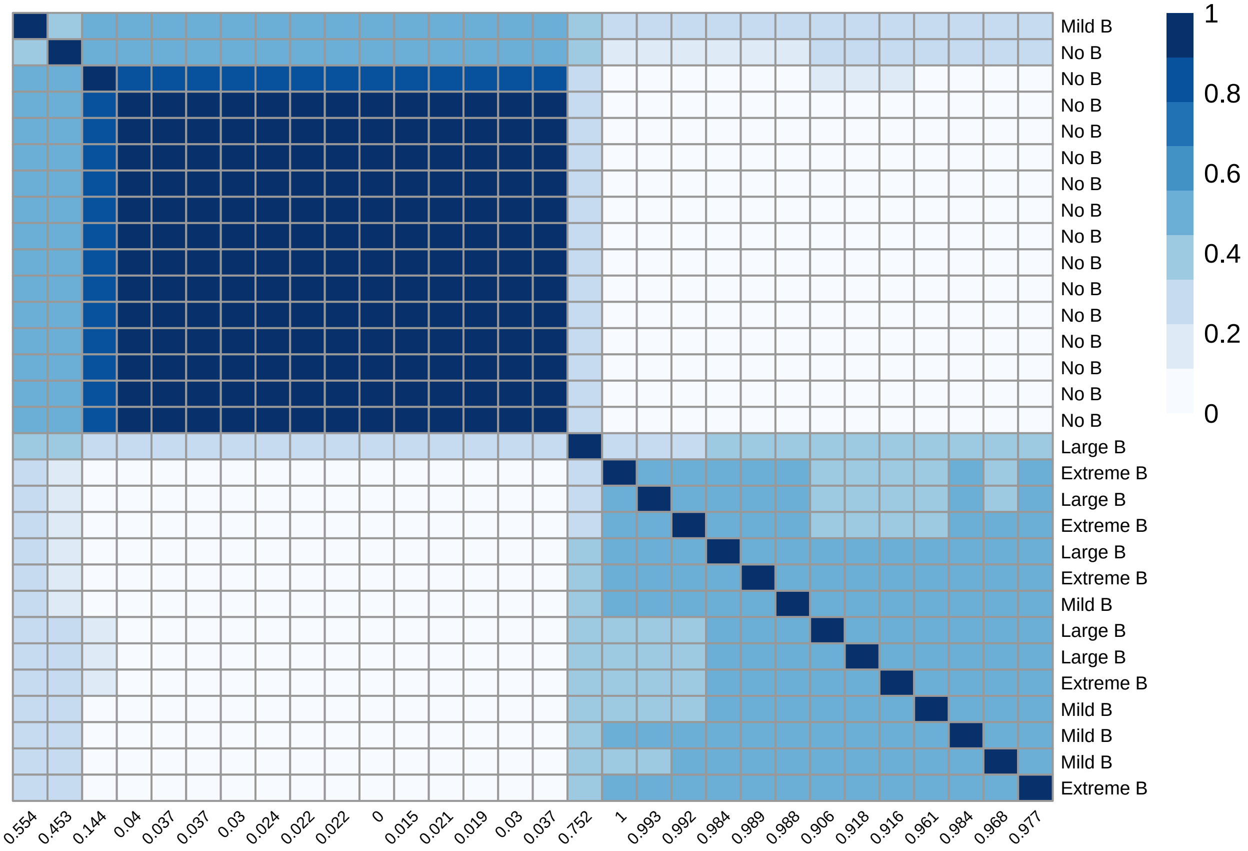

Figure 14: Heatmap based the co-clustering between studies. The row labels correspond to the level of bias (no bias, mild, large, and extreme). The column labels correspond to the probability of bias.

```r
time.end = Sys.time()
```

```r
#Total time
difference = difftime(time.end, time.start, units='mins')
difference
```

```
## Time difference of 31.9191 mins
```

13 Session information

```r
sessionInfo()
```

```
## R version 4.4.2 (2024-10-31 ucrt)
## Platform: x86_64-w64-mingw32/x64
## Running under: Windows 11 x64 (build 22631)
##
## Matrix products: default
##
##
## locale:
## [1] LC_COLLATE=German_Germany.utf8  LC_CTYPE=German_Germany.utf8
## [3] LC_MONETARY=German_Germany.utf8 LC_NUMERIC=C
## [5] LC_TIME=German_Germany.utf8
##
## time zone: Europe/Berlin
## tzcode source: internal
##
## attached base packages:
## [1] grid      stats     graphics  grDevices utils     datasets  methods
## [8] base
##
## other attached packages:
##  [1] RColorBrewer_1.1-3 pheatmap_1.0.12    statip_0.2.3       bspmma_0.1-2
##  [5] extraDistr_1.10.0  meta_8.0-1         metadat_1.2-0      jarbes_2.2.3
##  [9] GGally_2.2.1       R2jags_0.8-9       rjags_4-16         mcmcplots_0.4.3
## [13] coda_0.19-4.1      gridExtra_2.3      lubridate_1.9.3    forcats_1.0.0
## [17] stringr_1.5.1      dplyr_1.1.4        purrr_1.0.2        readr_2.1.5
## [21] tidyr_1.3.1        tibble_3.2.1       ggplot2_3.5.1      tidyverse_2.0.0
##
## loaded via a namespace (and not attached):
##  [1] tidyselect_1.2.1    farver_2.1.2        fastmap_1.2.0
##  [4] denstrip_1.5.4      CompQuadForm_1.4.3  mathjaxr_1.6-0
##  [7] promises_1.3.0      rpart_4.1.23        digest_0.6.37
## [10] timechange_0.3.0    mime_0.12           lifecycle_1.0.4
## [13] cluster_2.1.6       magrittr_2.0.3      compiler_4.4.2
## [16] rlang_1.1.4         tools_4.4.2         utf8_1.2.4
## [19] yaml_2.3.10         knitr_1.49          labeling_0.4.3
## [22] plyr_1.8.9          xml2_1.3.6          abind_1.4-8
## [25] miniUI_0.1.1.1      withr_3.0.2         numDeriv_2016.8-1.1
## [28] fansi_1.0.6         xtable_1.8-4        colorspace_2.1-1
## [31] scales_1.3.0        MASS_7.3-61         cli_3.6.3
## [34] rmarkdown_2.29      metafor_4.6-0       generics_0.1.3
## [37] rstudioapi_0.17.1   tzdb_0.4.0          minqa_1.2.8
```

```
## [40] splines_4.4.2       parallel_4.4.2      vctrs_0.6.5
## [43] boot_1.3-31         Matrix_1.7-1        hms_1.1.3
## [46] clue_0.3-66         R2WinBUGS_2.1-22.1  glue_1.8.0
## [49] nloptr_2.1.1        ggstats_0.7.0       stringi_1.8.4
## [52] gtable_0.3.6        later_1.3.2         sfsmisc_1.1-20
## [55] lme4_1.1-35.5       munsell_0.5.1       pillar_1.9.0
## [58] htmltools_0.5.8.1   ggExtra_0.10.1      R6_2.5.1
## [61] evaluate_1.0.1      shiny_1.9.1         lattice_0.22-6
## [64] httpuv_1.6.15       Rcpp_1.0.13-1       nlme_3.1-166
## [67] xfun_0.49           pkgconfig_2.0.3
```
